# Supplementary material for: Structure, function and substrate preferences of archaeal S-adenosyl-l-homocysteine hydrolases
Source: Commun Biol. 2024 Mar 29;7:380. doi: 10.1038/s42003-024-06078-9 (PMC10978960; doi:10.1038/s42003-024-06078-9)
Supplement: Supplementary file 2 — Supplementary Information [file 42003_2024_6078_MOESM2_ESM.pdf]

Electronic Supplementary Information

Structure, function and substrate preferences of archaeal *S*-adenosyl-L-homocysteine hydrolases

Lars-Hendrik Koeppl[1,+], Désirée Popadić[1,+], Raspudin Saleem-Batcha[1,+], Philipp Germer[1], Jennifer N. Andexer[1]*

[1]Institute of Pharmaceutical Sciences, University of Freiburg, Albertstr. 25, 79104 Freiburg, Germany
*****Corresponding E-mail: jennifer.andexer@pharmazie.uni-freiburg.de

[+] These authors contributed equally

Table of Contents

Experimental 2

HPLC analysis 4

Crystallography 5

Bioinformatical results 8

SIH enzymatic synthesis and structure verification 10

SAHH/SIHH characterisation 13

Supplementary Notes 1 49

Supplementary Notes 2 57

References 61

Experimental

Table S1. The primers used for cloning of SAHHs/SIHHs and their molecular weight including His_6-tag.

Enzyme	Forward primer (5'–3')	Reverse primer (5'–3')	Molecular weight [kDa]	Reference
*Cg*SAHH	TATATATACATATGGCACAGG TTATGGACTTC	TATATATACTCGAGTTAGTAG CGGTAGTGCTC	54.59	This work
*Ll*SAHH	TATATATACATATGGCCCTGC TGGTTG	TATATATAAAGCTTTTAGTAG CGATAATGAAACG	55.49	Guranowski and Pawelkiewicz, 1977[1]; Brzezinski *et al.,* 2008[2] and 2012[3]
*Mc*SAHH*			48.07	This work
*Me*SAHH*			54.04	This work
*Mh*SAHH*			53.97	This work
*Mi*SAHH*			49.49	This work
*Mj*SIHH	TATATATACATATGTACGAGG TGCGC	TATATATACTCGAGTTAGGTG CCTTC	48.77	Miller *et al*., 2015[4]
*Mma*SAHH	CGCGCGGCAGCCATATGAGC AACGTGAAAGATATGAGCC	GTGCGGCCGCAAGCTTAGGT GCCTTCTTTCCAATCGC	48.00	This work
*Mm*SAHH	CGCGCGGCAGCCATATGAGC GACAAACTGCCGTATAAAG	GTGCGGCCGCAAGCTAAGCT TAATAGCGATAATGATCCGG	49.85	Ishihara *et al*., 2010[5]; Kusakabe *et al*., 2015[6]
*Mt*SAHH*			48.51	This work
*Pa*SAHH	TATATATACATATGAGCGCAG TTATGACAC	TATATATAAAGCTTTTAATAG CGATAGGTATCC	53.56	Czyrko *et al.,* 2018[7]
*Pfu*SAHH	CGCGCGGCAGCCATATGGAT TGCGGCAAAGATTATTG	GTGCGGCCGCAAGCTTAGGT GCCATGTTCCCAAC	49.55	Porcelli *et al*., 2005[8]
*Sa*SAHH	TATATATACATATGGCAAGCG CCCAGCA	TATATATAAGCTTCAGTACCG GTAGTGGTC	54.72	This work
*Sac*SAHH	TATACATATGGATTATCGCGT TAAAGATCTG	TATATAAGCTTAGGTGCCGTA TTTCCACTG	48.34	This work

*Plasmids encoding the enzymes were ordered from BioCat GmbH (Heidelberg, Germany).

Enzyme	Forward primer (5'–3')	Reverse primer (5'–3')	Molecular weight [kDa]	Reference
*Sf*SAHH	TATACATATGACGACGACCTC CACGAC	TATAAGCTTTCAGTAGCGGTA GTGG	54.87	This work
*Sso*SAHH	CGCGCGGCAGCCATATGAGC TACAAAATCAAAGATCTGAG	GTGCGGCCGCAAGCTTAGGT GCCGCTTTTCCACTG	48.13	Porcelli *et al.*, 1993[9] and 2000[10]
*Tk*SAHH	CGCGCGGCAGCCATATGGAC TGCACGAAGGATT	GTGCGGCCGCAAGCTAAGCT TCAGGTGCCGTGCTCCCAGC	49.04	This work
*Tm*SAHH	TATATATACATATGAACACCG GTGAGA	TATATATACTCGAGTTACTGC CAGCTA	47.01	Hermann *et al.*, 2007[11]; Lozada-Ramírez *et al.*, 2013[12]

Table S2. Enzymes used in this study with their UniProt accession numbers [1–12].

Enzyme	Organism	Domain	Phylum/Kingdom	UniProt accession number
*Sac*SAHH	*Sulfolobus acidocaldarius*	Archaea	Crenarchaeota	Q4JAZ7 (SAHH_SULAC)
*Sso*SAHH	*Saccharolobus solfataricus*	Archaea	Crenarchaeota	P50252 (SAHH_SACS2)
*Mc*SAHH	*Methanocella conradii*	Archaea	Euryarchaeota	H8I6W9 (H8I6W9_METCZ)
*Me*SAHH	*Methanohalobium evestigatum*	Archaea	Euryarchaeota	D7E778 (D7E778_METEZ)
*Mh*SAHH	*Methanohalophilus halophilus*	Archaea	Euryarchaeota	A0A1L3Q075 (A0A1L3Q075_9EURY)
*Mi*SAHH	*Methanocaldococcus infernus*	Archaea	Euryarchaeota	D5VRX5 (D5VRX5_METIM)
*Mj*SIHH	*Methanocaldococcus jannaschii*	Archaea	Euryarchaeota	Q58783 (SIHH_METJA)
*Mma*SAHH	*Methanococcus maripaludis*	Archaea	Euryarchaeota	Q6LYR8 (SIHH_METMP)
*Mt*SAHH	*Methanothrix thermoacetophila*	Archaea	Euryarchaeota	A0B7W5 (A0B7W5_METTP)
*Pfu*SAHH	*Pyrococcus furiosus*	Archaea	Euryarchaeota	P50251 (SAHH_PYRFU)
*Tk*SAHH	*Thermococcus kodakarensis*	Archaea	Euryarchaeota	Q5JED2 (SAHH_THEKO)
*Cg*SAHH	*Corynebacterium glutamicum*	Bacteria	Actinomycetota	Q8NSC4 (SAHH_CORGL)
*Pa*SAHH	*Pseudomonas aeruginosa*	Bacteria	Pseudomonadota	Q9I685 (SAHH_PSEAE)

Enzyme	Organism	Domain	Phylum/Kingdom	UniProt accession number
*Sa*SAHH	*Streptomyces albus*	Bacteria	Actinomycetota	Not assigned
*Sf*SAHH	*Streptomyces flocculus*	Bacteria	Actinomycetota	Not assigned
*Tm*SAHH	*Thermotoga maritima*	Bacteria	Thermotogota	O51933 (SAHH_THEMA)
*Ll*SAHH	*Lupinus luteus*	Eukaryota	Plantae	Q9SP37 (SAHH_LUPLU)
*Mm*SAHH	*Mus musculus*	Eukaryota	Animalia	P50247 (SAHH_MOUSE)

HPLC analysis

Table S3. HPLC retention times.

Substance	Retention time [min]
Adenine	9.2
Adenosine	7.9
Hypoxanthine	3.4
Inosine	2.7
SAH	8.0
SIH	3.0

Crystallography

Table S4. Overview of SAHHs with solved structures deposited in the PDB databank.

Organism	Domain, Phylum/Kingdom	PDB-ID	Bound Molecules	Reference
Acanthamoeba castellanii	Eukaryota, Amoebozoa	6UK3	Adenosine	Unpublished
Bradyrhizobium elkanii	Bacteria, Proteobacteria	4LVC	Adenosine	Manszewski *et al*., 2015[13]
		5M65<br>5M66<br>5M67<br>5M5K	Adenine<br>Adenosine<br>Adenine, 2´-deoxyadenosine<br>Adenosine, Cordycepin	Manszewski *et al*., 2017[14]
		6EXI	NAD^+-free, Adenosine	Kailing *et al*., 2018[15]
Brucella abortus	Bacteria, Proteobacteria	3N58	Adenosine	Unpublished
Burkholderia pseudomallei	Bacteria, Proteobacteria	3D64<br>3GLQ	None<br>9-β-D-arabinofuranosyladenine	Unpublished
Cryptosporidium parvum	Eukaryota, Apicomplexa	5HM8<br>5T8K<br>5TJ9<br>5TLS<br>5UTU	Adenosine<br>Adenine<br>Aristeromycin<br>DZ2002<br>SAH, Adenosine	Unpublished
Elizabethkingia anophelis	Bacteria, Bacteroidetes	6APH	Adenosine	Unpublished
Homo sapiens	Eukaryota, Animalia	1A7A	Adenosine analogue	Turner *et al*., 1998[16]
		1LI4	Neplanocin	Yang *et al*., 2003[17]
		3GVP	None	Unpublished
		3NJ4	Fluoro-neplanocin A	Lee *et al*., 2011[18]
		4YVF	Complex Isoindoline/chloroaniline inhibitor	Nakao *et al*., 2015[19]
		5W4B<br>5W49	Benzothiazole inhibitor<br>Oxadiazole inhibitor	Uchiyama *et al* ., 2017[20]
		4PGF<br>4PFJ	Adenosine (mono-acetylated protein<br>Adenosine (bi-acetylated protein)	Wang *et al*., 2014[21]
Leishmania major	Eukaryota, Euglenozoa	3G1U	Adenosine	Unpublished
Lupinus luteus	Eukaryota, Plantae	3OND<br>3ONE<br>3ONF	Adenosine<br>Adenine<br>3´-deoxyadenosine	Brzezinski *et al*., 2012[3]
Mus musculus	Eukaryota, Animalia	5AXA<br>5AXB<br>5AXC<br>5AXD	Adenosine<br>Noraristeromycin<br>3´-keto aristeromycin<br>Ribavirin	Kusakabe *et al*., 2015[6]
Mycobacterium tuberculosis	Bacteria, Actinobacteria	2ZIZ<br>2ZJ0	3-deazaadenosine<br>2-fluoroadenosine	Reddy *et al*., 2008[22]

		2ZJ1<br>3CE6<br>3DHY	3´-keto aristeromycin<br>Adenosine<br>5´-ethylthioadenosine	
Naegleria fowleri	Eukaryota, Percolozoa	5V96	Adenosine	Unpublished
Plasmodium falciparum	Eukaryota, Apicomplexa	1V8B	Adenosine	Tanaka *et al*., 2004[23]
Pseudomonas aeruginosa	Bacteria, Proteobacteria	6F3M<br>6F3N<br>6F3O<br>6F3P<br>6F3Q	Adenosine, zinc<br>SAH/adenosine, zinc<br>Adenine, zinc<br>3´-deoxyadenosine<br>Adenine, rubidium	Czyrko *et al*., 2018[7]
Rattus norvegicus	Eukaryota, Animalia	1B3R	None	Hu *et al*., 1999[24]
		1K0U	Eritadenine	Huang *et al*., 2002[25]
		1KY4	None	Takata *et al*.,2002[26]
		2H5L	3-deazaeritadenine	Yamada *et al*., 2005[27]
Synechocystis sp.	Bacteria, Cyanobacteria	7O5L<br>7O5M	Adenosine, rubidium<br>Adenosine	Malecki *et al*.,2022[28]
Trypanosoma brucei	Eukaryota, Euglenozoa	3H9U[29]	Adenosine	unpublished
Thermotoga maritima	Bacteria, Thermotogae	3X2E<br>3X2F	None (open)<br>None (closed)	Zheng *et al*., 2015[30]
		5TOV<br>5TOW	NADH<br>NADH, adenosine	Brzezinski *et al*., 2017[31]

Table S5. Interactions between the active site of SAHHs and the bound substrate.

Complex Type	Base moiety Interactions	Other Hydrogen Bonds	Non-bonded Contacts
SacSAHH•NAD•adenosine	Adenine:<br>N1 - Thr53<br>N6 - Glu55, His344<br>N7 - His344	O2´ - Glu147, Asp181<br>O3´ - Lys177<br>O5´ - His51, Asp124	Thr56, Thr148, His292, Leu338, Gly343, Met349, Phe353, NAD501
MmaSAHH•NAD•inosine	Hypoxanthine:<br>N1 - Glu72<br>O6 - Lys74<br>N7 - His364	O2´ - Lys198<br>O3´ - Lys198<br>O4´ - His70<br>O5´ - His70, His313	Thr75, Asp143, Glu168, Leu355, Leu358, Gly363, Met369, Phe373, NAD502
PfuSAHH•NAD•inosine	Hypoxanthine:<br>N1 - Glu57<br>O6 - Lys59<br>N7 - His350	O2´ - Glu153, Lys183, Asp187<br>O3´ - Thr154, Lys183<br>O5´ - His55, Asp128, His298	Thr60, Leu341, Leu344, Gly349, Met355, Phe359, NAD601
PfuSAHH•NAD•SIH	Hypoxanthine:<br>N1 - Glu57<br>O6 - Lys59<br>N7 - His350	O2´ - Glu153, Asp187<br>O3´ - Thr154, Lys183:<br>SD - His55<br>N - Asp128, Ser79<br>O - Asn80<br>OXT - Phe299	Thr60, Gly297, His298, Leu341, Leu344, Gly349, Met355, Phe359, NAD502
MmSAHH•NAD•inosine	Hypoxanthine:<br>N1 - Thr57<br>N7 - His353	O3´ - Thr157, Lys186<br>O4' - His55<br>O5´ - His55, His301	Leu54, Glu59, Thr60, Asp131, Glu156, Asp190, Leu347, Gly352, Met358, Phe362, NAD601

Bioinformatical results

Table S6. Homologues of *Mj*DadD found in the genomes whose SAHH was characterised in this work, using BLASTP[32]. Sequence identity and similarity were calculated using the Emboss Needle algorithm[33].

Organism	UniProt accession number	Sequence identity/similarity [%] to *Mj*DadD
Corynebacterium glutamicum	Not available	Not available
Lupinus luteus	Not available	Not available
Methanocaldococcus jannaschii	Q58936 (DADD_METJA)	100.0/100.0
Methanococcus maripaludis	Q6LX61 (DADD_METMP)	69.2/83.4
Mus musculus	Not available	Not available
Methanocella conradii	H8I882 (H8I882_METCZ)	35.3/54.4
Methanohalobium evestigatum	D7E8N2 (D7E8N2_METEZ)	44.6/65.1
Methanohalophilus halophilus	A0A1L3PZX9_9EURY	42.2/62.9
Methanocaldococcus infernus	D5VT65 (D5VT65_METIM)	81.7/90.2
Methanothrix thermoacetophila	A0B7V2 (DADD_METTP)	43.3/64.2
Pseudomonas aeruginosa	Not available	Not available
Pyrococcus furiosus	Q8U0P7 (MTAD_PYRFU)	46.0/65.0
Sulfolobus acidocaldarius	Not available	Not available
Saccharolobus solfataricus	Not available	Not available
Streptomyces albus	Not available	Not available
Streptomyces flocculus	Not available	Not available
Thermococcus kodakarensis	Q5JER0 (MTAD_THEKO)	51.7/67.4
Thermotoga maritima	Q9X034 (MTAD_THEMA)	39.1/57.9

Table S7 Homologues of *Ec*MTAN found in the genomes whose SAHH/SIHH was characterised in this work, using BLASTP[32]. Sequence identity and similarity were calculated using the Emboss Needle algorithm[33].

Organism	UniProt accession number	Sequence identity/similarity [%] to *Ec*MTAN
Corynebacterium glutamicum	A0A1R4F352_CORGT	21.9/36.8
Lupinus luteus	B6DX57_LUPLU	23.0/40.9
Methanocaldococcus jannaschii	Not available	Not available
Methanococcus maripaludis	Not available	Not available
Mus musculus	Not available	Not available
Methanocella conradii	Not available	Not available
Methanohalobium evestigatum	Not available	Not available
Methanohalophilus halophilus	Not available	Not available
Methanocaldococcus infernus	Not available	Not available
Methanothrix thermoacetophila	Not available	Not available
Pseudomonas aeruginosa	Not available	Not available
Pyrococcus furiosus	Not available	Not available
Sulfolobus acidocaldarius	Not available	Not available
Saccharolobus solfataricus	Not available	Not available
Streptomyces albus	Not available	Not available
Streptomyces flocculus	Not available	Not available
Thermococcus kodakarensis	Not available	Not available
Thermotoga maritima	MTNN_THEMA	27.2/46.6

SIH enzymatic synthesis and structure verification

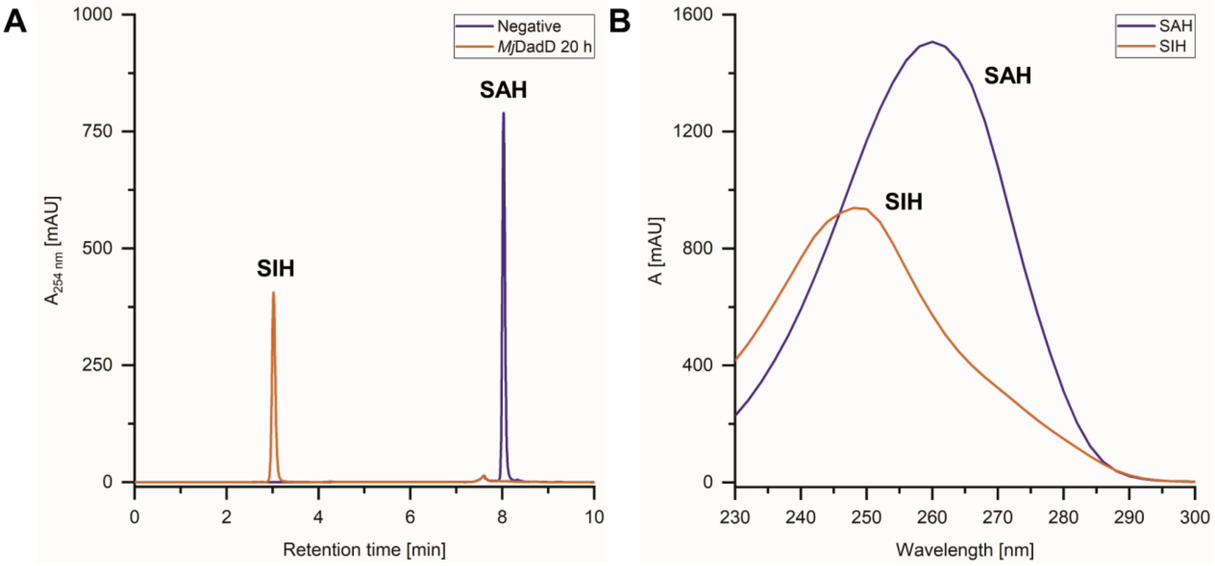

Figure S1. A. HPLC chromatogram showing the conversion of SAH (purple; 8.0 min) to SIH (orange; 3.0 min) catalysed by *Mj*DadD. **B.** UV spectrum of SAH and SIH showing the absorbance maximum shift between SAH (purple; 260 nm) and SIH (orange; 248 nm).

DMSO-d6 was initially used to solve the compound and the solvent signals are still visible in the NMR spectra [^{13}C NMR: DMSO-d6 38.5 ppm (septet, CD_3)]; ultimately D_2O was used for dissolving the powder after lyophilisation. Signals for the enzyme storage buffer are also visible in the NMR spectra [^{1}H NMR of glycerol and Tris signals: δ = 3.24 (dd for 2 H, glycerol), 3.34 (dd for 2 H, glycerol), 3.44-3.51 (m, 1 H, C-H in glycerol, also overlapping with Tris signal) ^{13}C NMR: δ = 72.0 (C-H in glycerol), 62.4 (C-H_2 in glycerol), 60.6 (C-H_2 in Tris); smaller signal for quaternary C in Tris not visible at this concentration].

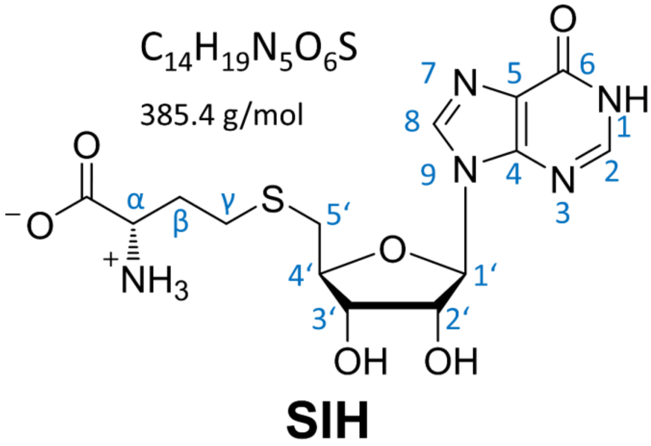

Figure S2A. Structure, molecular formula and molecular weight of SIH. Carbon atoms are numbered according to the assignment of the NMR signals.

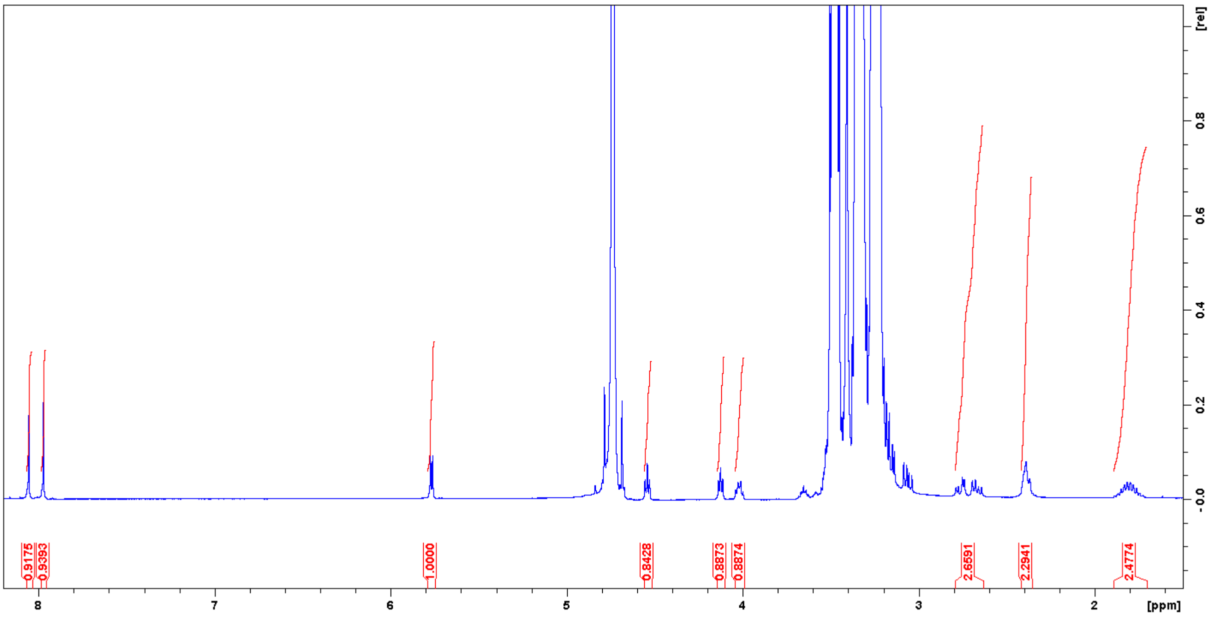

Figure S2B. ^{1}H NMR spectrum of enzymatically synthesised SIH.

^{1}H NMR (D_2O): δ = 8.06 (s, 1 H, H-8), 7.98 (s, 1 H, H-2), 5.77 (d, *J* = 5.06 Hz, 1 H, H-1'), 4.55 (t, J = 5.06 Hz, 1 H, H-2'), 4.13 (t, *J* = 5.06 Hz, 1 H, H-3'), 4.02 (dt, J = 4.87, 6.38 Hz, 1 H, H-4'), 3.52-3.55 (m, 1 H, H_{α}), 2.66 (dd, J = 6,98, 14.20 Hz, 1 H, H-5'$_{A}$), 2.76 (dd, J = 5.06, 14.20 Hz, 1 H, H-5'$_{B}$), 2.34-2.44 (m, 2 H, H_{γ}), 1.70-1.90 (m, 2 H, H_{β}).

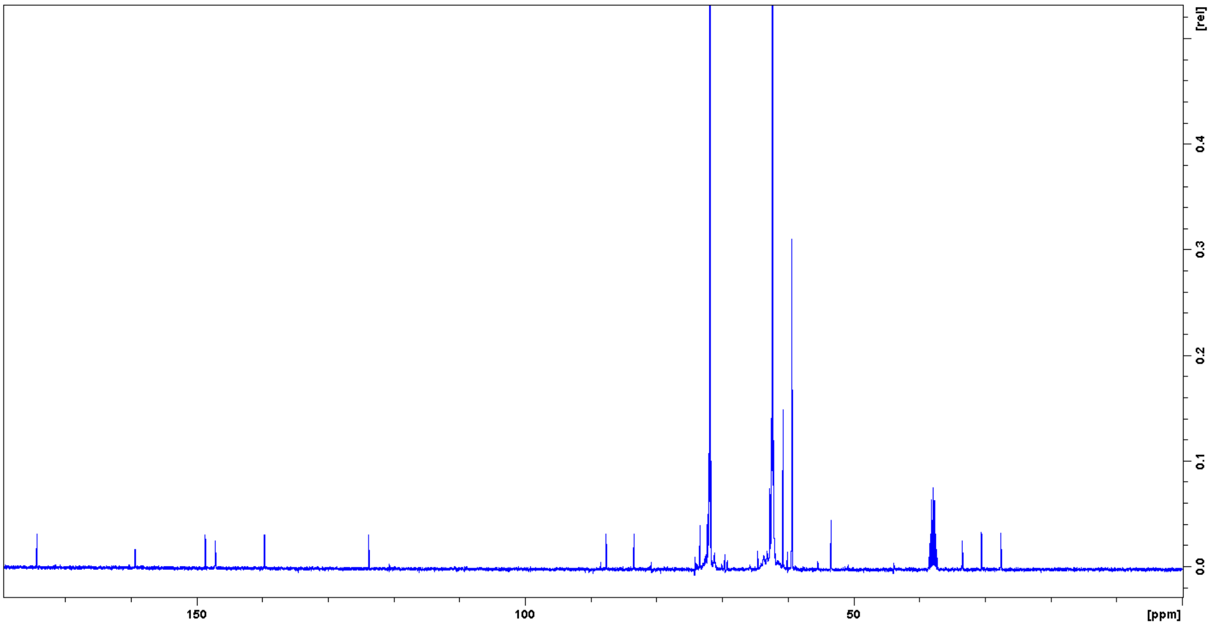

Figure S2C. ^{13}C NMR spectrum of enzymatically synthesised SIH.

^{13}C NMR (D_2O): δ = 174.4 (COOH), 159.2 (C-6), 148.7 (C-4), 147.1 (C-2), 139.7 (C-8), 123.8 (C-5), 87.6 (C-1), 83.4 (C-4'), 73.3 (C-2'), 72.3 (C-3'), 53.4 (C_{α}), 33.4 (C-5'), 30.4 (C_{β}), 27.6 (C_{γ}).

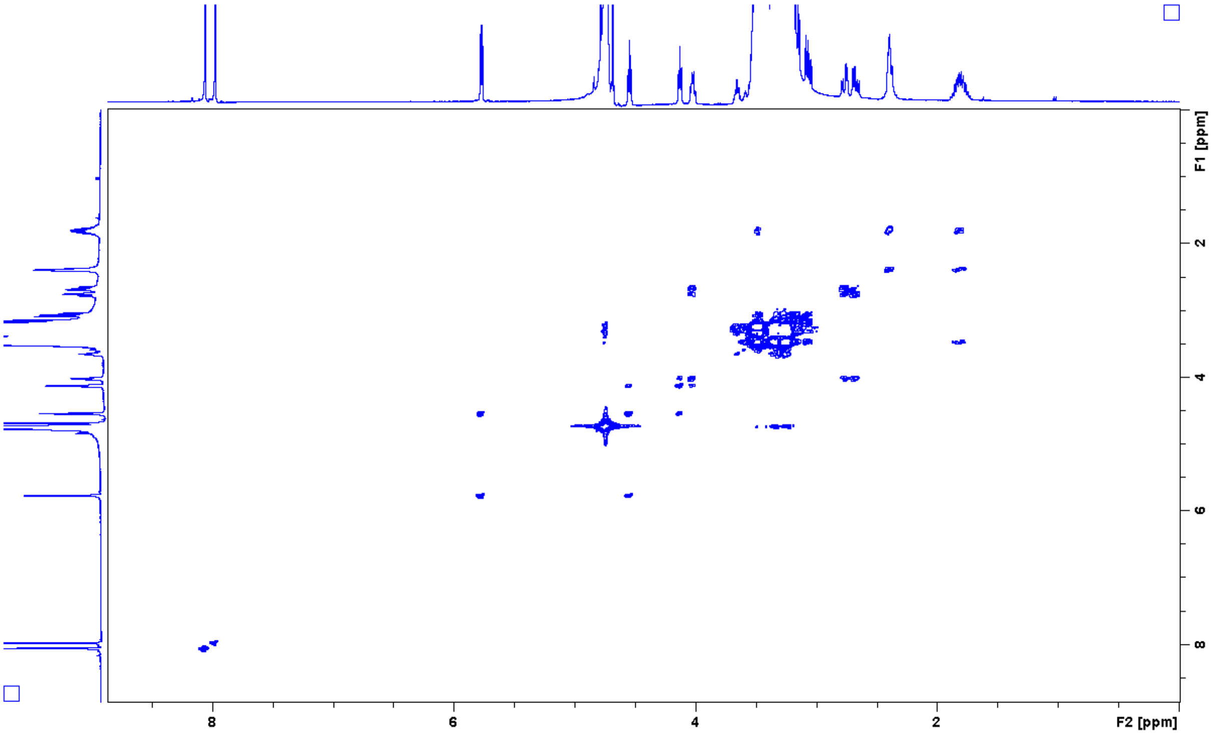

Figure S2D. H,H-COSY spectrum of enzymatically synthesised SIH.

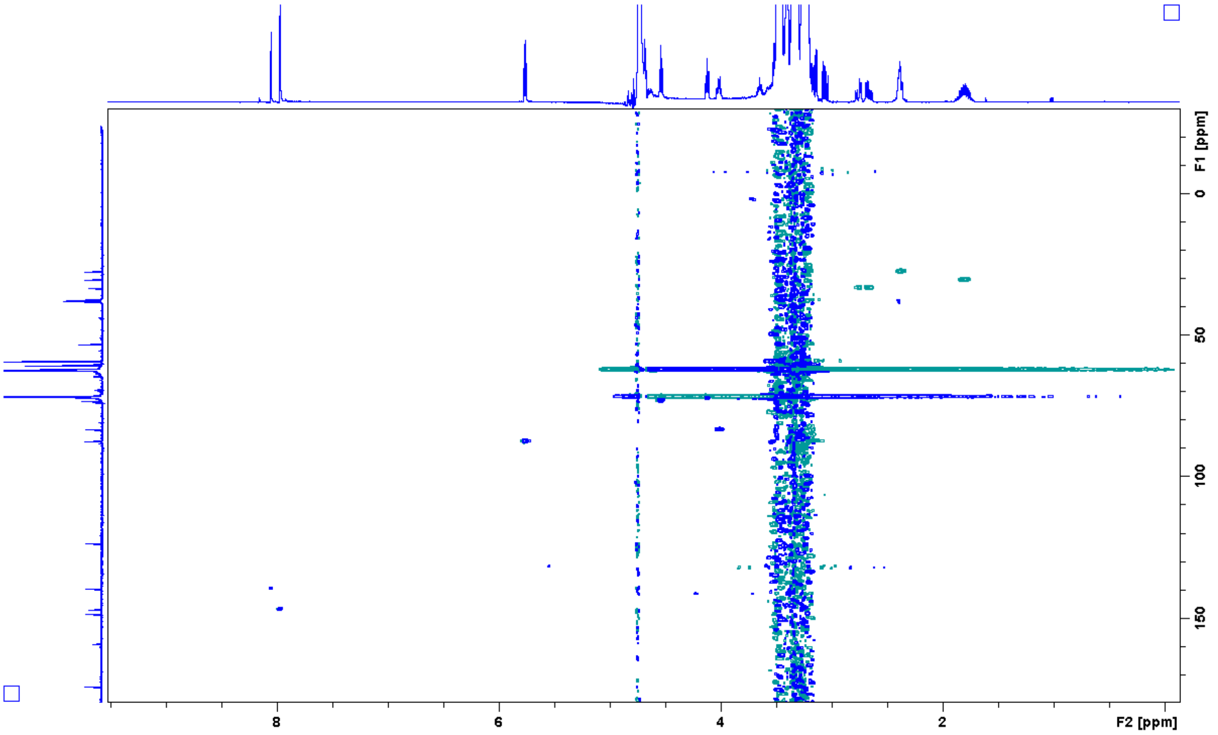

Figure S2E. HSQC spectrum of enzymatically synthesised SIH.

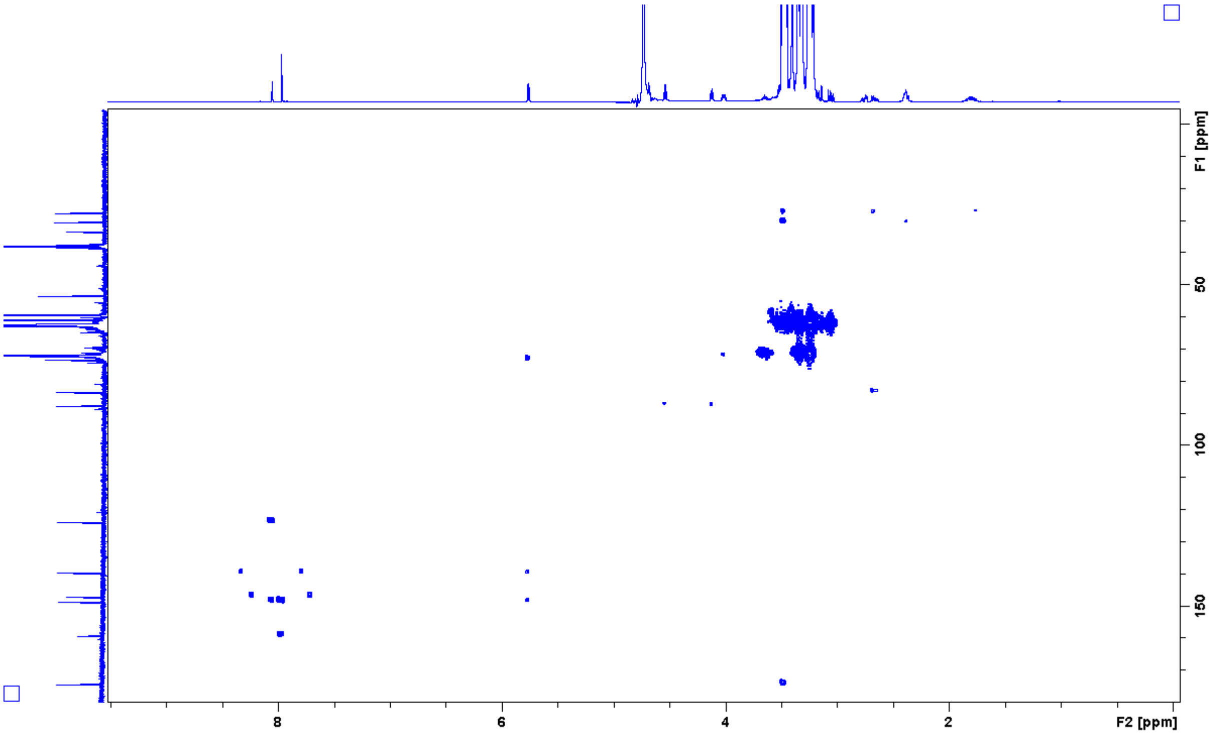

Figure S2F. HMBC spectrum of enzymatically synthesised SIH.

SAHH/SIHH characterisation

The protein standard featuring pink bands is the Precision Plus Protein Dual Color Standard by Bio-Rad Laboratories GmbH (Feldkirchen, Germany; taken from https://www.bio-rad.com/en-de/sku/1610374-precision-plus-protein-dual-color-standards-500-ul?ID=1610374), while the one with green and orange bands is the Color Prestained Protein Standard, Broad Range (11–245 kDa; taken from https://international.neb.com/products/p7712-color-prestained-protein-standard-broad-range-11-245-kda) manufactured by New England Biolabs GmbH (Frankfurt am Main, Germany). The standard with only blue bands is the Blue Prestained Protein Standard, Broad Range (11–190 kDa; New England Biolabs GmbH, Frankfurt am Main, Germany taken from https://international.neb.com/products/p7706-blue-prestained-protein-standard-broad-range-11-190-kda).

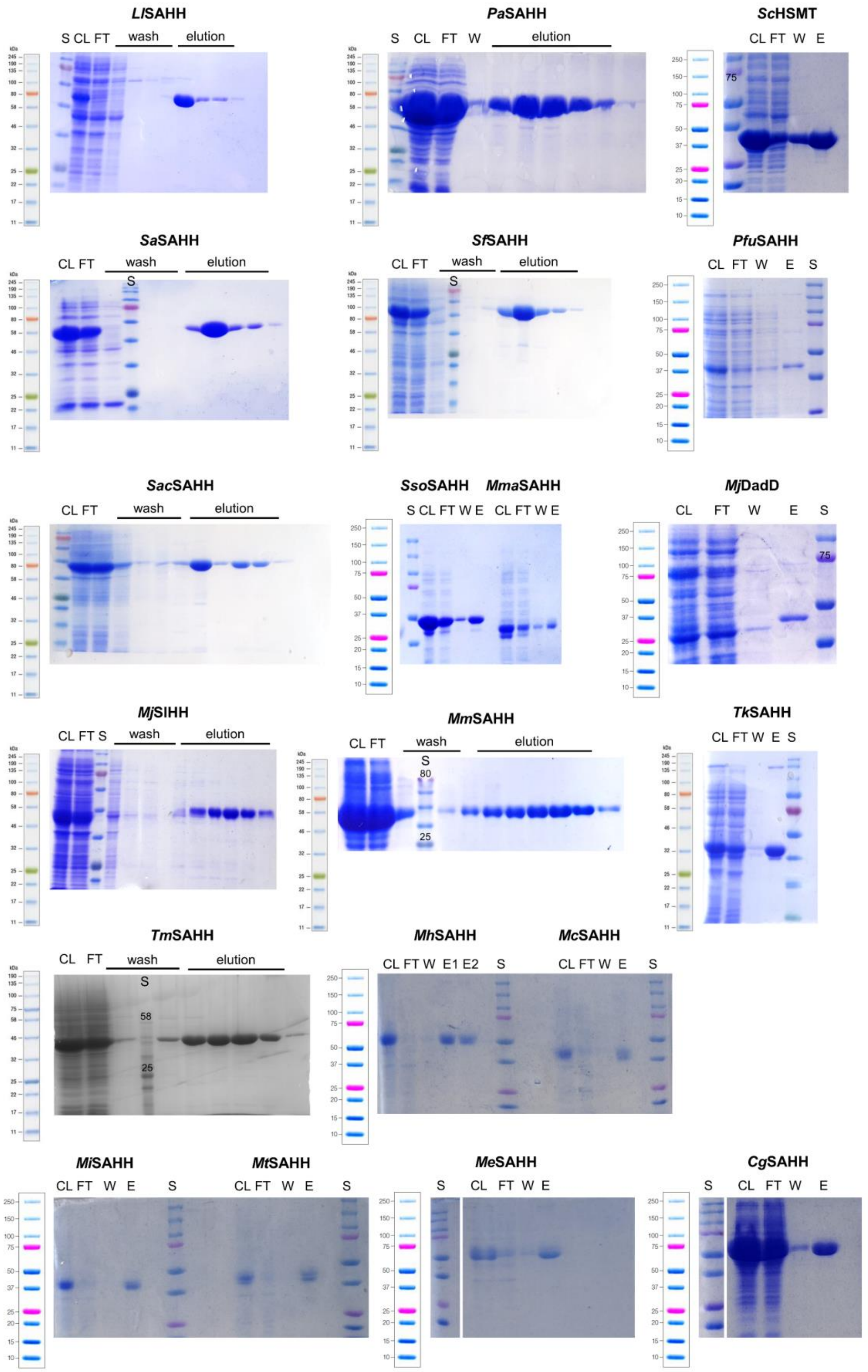

Figure S3. SDS gels of all purifications of enzymes used in this work[34] CL, crude lysate; E, elution; FT, flow through; S, standard protein ladder; W, wash. Gels were cropped if other samples were not relevant.

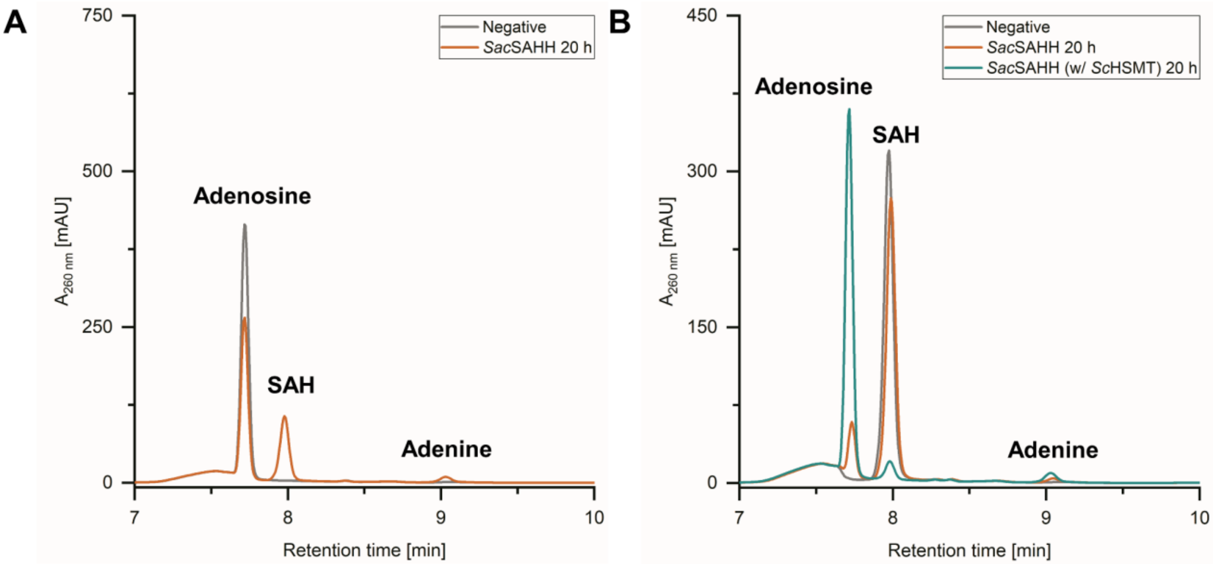

Figure S4A. HPLC chromatograms showing **A.** the SAH synthesis reaction and **B.** the SAH cleavage reaction (with and without the addition of *Sc*HSMT) catalysed by *Sac*SAHH.

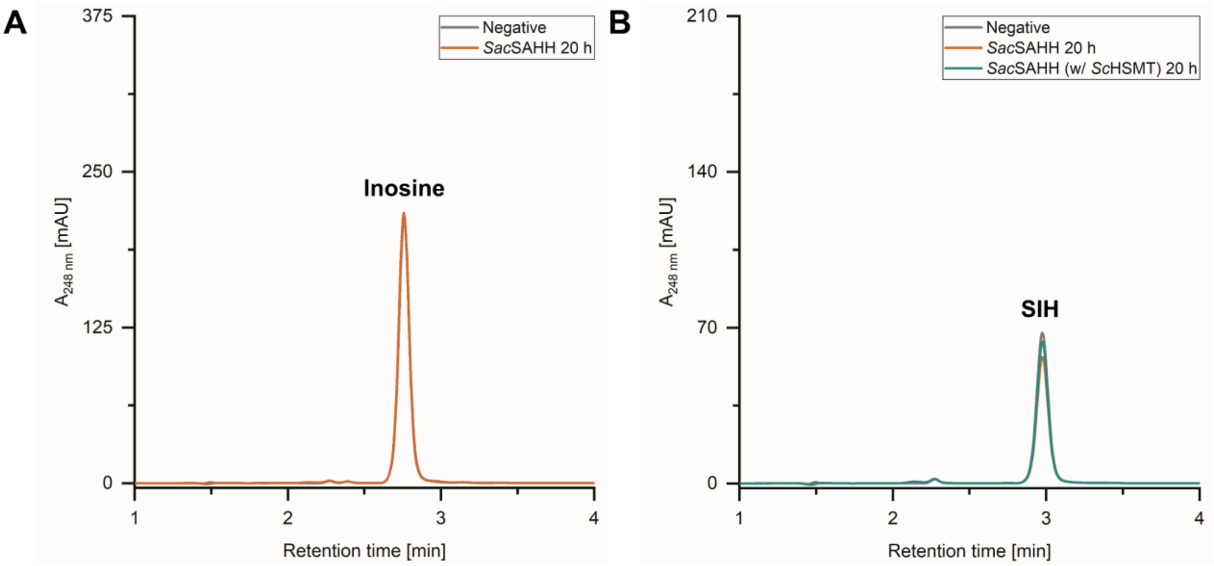

Figure S4B. HPLC chromatograms showing **A.** the SIH synthesis reaction and **B.** the SIH cleavage reaction (with and without the addition of *Sc*HSMT) catalysed by *Sac*SAHH.

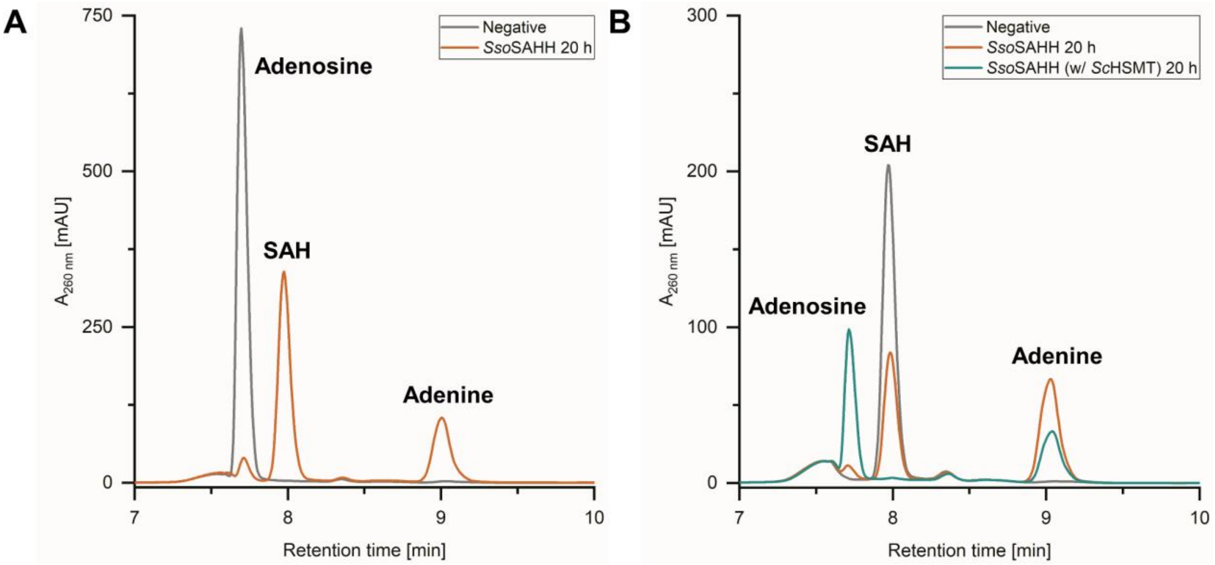

Figure S5A. HPLC chromatograms showing **A.** the SAH synthesis reaction and **B.** the SAH cleavage reaction (with and without the addition of *Sc*HSMT) catalysed by *Sso*SAHH.

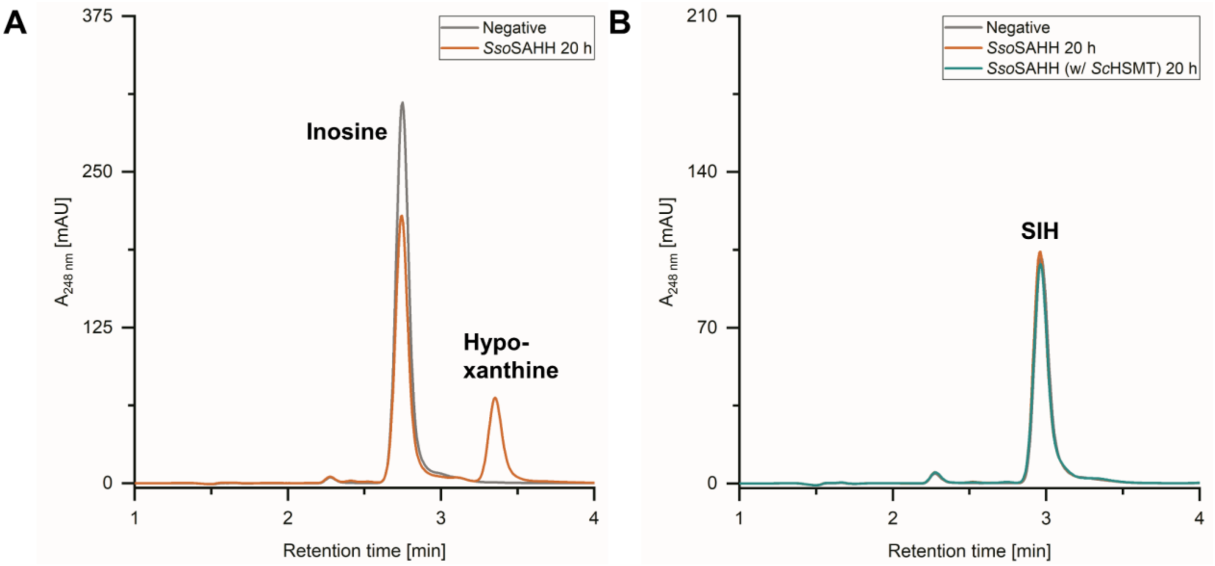

Figure S5B. HPLC chromatograms showing **A.** the SIH synthesis reaction and **B.** the SIH cleavage reaction (with and without the addition of *Sc*HSMT) catalysed by *Sso*SAHH.

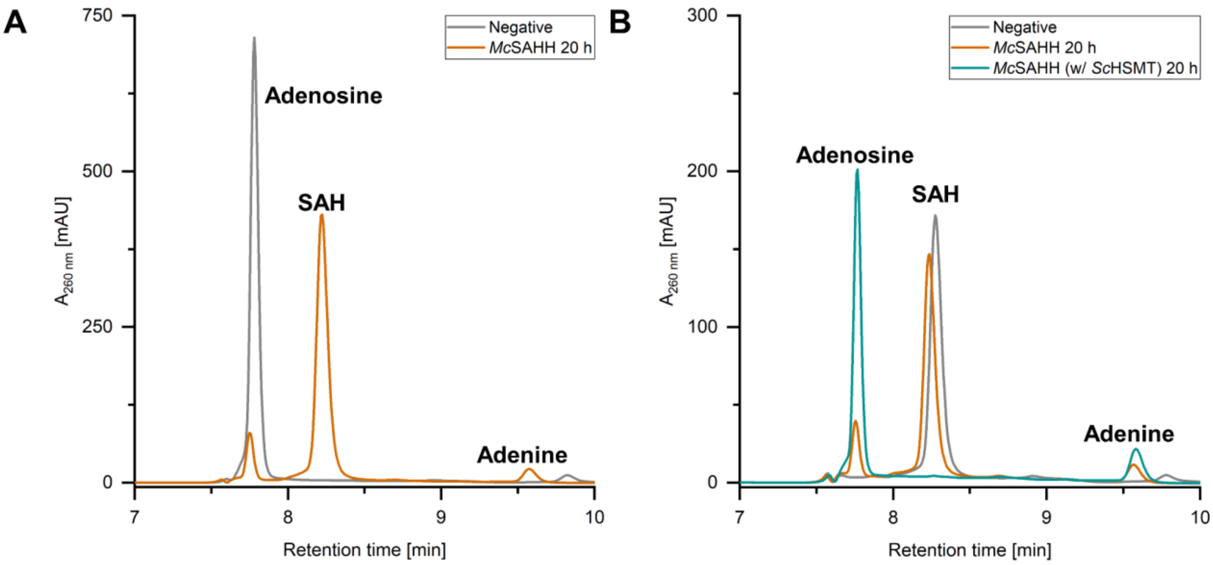

Figure S6A. HPLC chromatograms showing **A.** the SAH synthesis reaction and **B.** the SAH cleavage reaction (with and without the addition of *Sc*HSMT) catalysed by *Mc*SAHH.

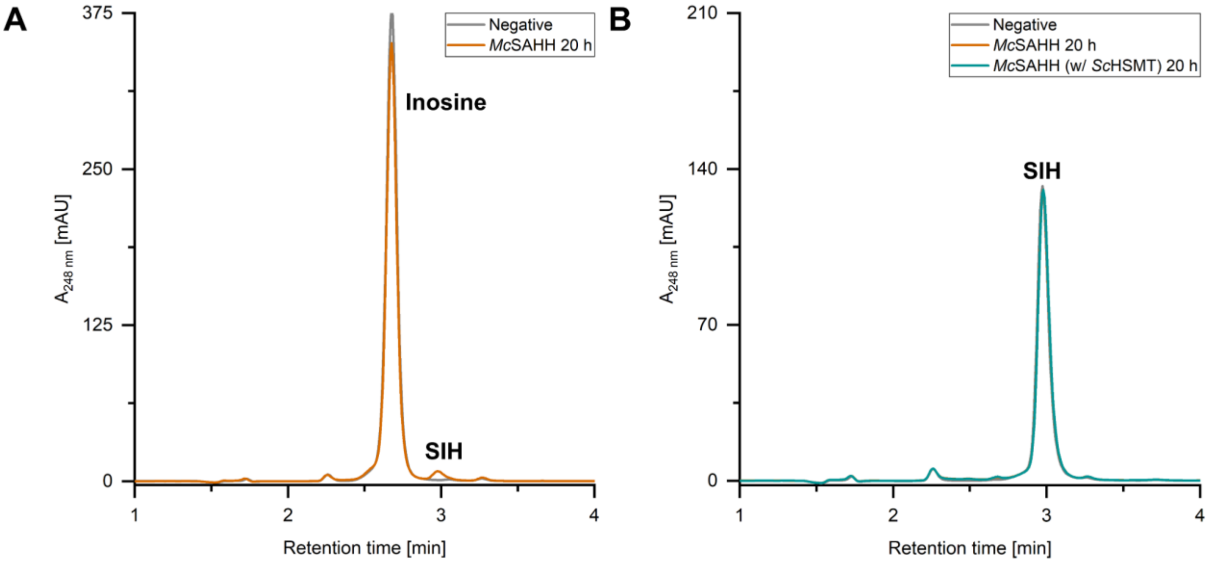

Figure S6B. HPLC chromatograms showing **A.** the SIH synthesis reaction and **B.** the SIH cleavage reaction (with and without the addition of *Sc*HSMT) catalysed by *Mc*SAHH.

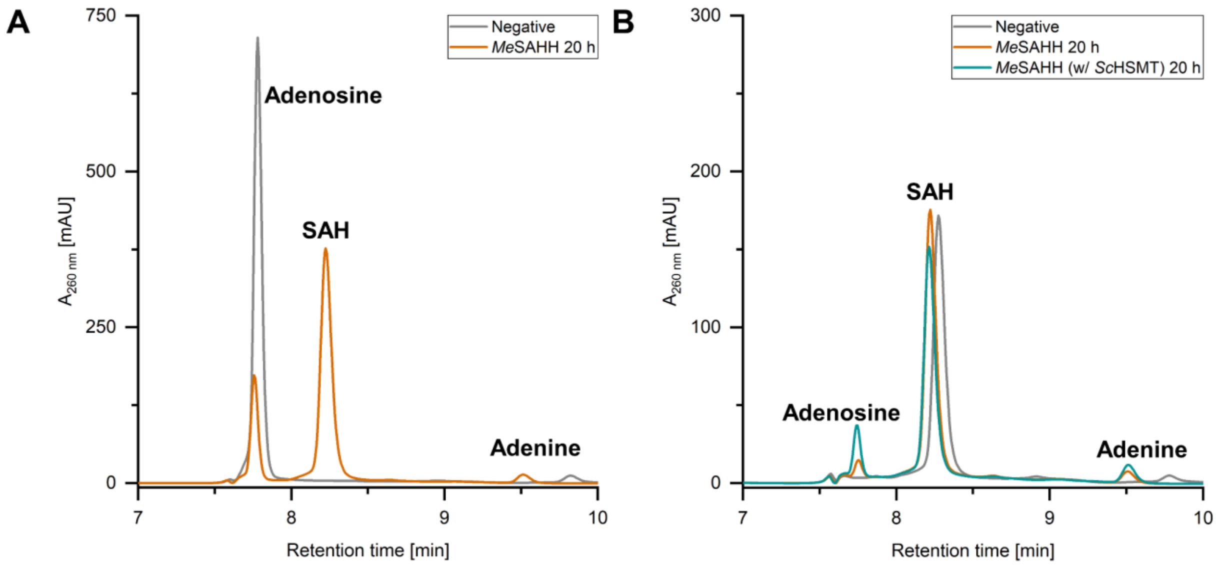

Figure S7A. HPLC chromatograms showing **A.** the SAH synthesis reaction and **B.** the SAH cleavage reaction (with and without the addition of *Sc*HSMT) catalysed by *Me*SAHH.

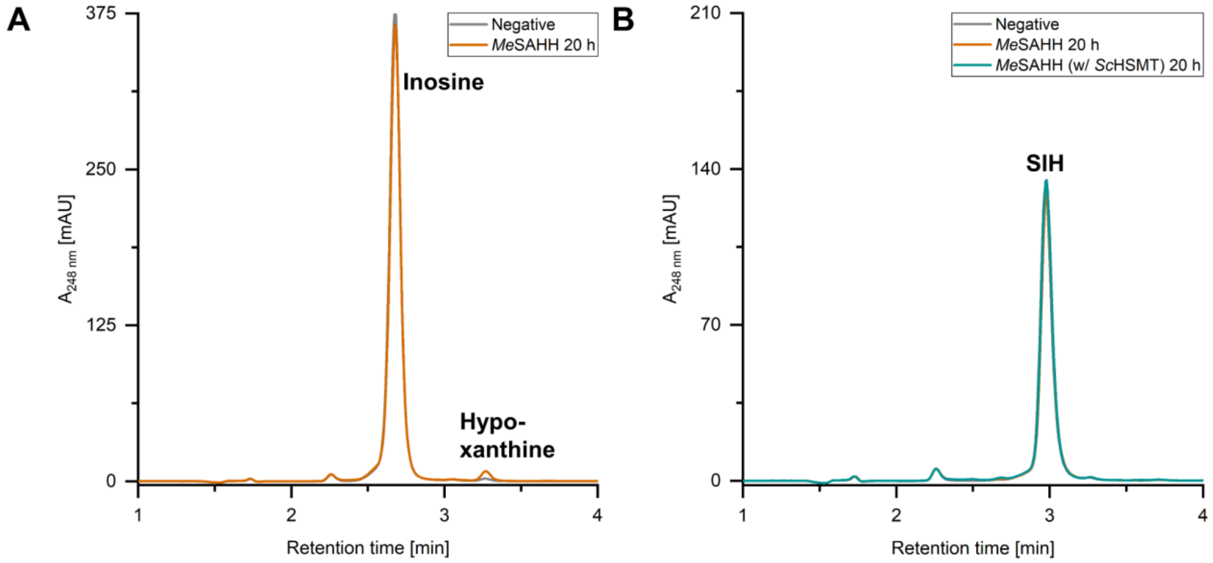

Figure S7B. HPLC chromatograms showing **A.** the SIH synthesis reaction and **B.** the SIH cleavage reaction (with and without the addition of *Sc*HSMT) catalysed by *Me*SAHH.

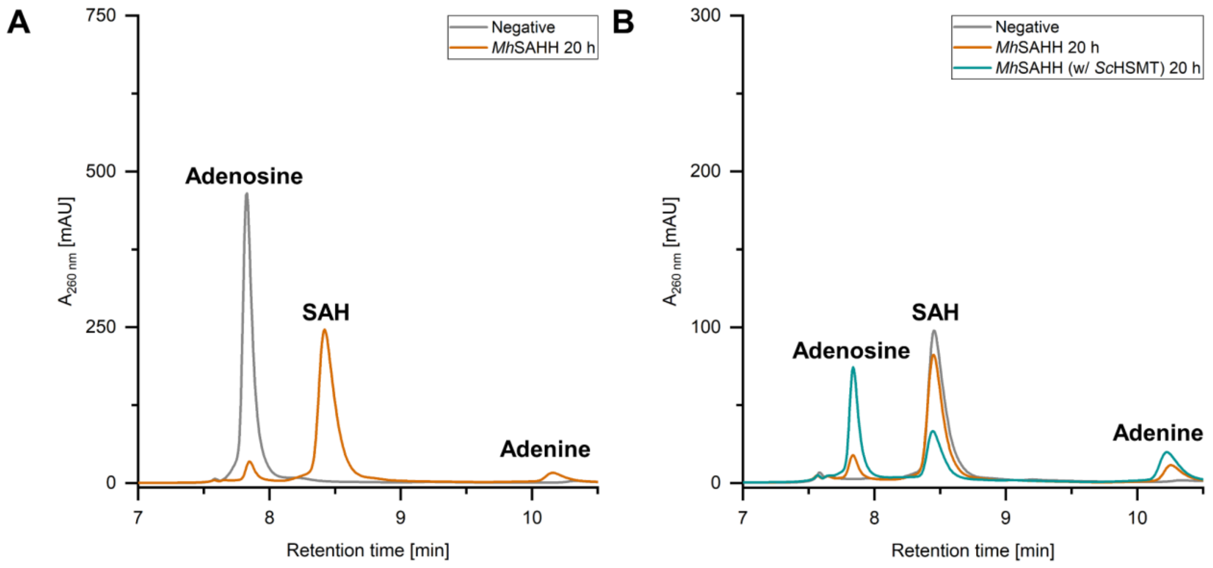

Figure S8A. HPLC chromatograms showing **A.** the SAH synthesis reaction and **B.** the SAH cleavage reaction (with and without the addition of *Sc*HSMT) catalysed by *Mh*SAHH.

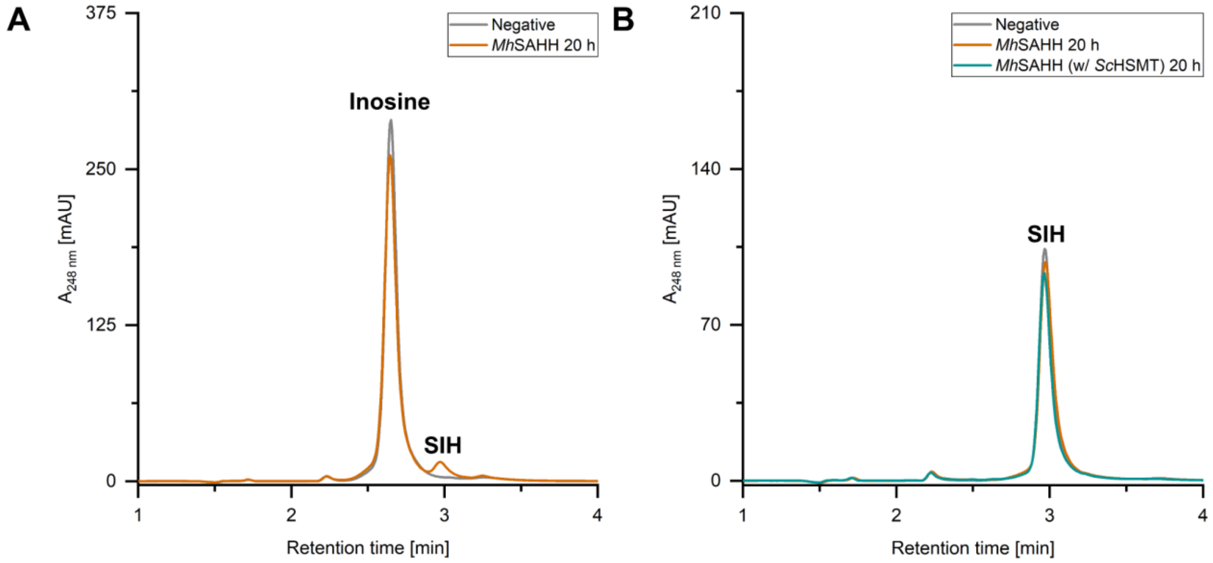

Figure S8B. HPLC chromatograms showing **A.** the SIH synthesis reaction and **B.** the SIH cleavage reaction (with and without the addition of *Sc*HSMT) catalysed by *Mh*SAHH.

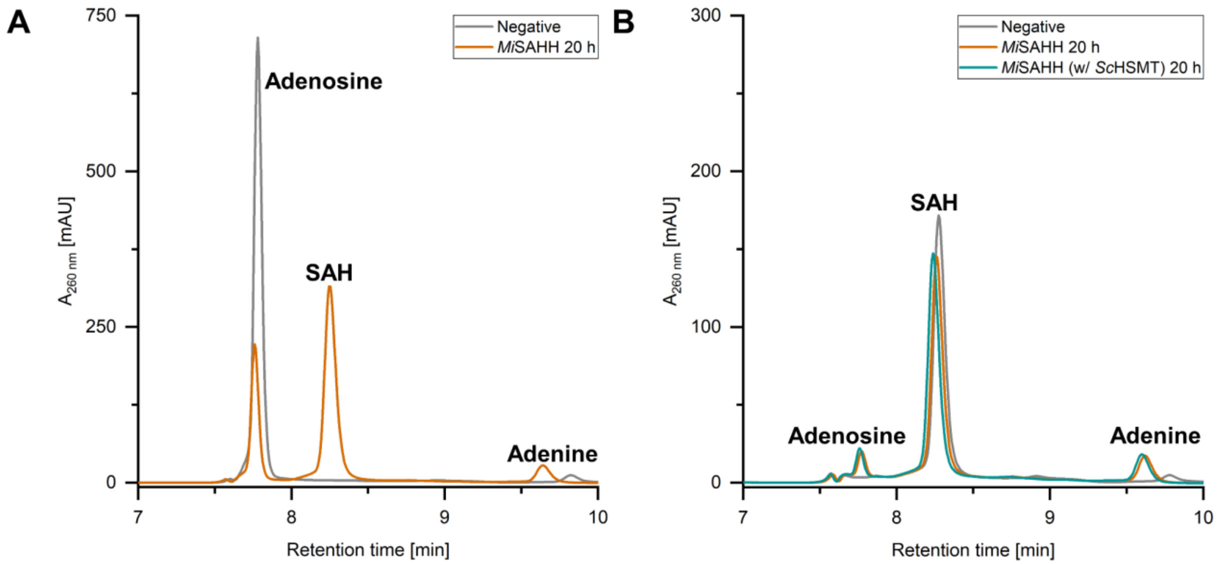

Figure S9A. HPLC chromatograms showing **A.** the SAH synthesis reaction and **B.** the SAH cleavage reaction (with and without the addition of *Sc*HSMT) catalysed by *Mi*SAHH.

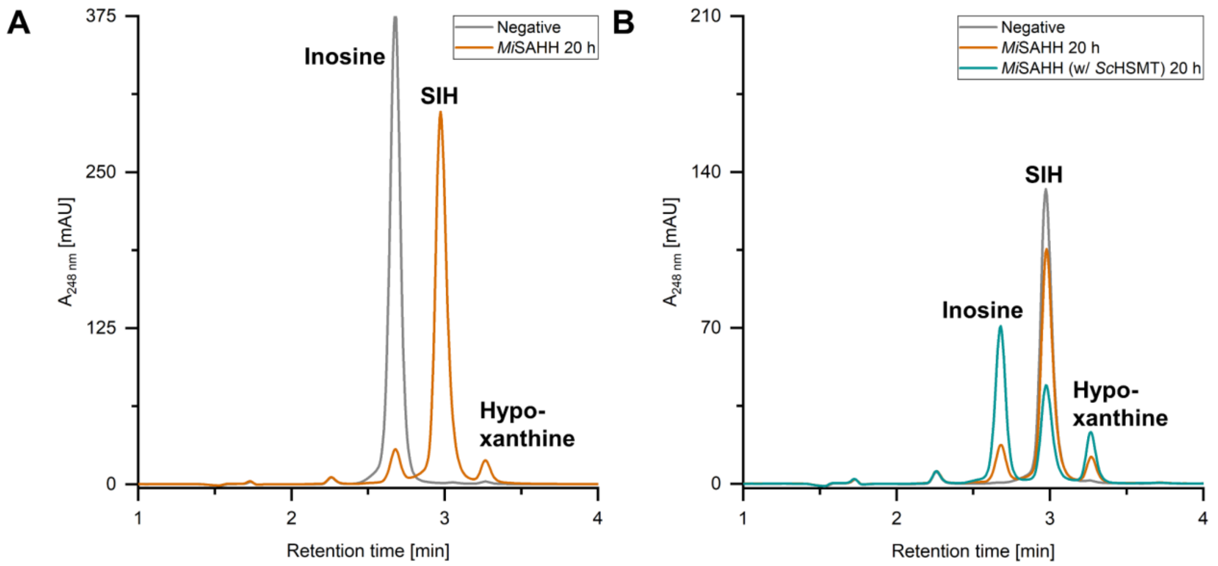

Figure S9B. HPLC chromatograms showing **A.** the SIH synthesis reaction and **B.** the SIH cleavage reaction (with and without the addition of *Sc*HSMT) catalysed by *Mi*SAHH.

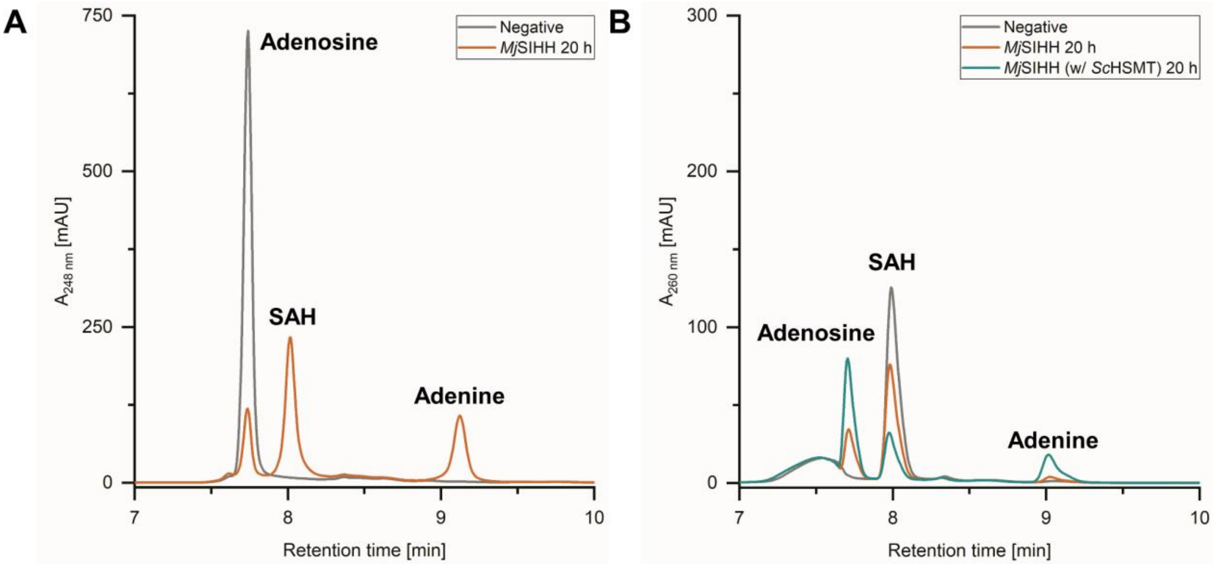

Figure S10A. HPLC chromatograms showing **A.** the SAH synthesis reaction and **B.** the SAH cleavage reaction (with and without the addition of *Sc*HSMT) catalysed by *Mj*SIHH.

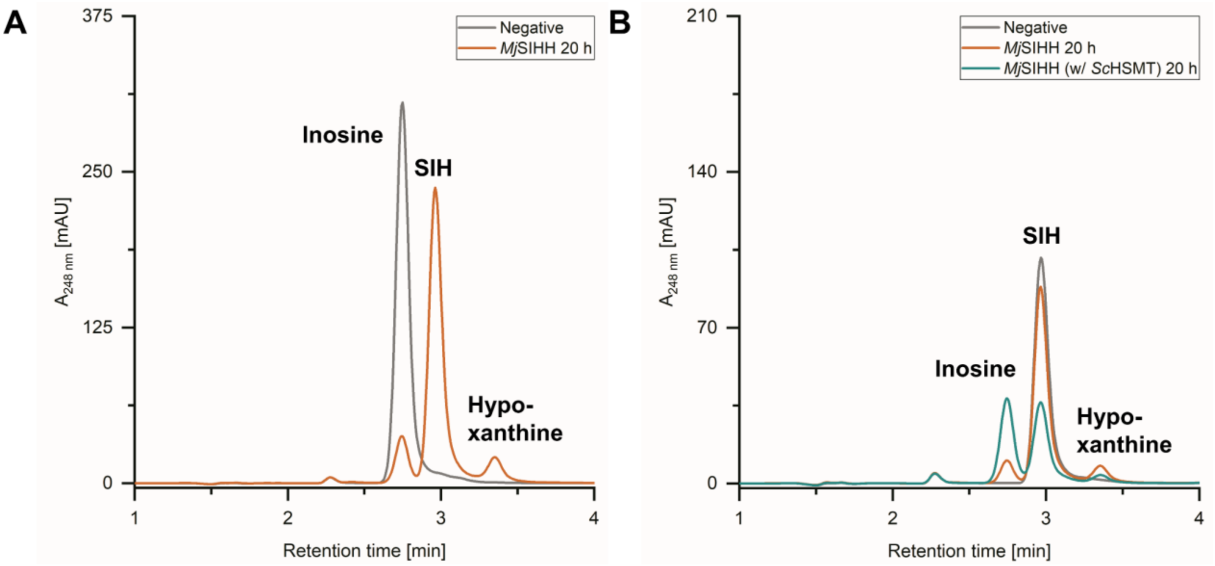

Figure S10B. HPLC chromatograms showing **A.** the SIH synthesis reaction and **B.** the SIH cleavage reaction (with and without the addition of *Sc*HSMT) catalysed by *Mj*SIHH.

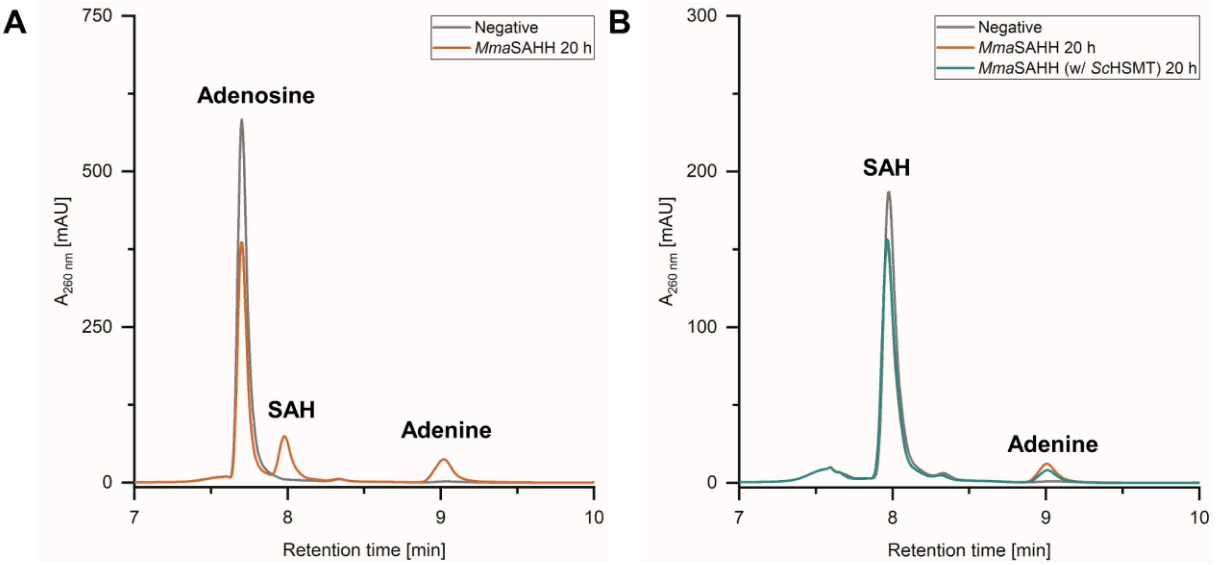

Figure S11A. HPLC chromatograms showing **A.** the SAH synthesis reaction and **B.** the SAH cleavage reaction (with and without the addition of *Sc*HSMT) catalysed by *Mma*SAHH.

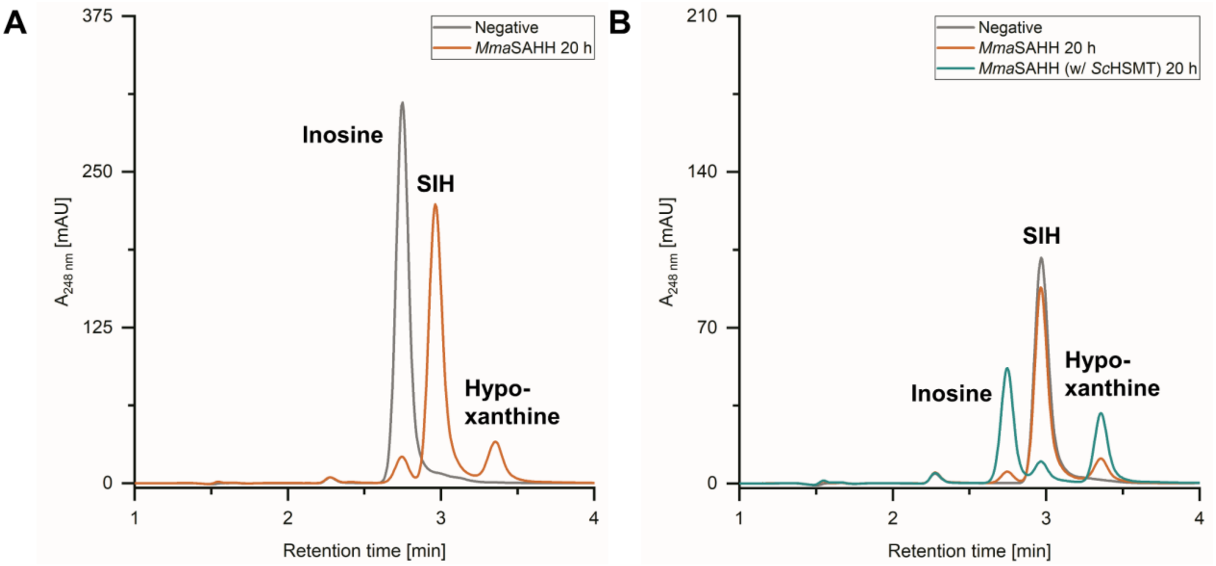

Figure S11B. HPLC chromatograms showing **A.** the SIH synthesis reaction and **B.** the SIH cleavage reaction (with and without the addition of *Sc*HSMT) catalysed by *Mma*SAHH.

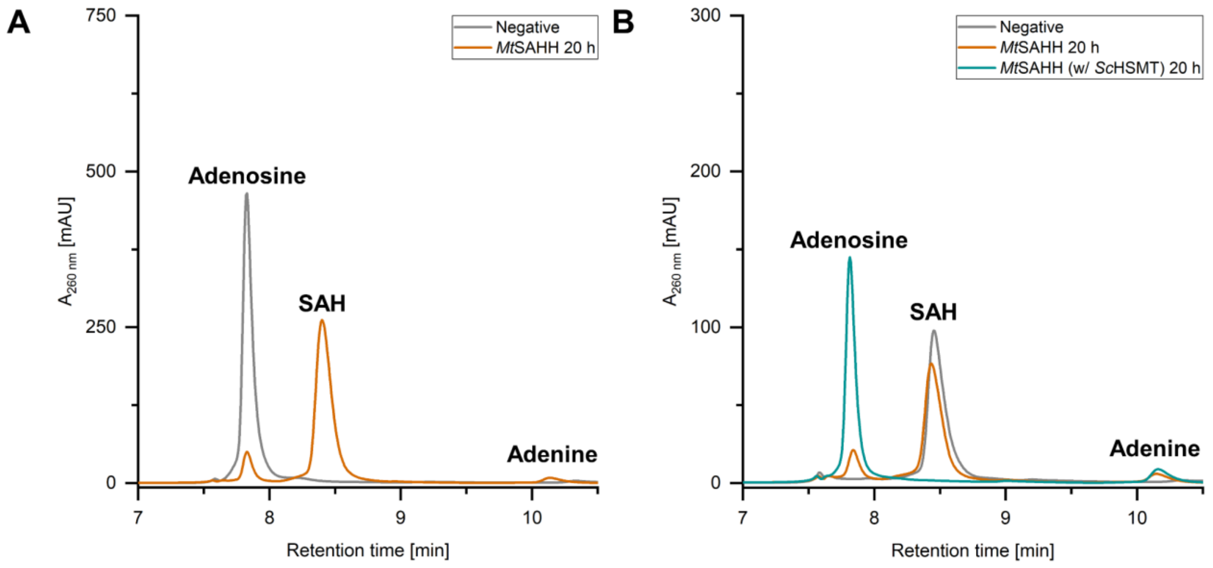

Figure S12A. HPLC chromatograms showing **A.** the SAH synthesis reaction and **B.** the SAH cleavage reaction (with and without the addition of *Sc*HSMT) catalysed by *Mt*SAHH.

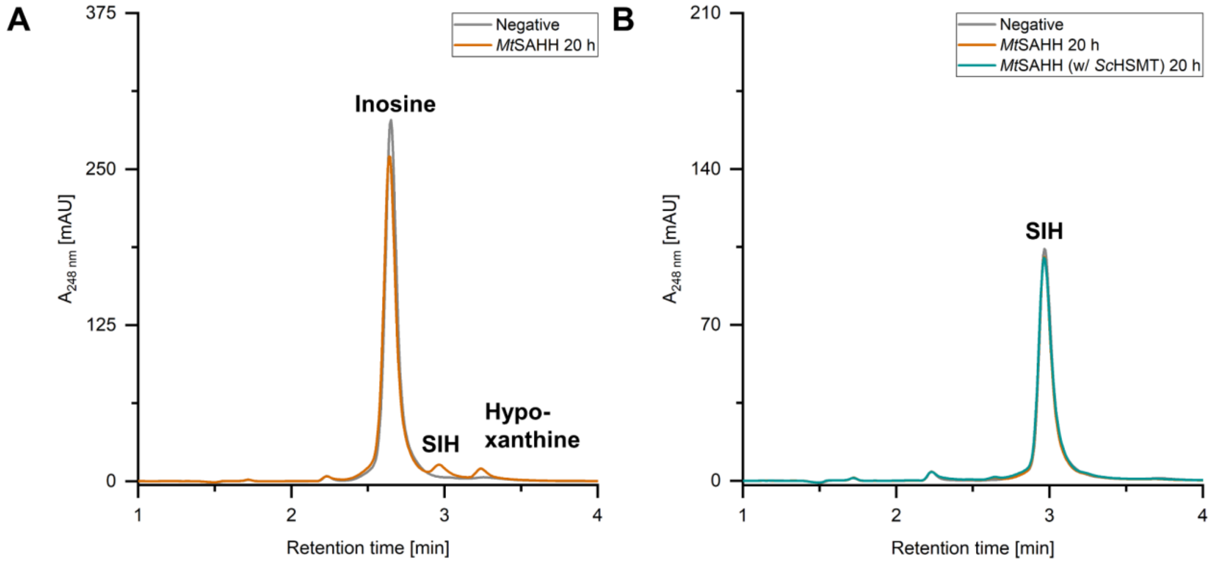

Figure S12B. HPLC chromatograms showing **A.** the SIH synthesis reaction and **B.** the SIH cleavage reaction (with and without the addition of *Sc*HSMT) catalysed by *Mt*SAHH.

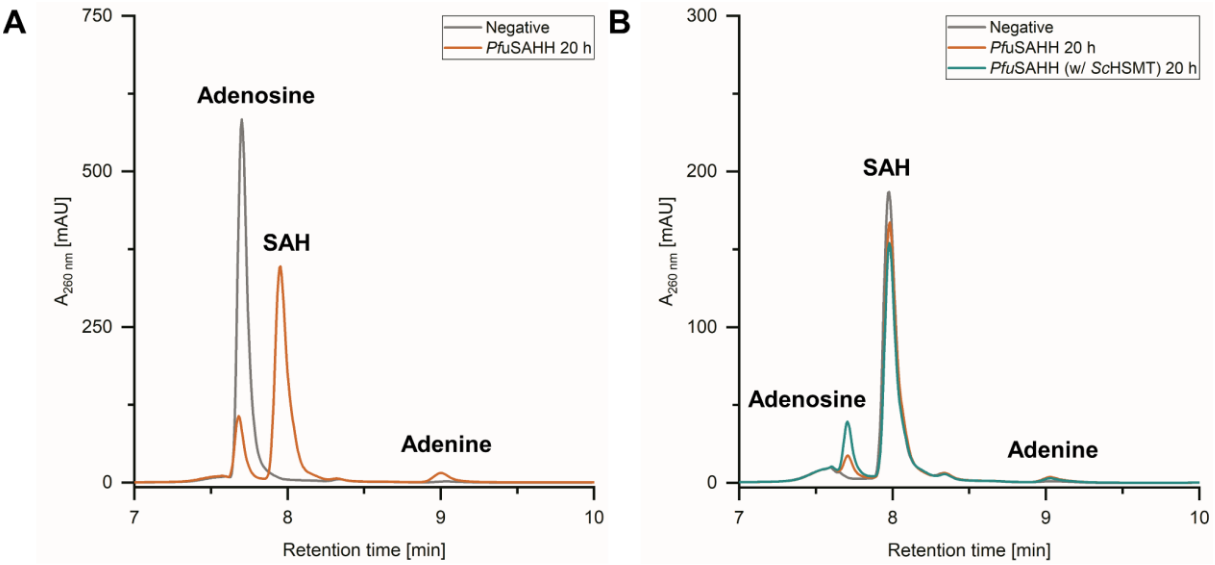

Figure S13A. HPLC chromatograms showing **A.** the SAH synthesis reaction and **B.** the SAH cleavage reaction (with and without the addition of *Sc*HSMT) catalysed by *Pfu*SAHH.

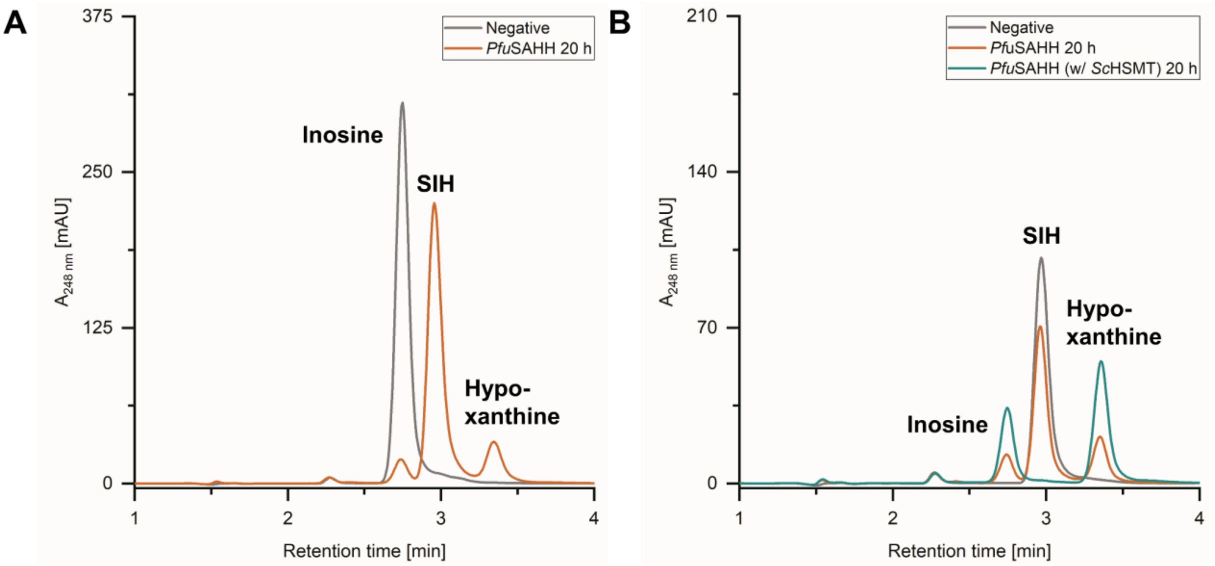

Figure S13B. HPLC chromatograms showing **A.** the SIH synthesis reaction and **B.** the SIH cleavage reaction (with and without the addition of *Sc*HSMT) catalysed by *Pfu*SAHH.

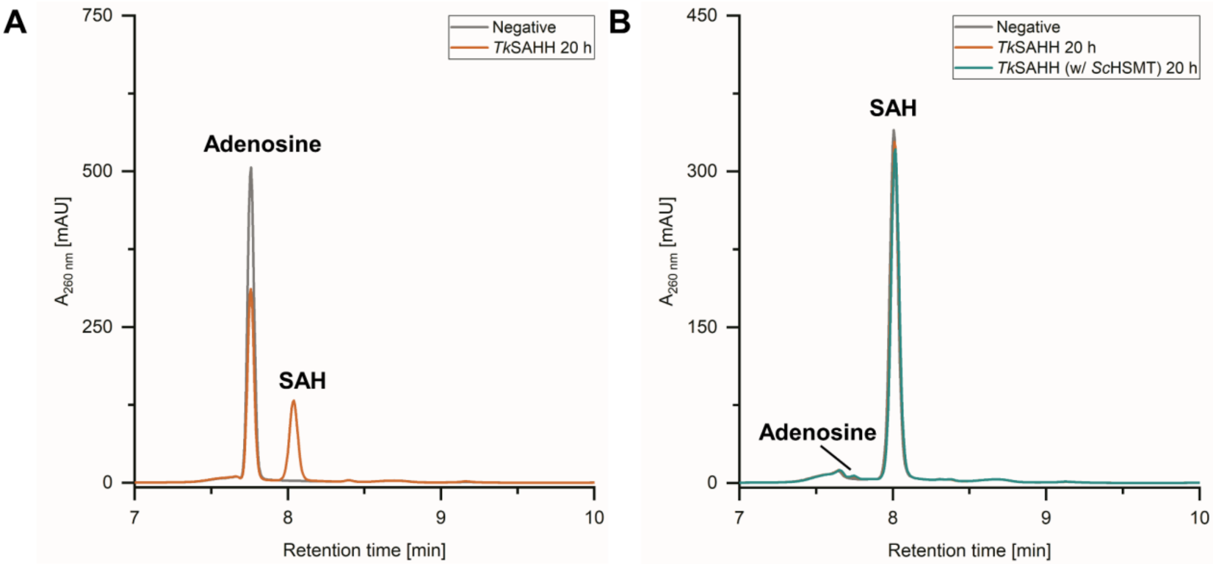

Figure S14A. HPLC chromatograms showing **A.** the SAH synthesis reaction and **B.** the SAH cleavage reaction (with and without the addition of *Sc*HSMT) catalysed by *Tk*SAHH.

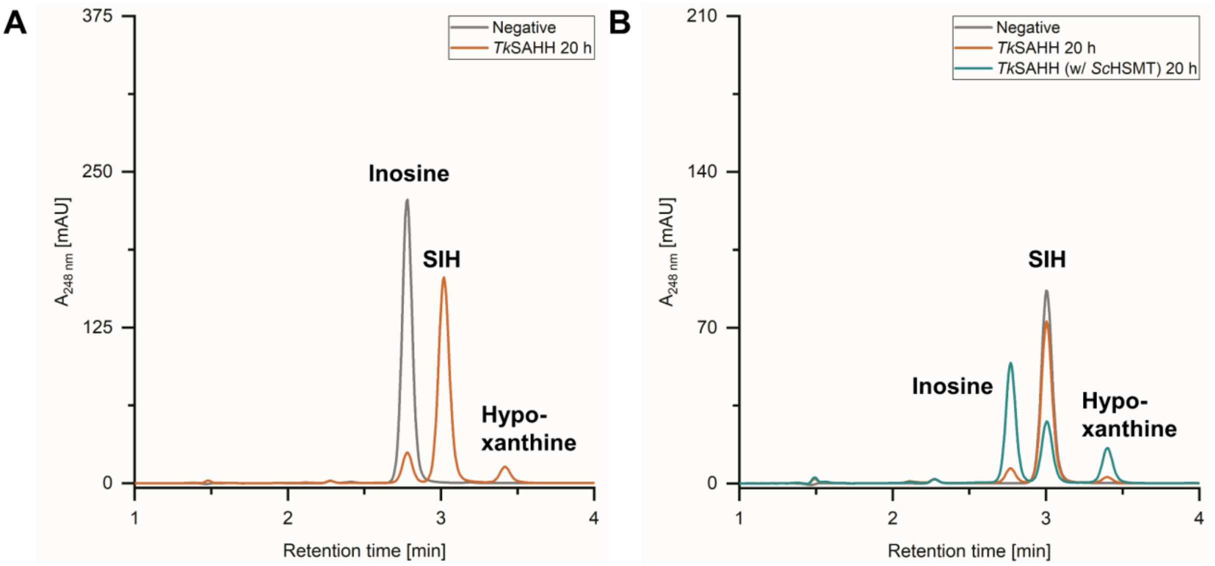

Figure S14B. HPLC chromatograms showing **A.** the SIH synthesis reaction and **B.** the SIH cleavage reaction (with and without the addition of *Sc*HSMT) catalysed by *Tk*SAHH.

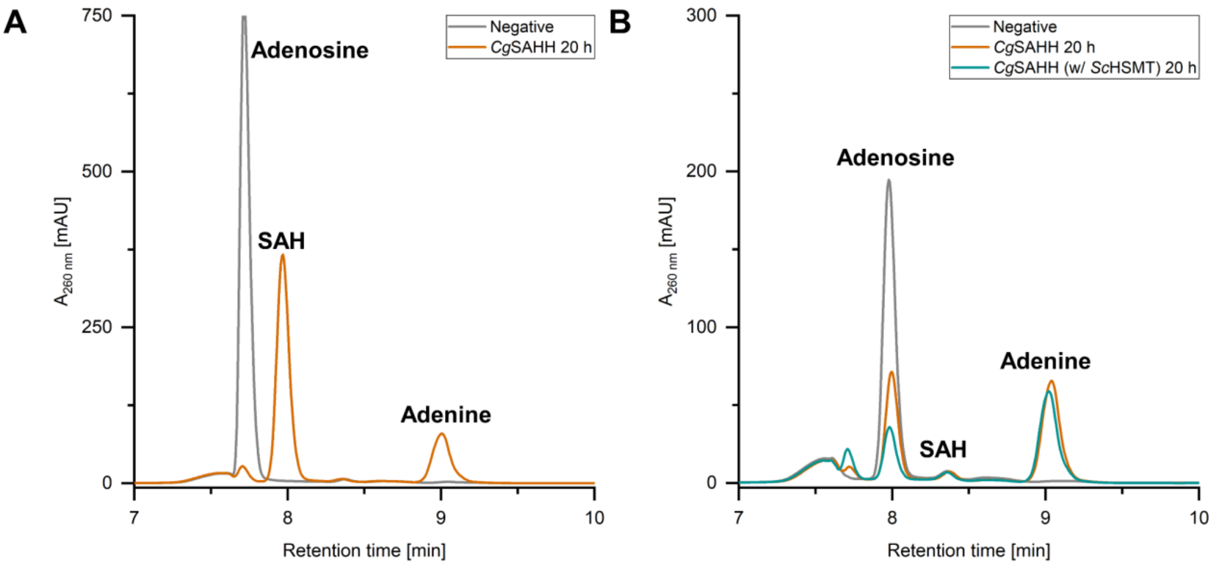

Figure S15A. HPLC chromatograms showing **A.** the SAH synthesis reaction and **B.** the SAH cleavage reaction (with and without the addition of *Sc*HSMT) catalysed by *Cg*SAHH.

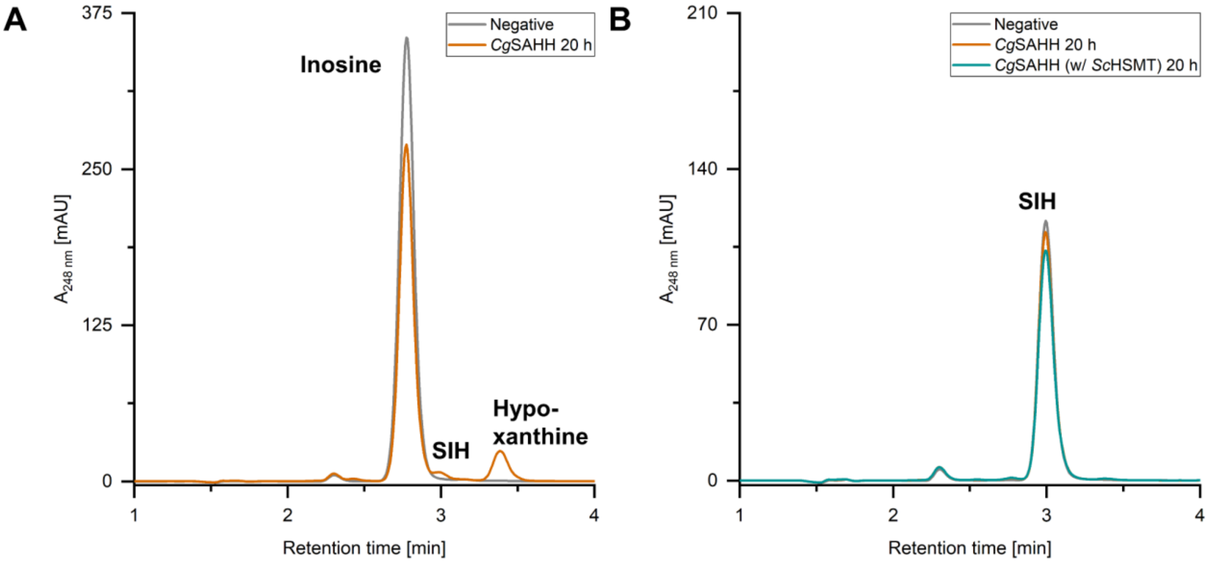

Figure S15B. HPLC chromatograms showing **A.** the SIH synthesis reaction and **B.** the SIH cleavage reaction (with and without the addition of *Sc*HSMT) catalysed by *Cg*SAHH.

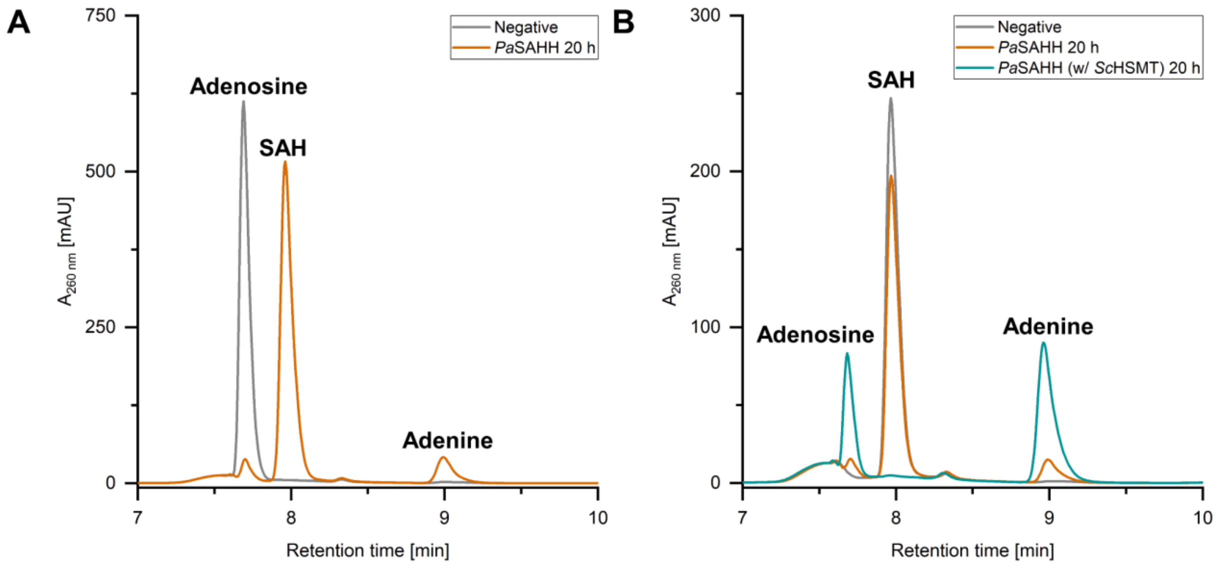

Figure S16A. HPLC chromatograms showing **A.** the SAH synthesis reaction and **B.** the SAH cleavage reaction (with and without the addition of *Sc*HSMT) catalysed by *Pa*SAHH.

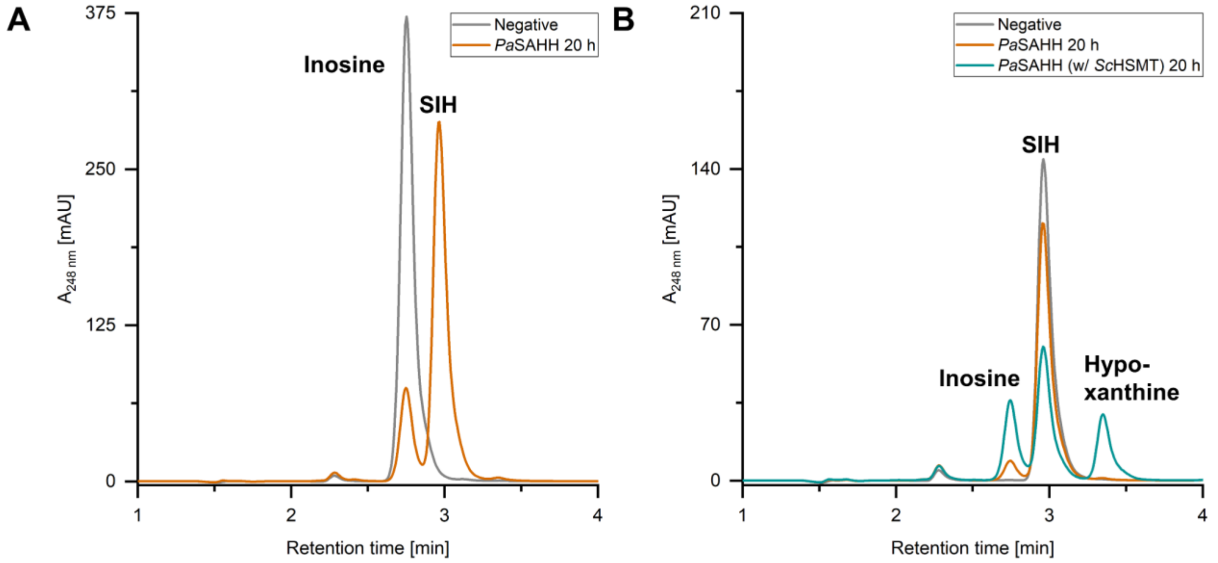

Figure S16B. HPLC chromatograms showing **A.** the SIH synthesis reaction and **B.** the SIH cleavage reaction (with and without the addition of *Sc*HSMT) catalysed by *Pa*SAHH.

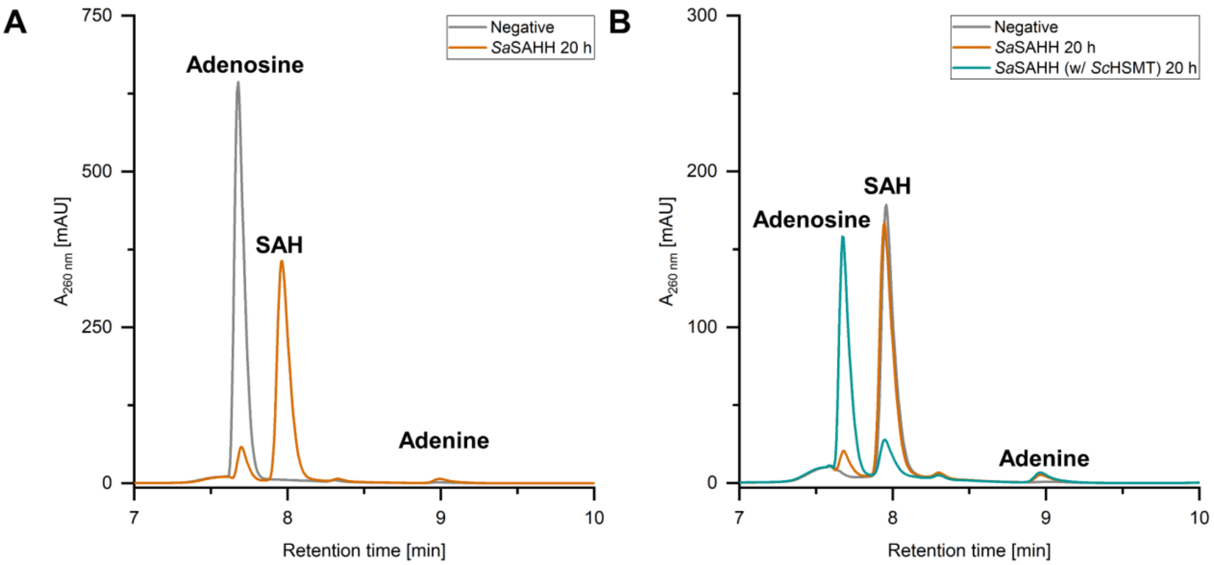

Figure S17A. HPLC chromatograms showing **A.** the SAH synthesis reaction and **B.** the SAH cleavage reaction (with and without the addition of *Sc*HSMT) catalysed by *Sa*SAHH.

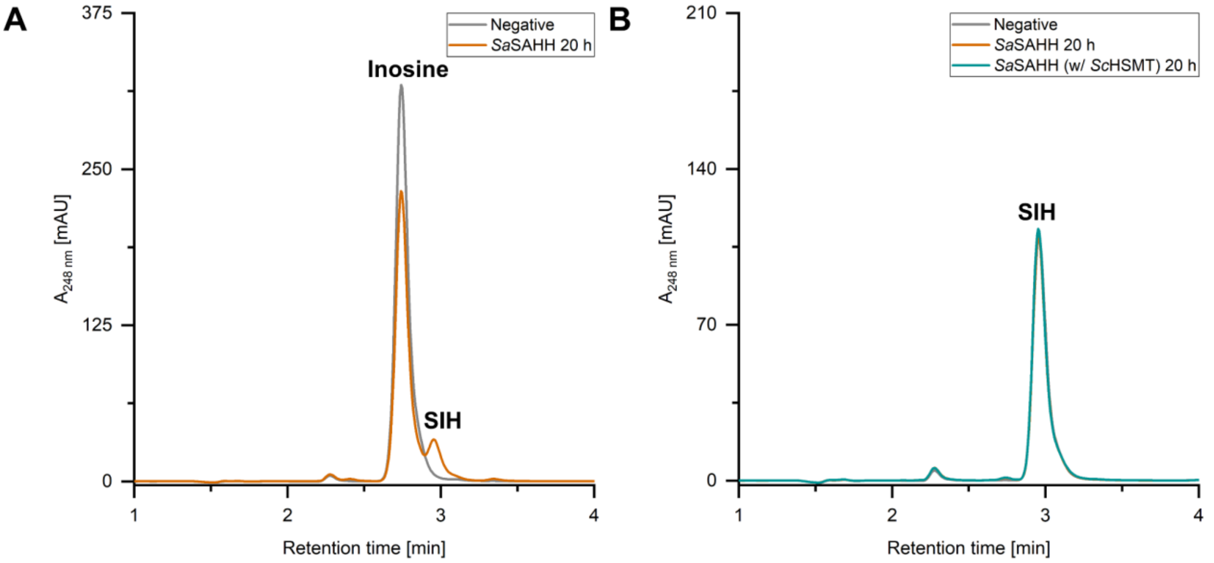

Figure S17B. HPLC chromatograms showing **A.** the SIH synthesis reaction and **B.** the SIH cleavage reaction (with and without the addition of *Sc*HSMT) catalysed by *Sa*SAHH.

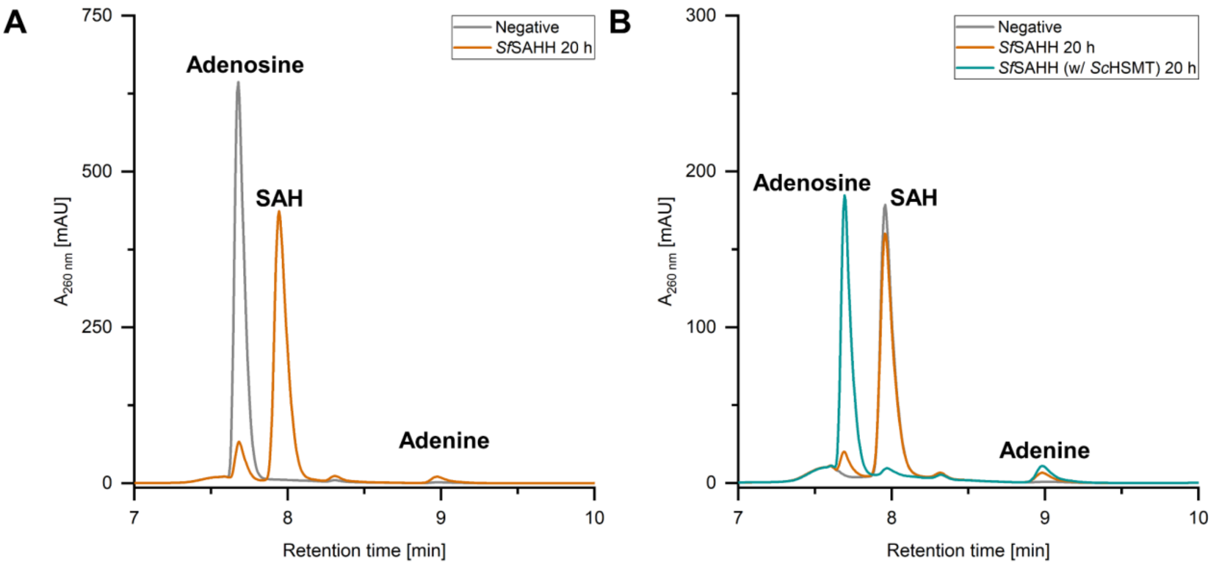

Figure S18A. HPLC chromatograms showing **A.** the SAH synthesis reaction and **B.** the SAH cleavage reaction (with and without the addition of *Sc*HSMT) catalysed by *Sf*SAHH.

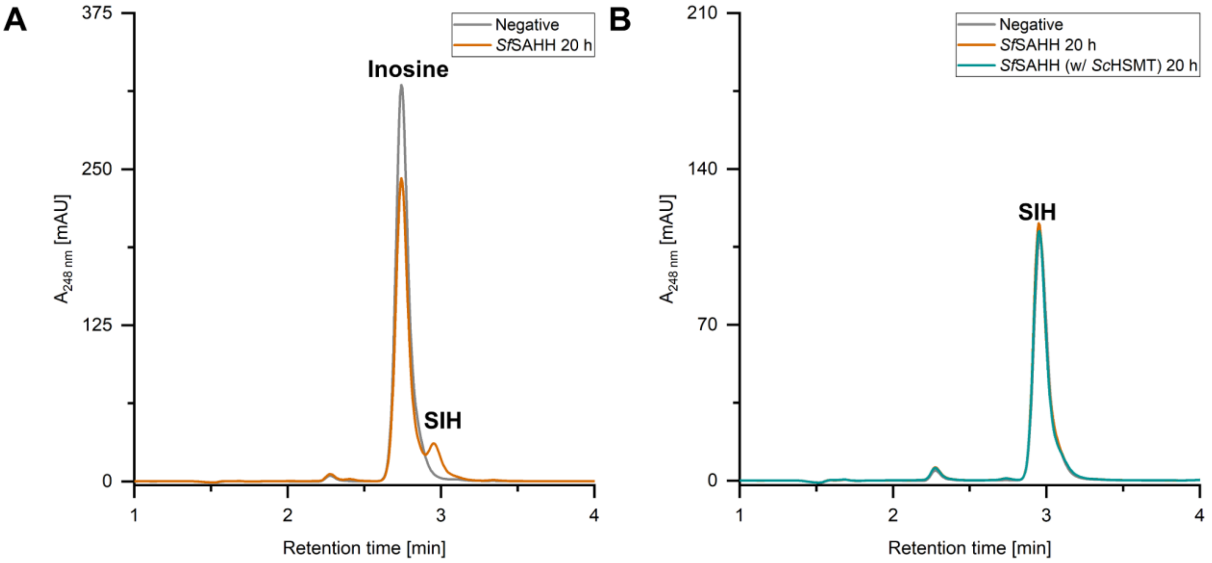

Figure S18B. HPLC chromatograms showing **A.** the SIH synthesis reaction and **B.** the SIH cleavage reaction (with and without the addition of *Sc*HSMT) catalysed by *Sf*SAHH.

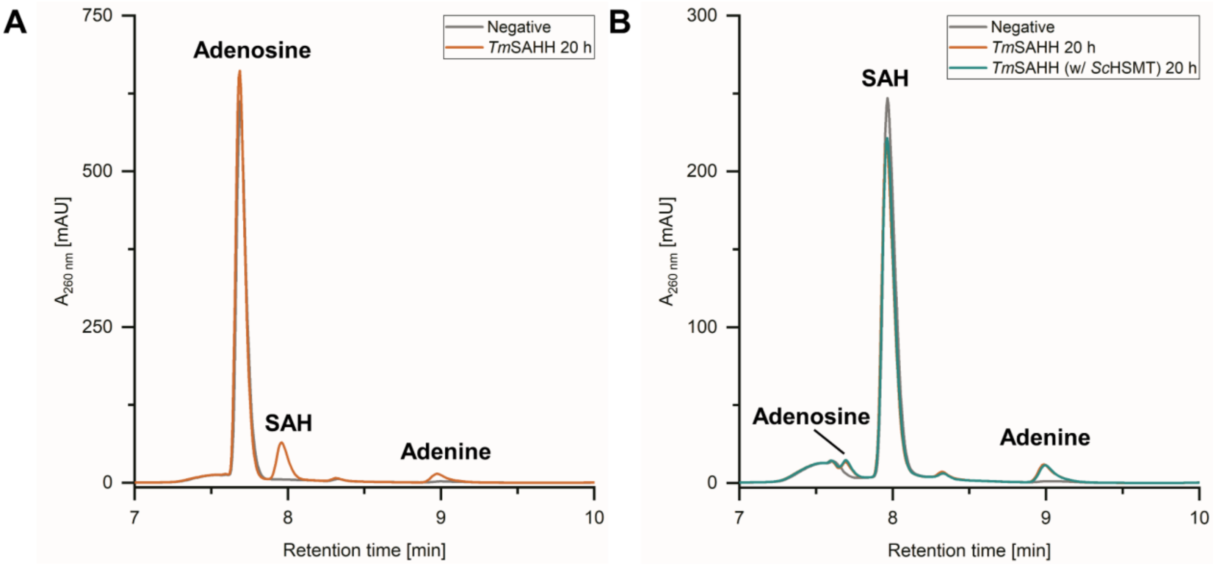

Figure S19A. HPLC chromatograms showing **A.** the SAH synthesis reaction and **B.** the SAH cleavage reaction (with and without the addition of *Sc*HSMT) catalysed by *Tm*SAHH.

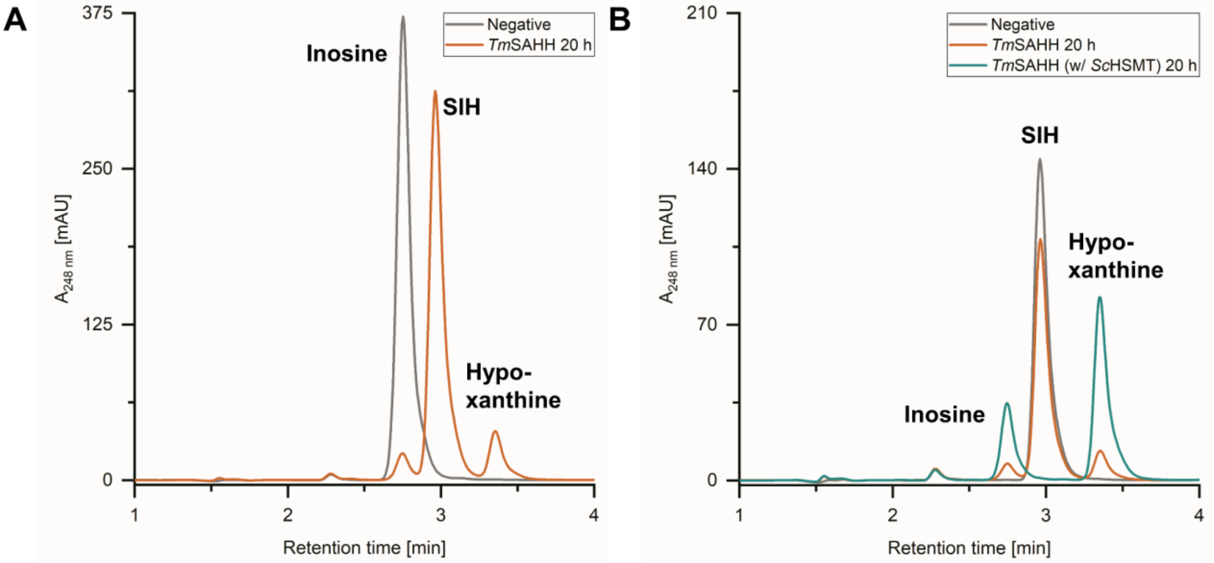

Figure S19B. HPLC chromatograms showing **A.** the SIH synthesis reaction and **B.** the SIH cleavage reaction (with and without the addition of *Sc*HSMT) catalysed by *Tm*SAHH.

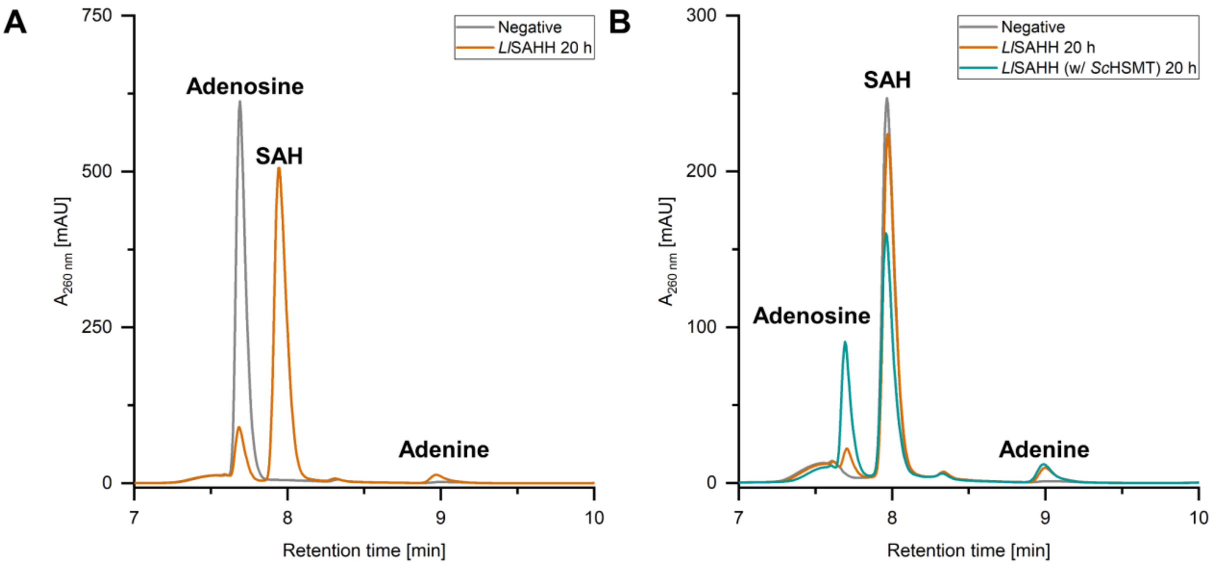

Figure S20A. HPLC chromatograms showing **A.** the SAH synthesis reaction and **B.** the SAH cleavage reaction (with and without the addition of *Sc*HSMT) catalysed by *Ll*SAHH.

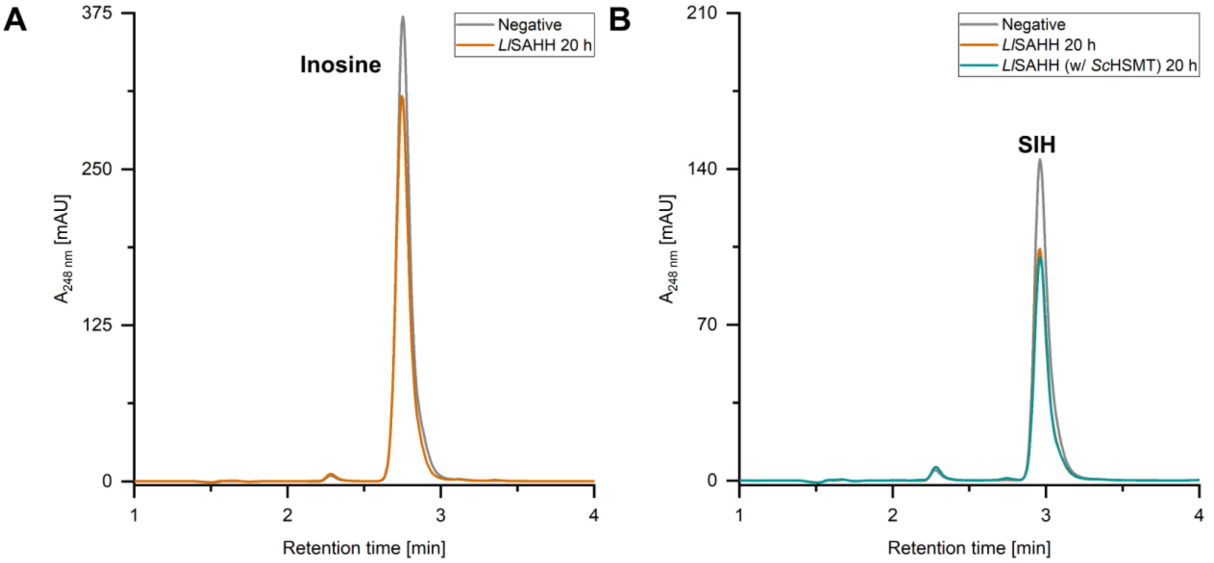

Figure S20B. HPLC chromatograms showing **A.** the SIH synthesis reaction and **B.** the SIH cleavage reaction (with and without the addition of *Sc*HSMT) catalysed by *Ll*SAHH.

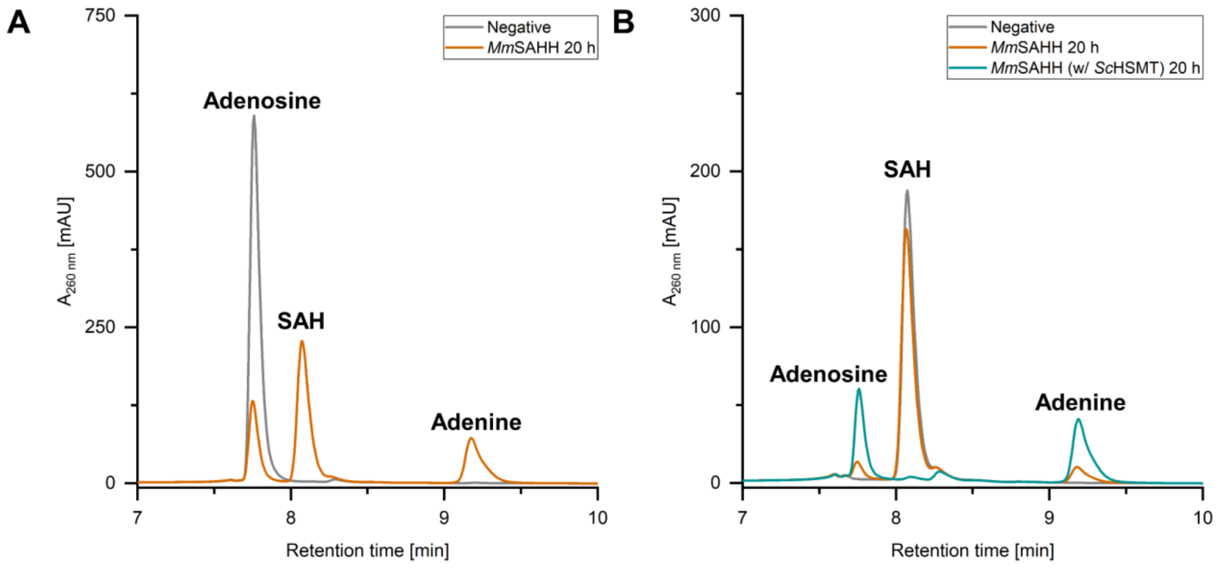

Figure S21A. HPLC chromatograms showing **A.** the SAH synthesis reaction and **B.** the SAH cleavage reaction (with and without the addition of *Sc*HSMT) catalysed by *Mm*SAHH.

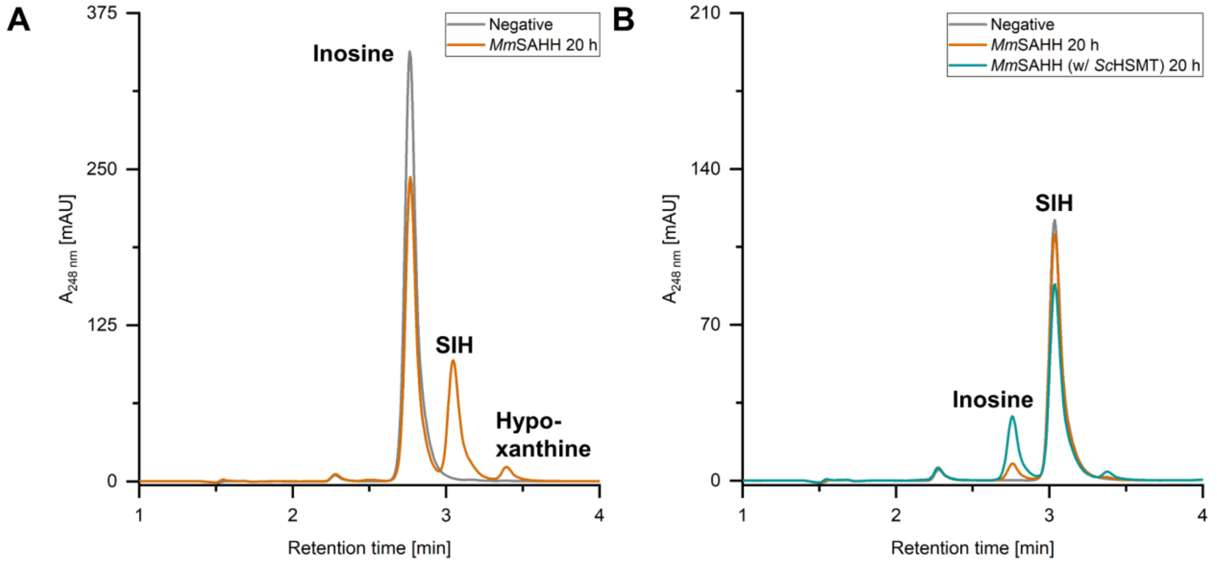

Figure S21B. HPLC chromatograms showing **A.** the SIH synthesis reaction and **B.** the SIH cleavage reaction (with and without the addition of *Sc*HSMT) catalysed by *Mm*SAHH.

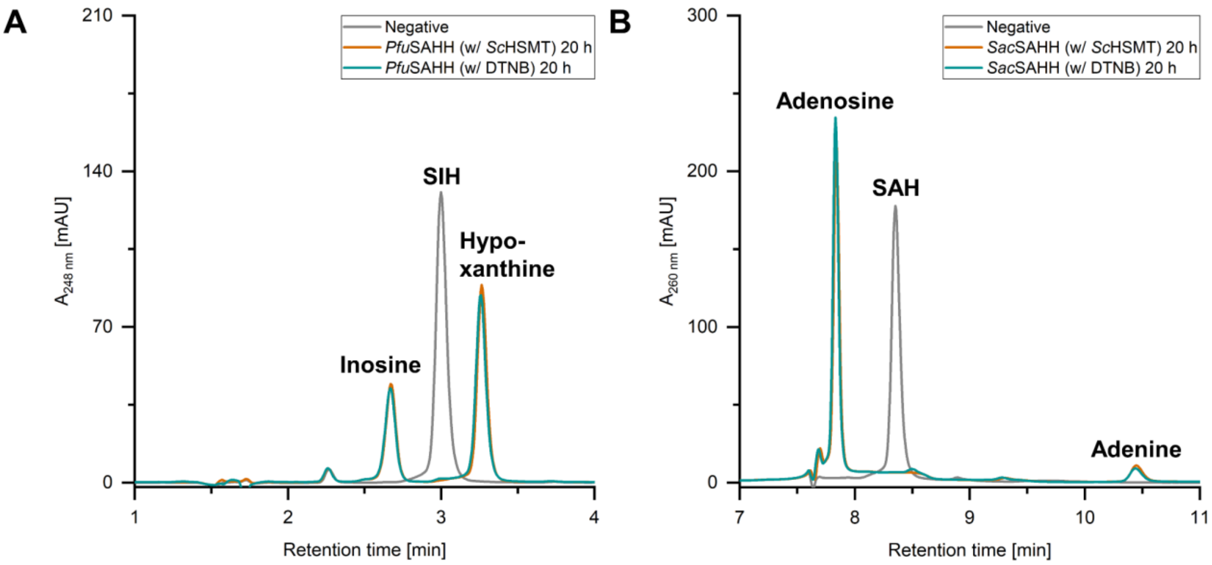

Figure S22. HPLC chromatograms showing **A.** the SIH cleavage reaction (with the addition of either *Sc*HSMT or DTNB) catalysed by *Pfu*SAHH and **B.** the SAH cleavage reaction (with the addition of either *Sc*HSMT or DTNB) catalysed by *Sac*SAHH.

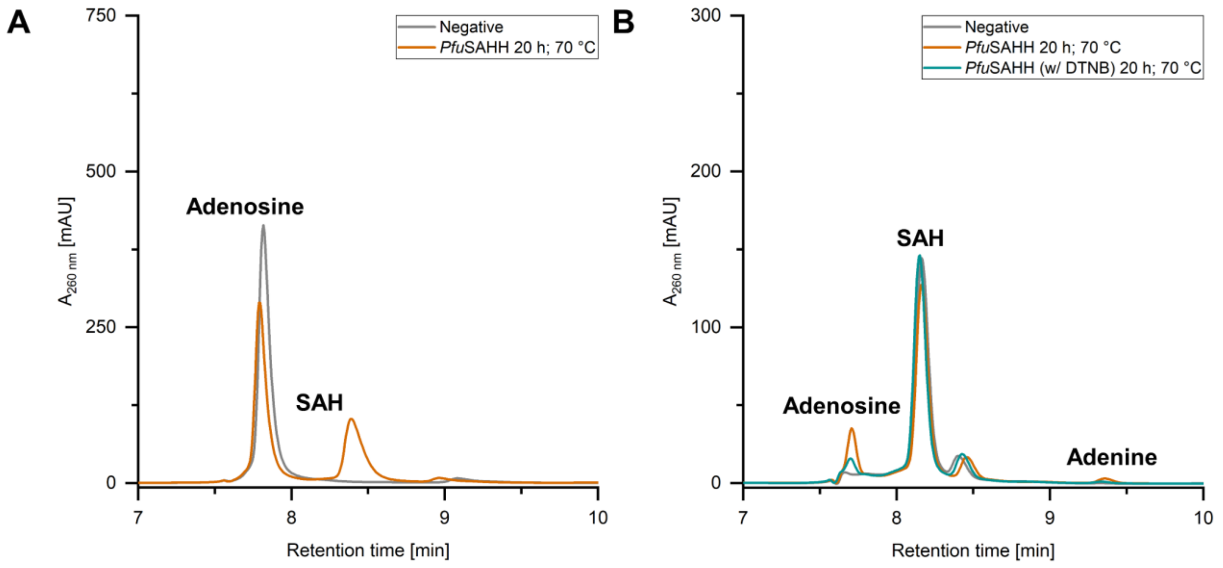

Figure S23A. HPLC chromatograms showing **A.** the SAH synthesis reaction and **B.** the SAH cleavage reaction (with and without the addition of DTNB) catalysed by *Pfu*SAHH at 70 °C.

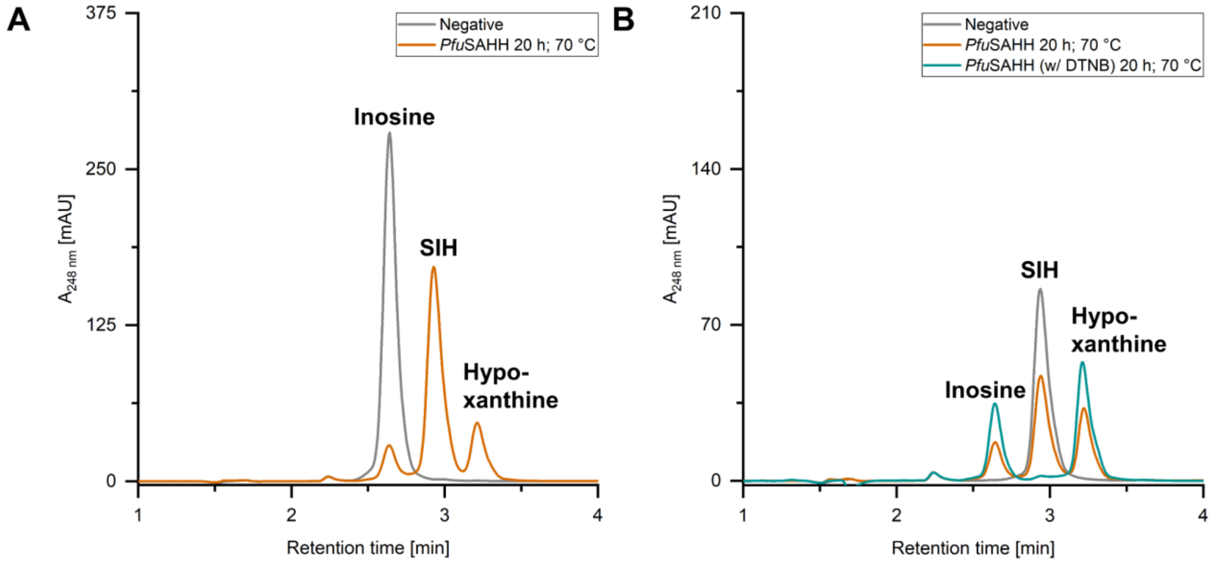

Figure S23B. HPLC chromatograms showing **A.** the SIH synthesis reaction and **B.** the SIH cleavage reaction (with and without the addition of DTNB) catalysed by *Pfu*SAHH at 70 °C.

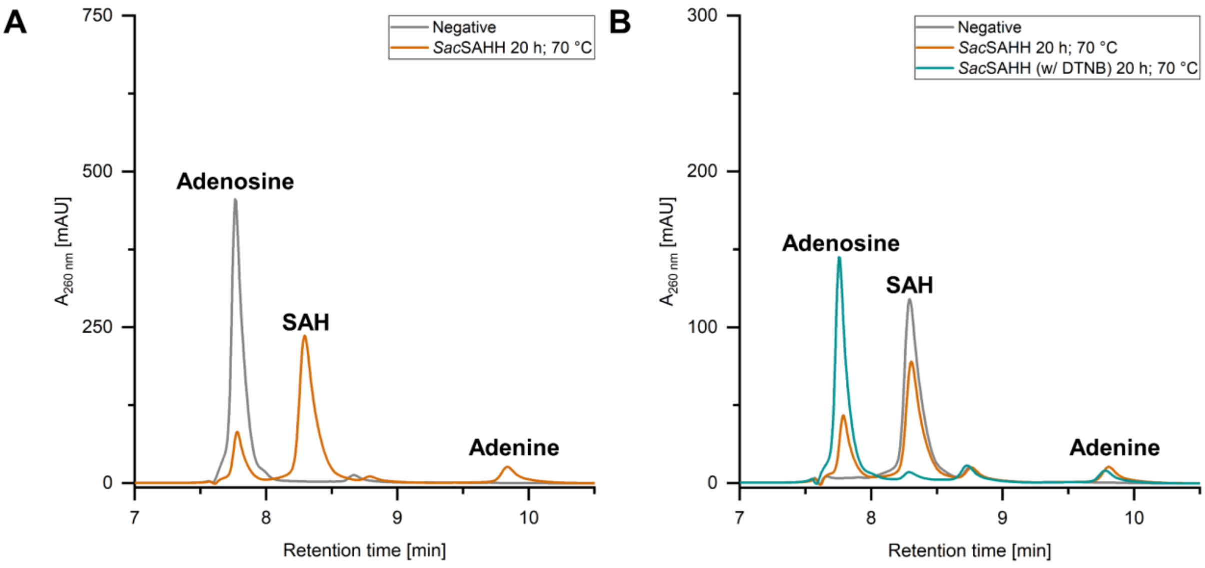

Figure S24A. HPLC chromatograms showing **A.** the SAH synthesis reaction and **B.** the SAH cleavage reaction (with and without the addition of DTNB) catalysed by *Sac*SAHH at 70 °C.

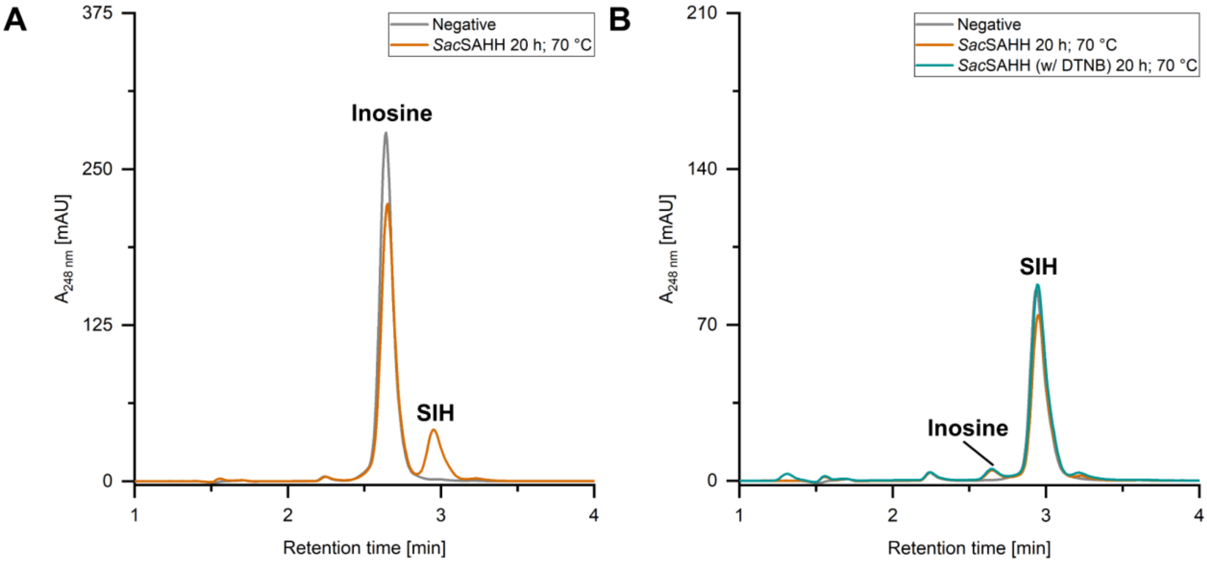

Figure S24B. HPLC chromatograms showing **A.** the SIH synthesis reaction and **B.** the SIH cleavage reaction (with and without the addition of DTNB) catalysed by *Sac*SAHH at 70 °C.

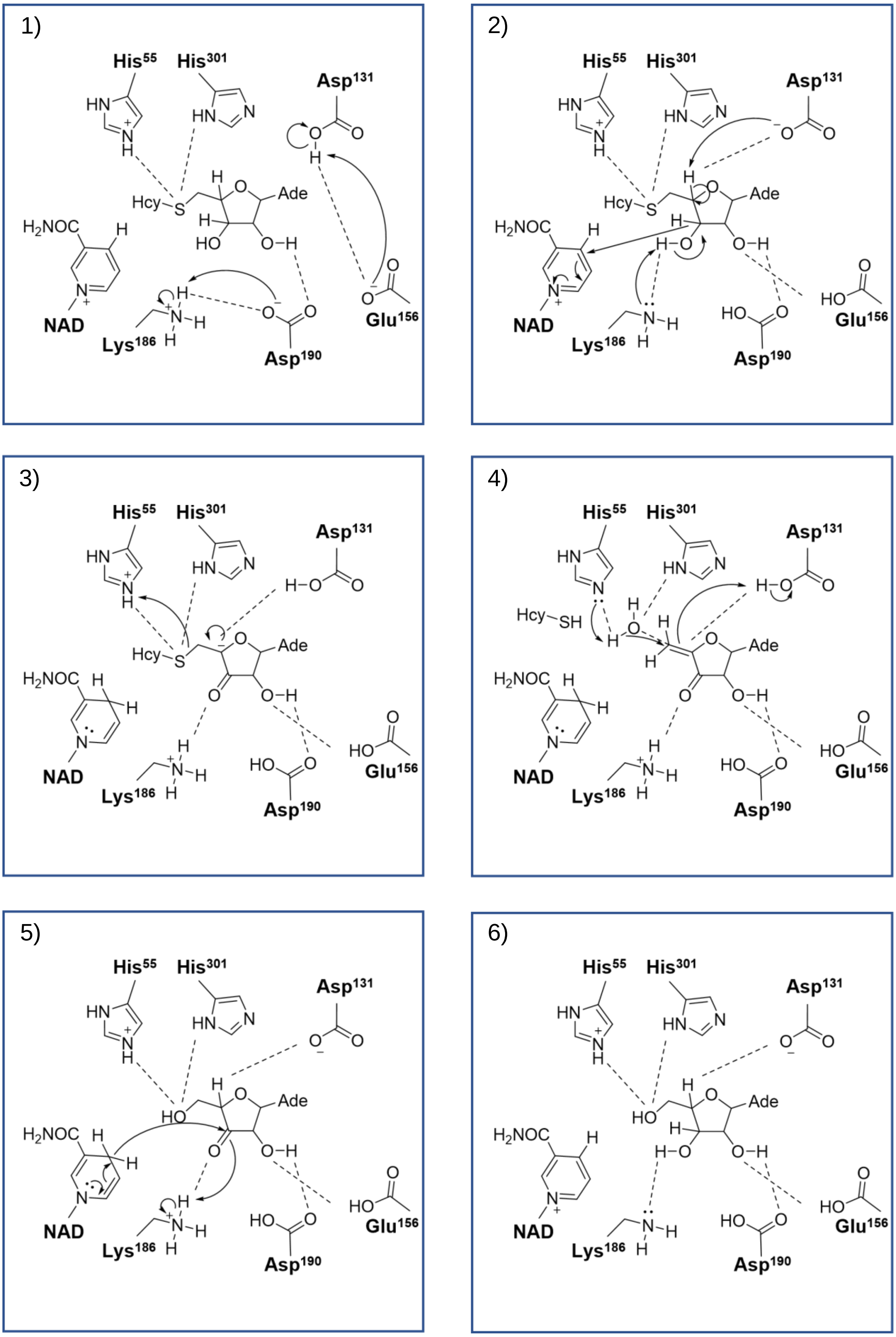

Figure S25. Molecular mechanism of (*Mm*SAHH-catalysed) SAH cleavage. 1) Interactions between amino acid residues before the catalytic reaction. 2) Formation of the 3´-keto intermediate and abstraction of the 4´-proton leading to the carbanion intermediate. 3) Formation of the 3´-keto-4´,5´dehydro intermediate after elimination of L-homocysteine. 4) Nucleophilic attack of an activated water molecule resulting in 3´-keto-adenosine. 5) Reduction of the 3´-keto-adenosine intermediate.6) Final product, adenosine still bound in the active site.

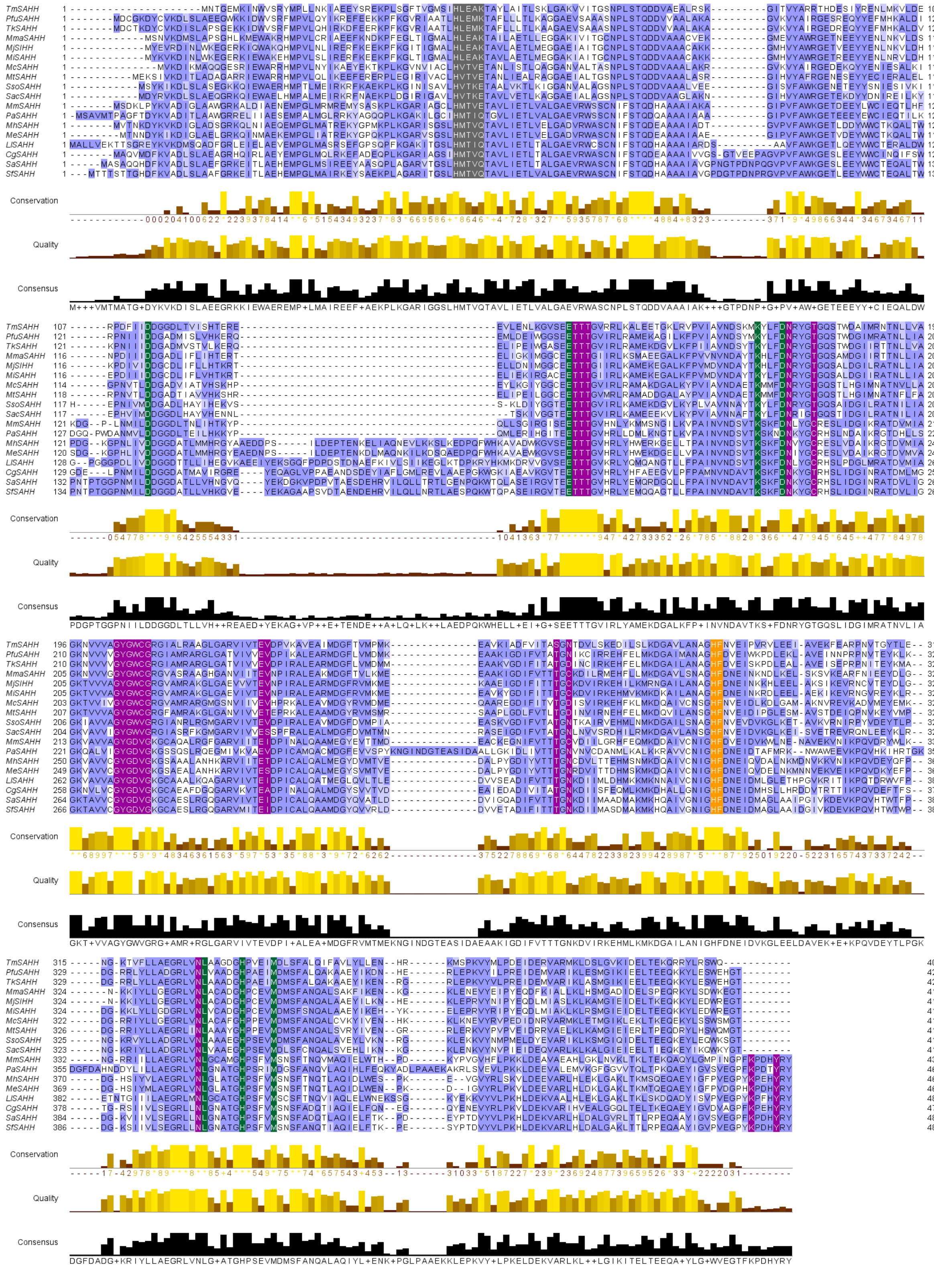

Figure S26. Multiple sequence alignment of selected SAHHs/SIHHs. Residues forming the sequence signature for binding of the nucleobase are coloured in light green. The other residues interacting with the substrate are coloured in dark green while the residues interacting with the cofactor NAD+ are in purple. His and Phe of the molecular gate are coloured orange. The alignment was calculated in Clustal Omega[35] and the figure was prepared using Jalview Version2[36].

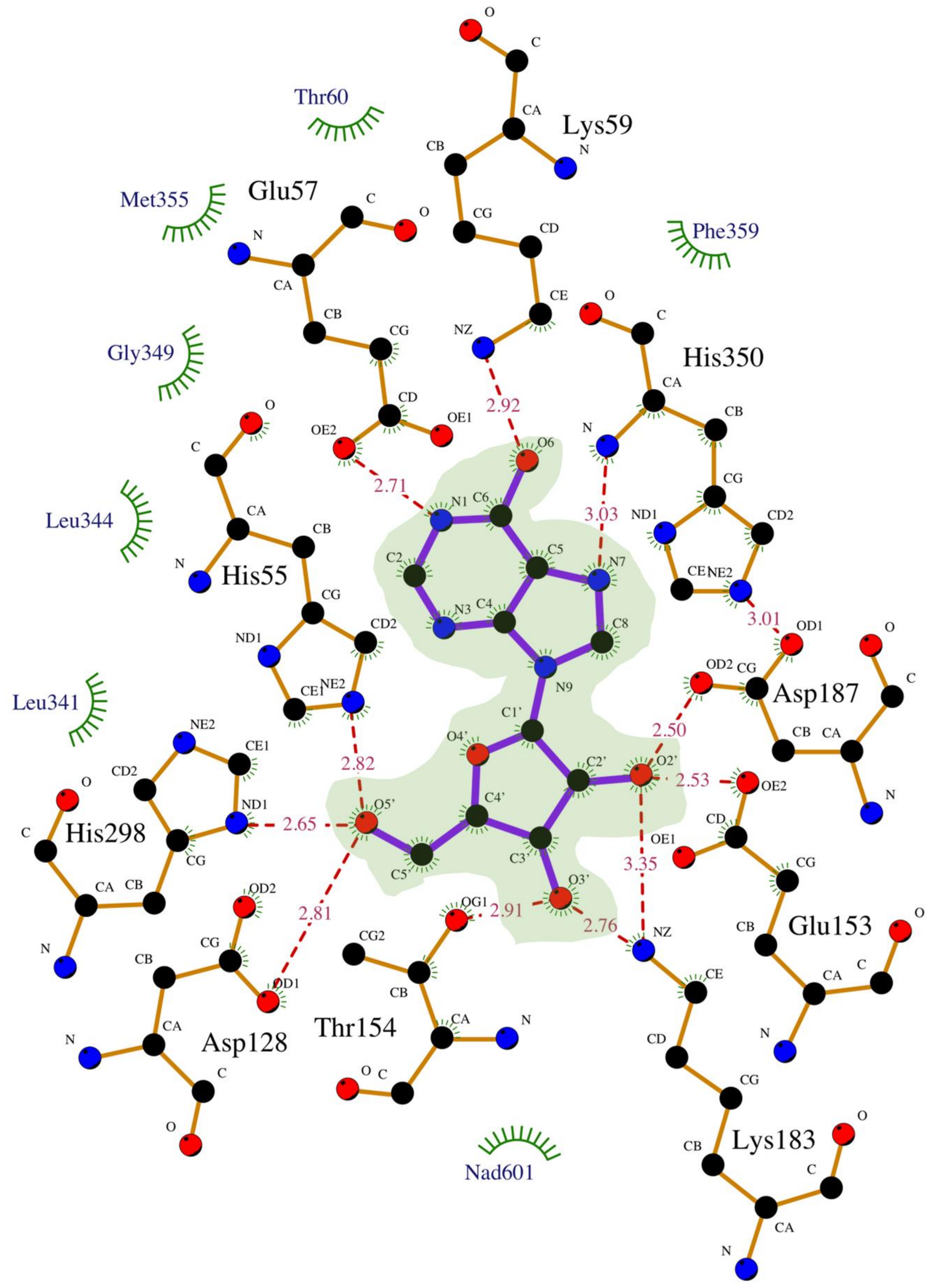

Figure S27A. LigPlot showing the interactions of *Pfu*SAHH-bound inosine (PDB ID: 7R37 chain A) with the amino acid backbones and side chains in the active site.

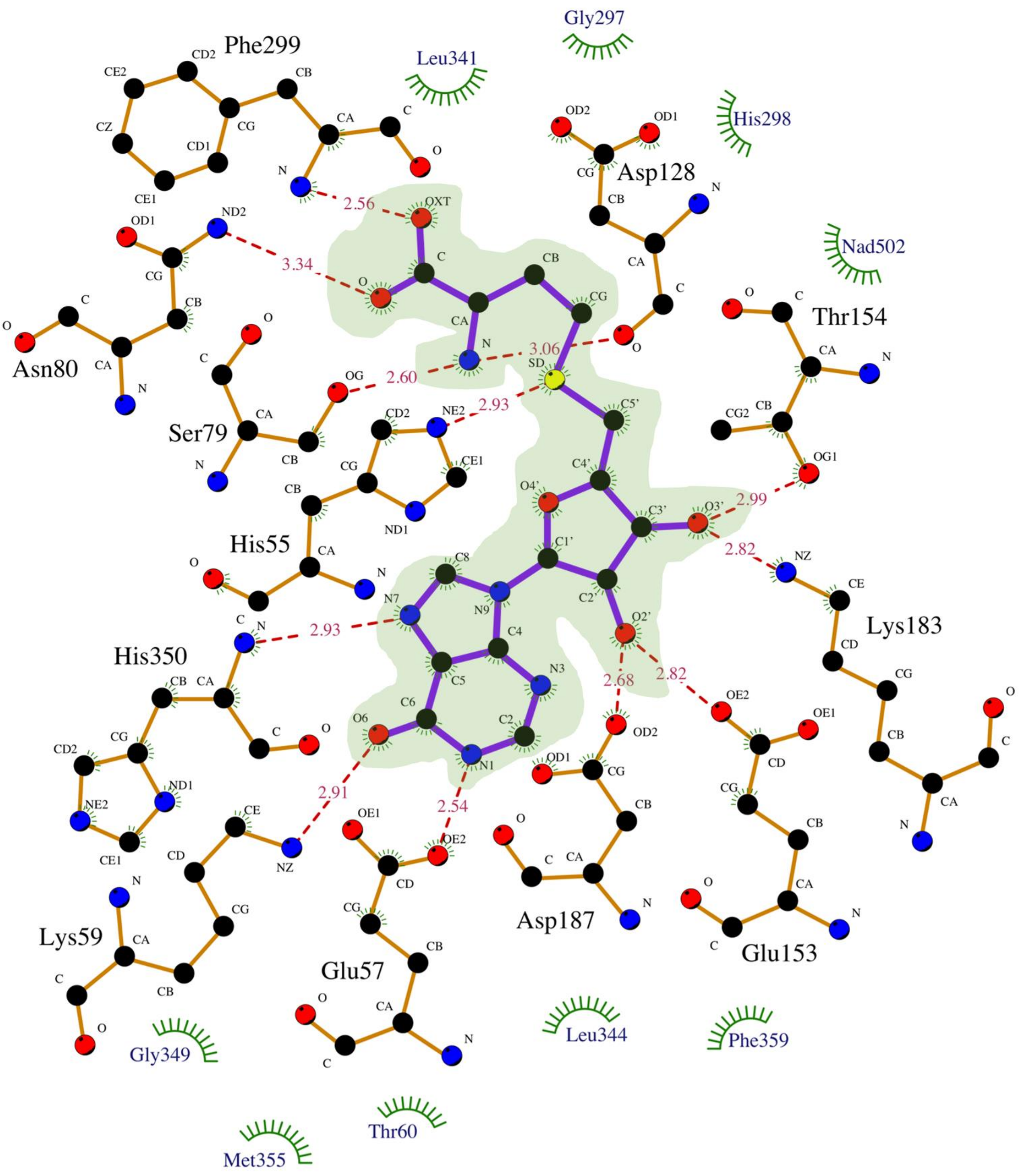

Figure S27B. LigPlot showing the interactions of *Pfu*SAHH-bound SIH PDB ID: 7R38 chain A) with the amino acid backbones and side chains in the active site.

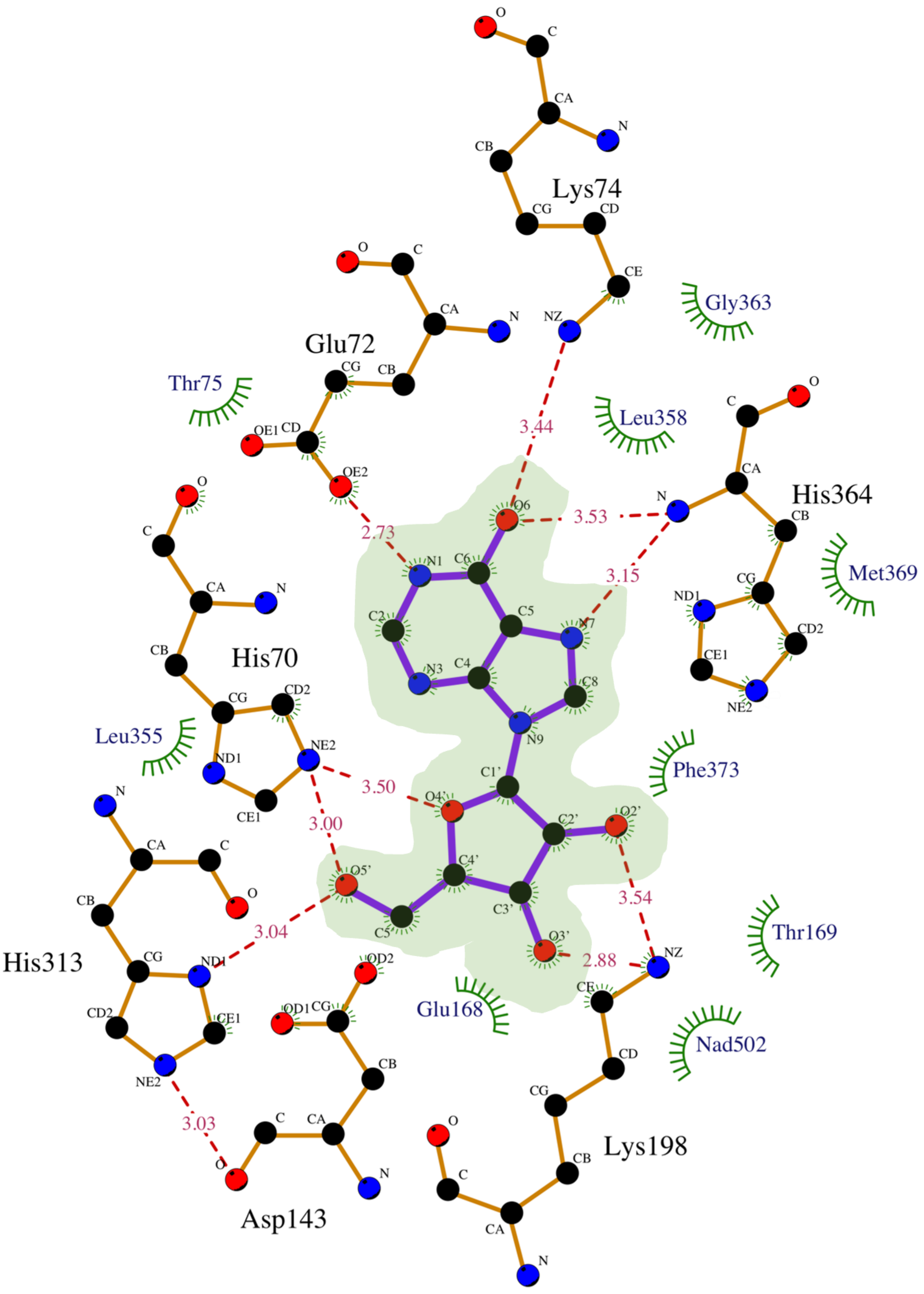

Figure S27C. LigPlot showing the interactions of *Mma*SAHH-bound inosine (PDB ID: 7R3A chain A) with the amino acid backbones and side chains in the active site.

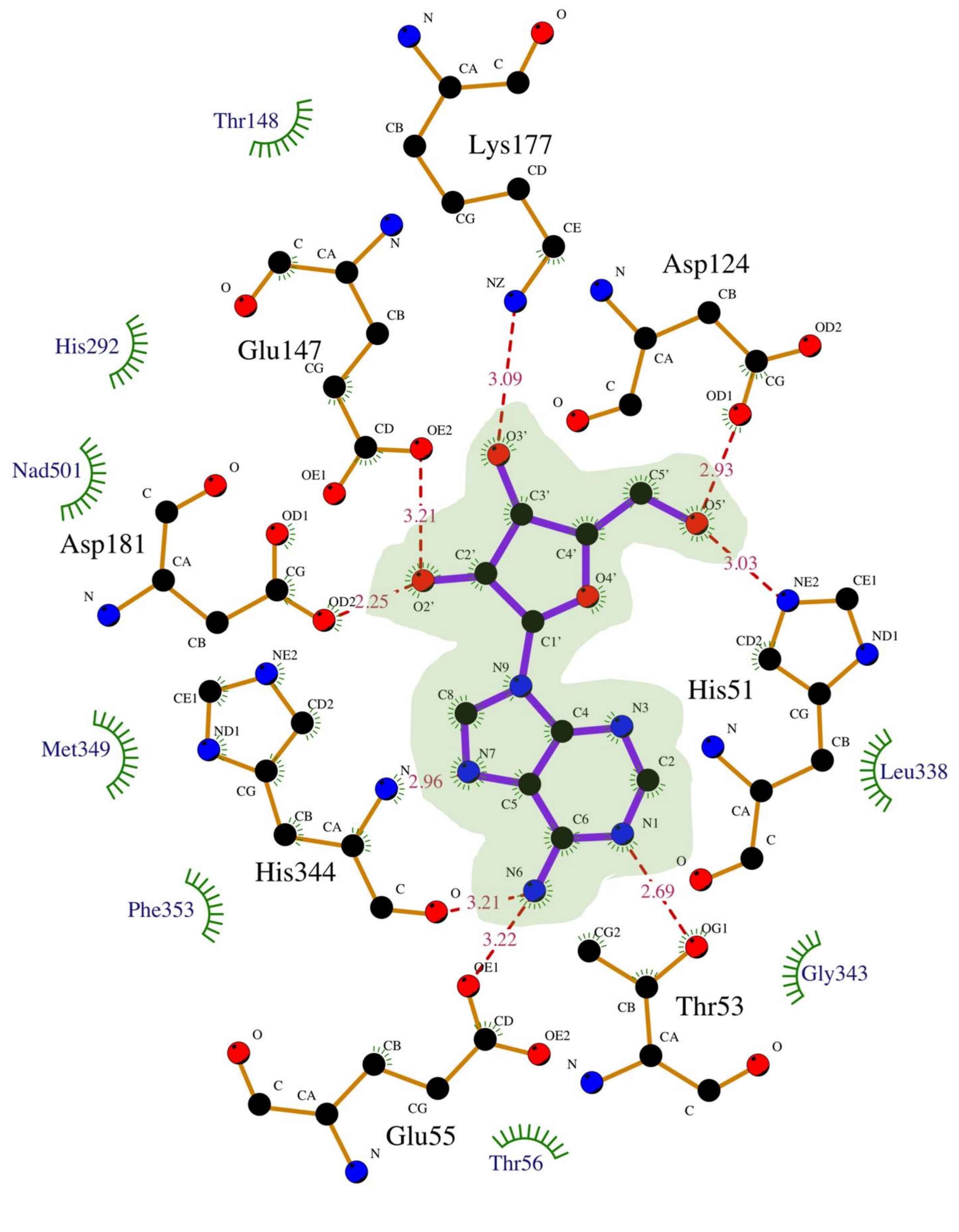

Figure S27D. LigPlot showing the interactions of *SacS*AHH-bound adenosine (PDB ID: 7R39 chain A) with the amino acid backbones and side chains in the active site.

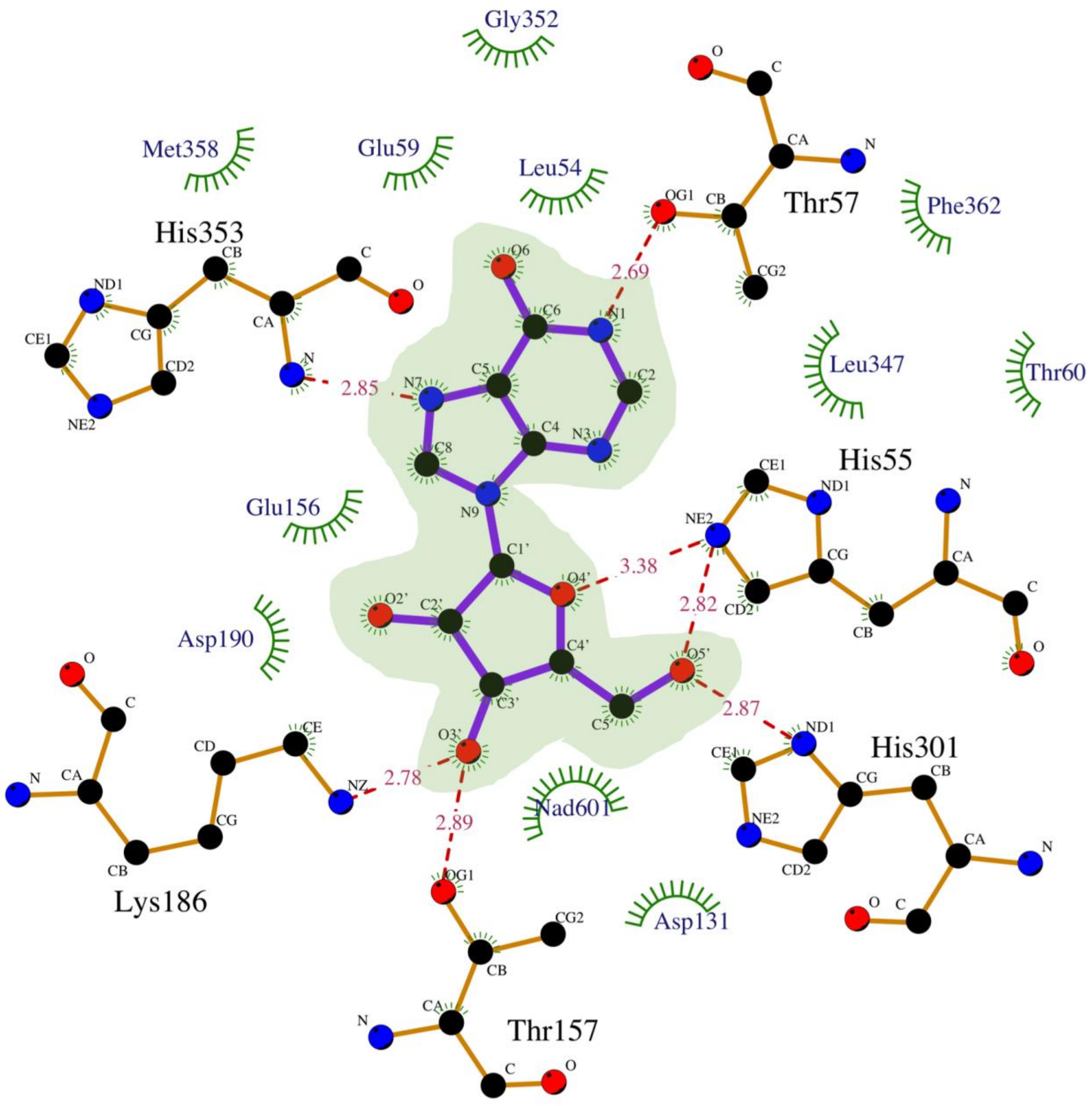

Figure S27E. LigPlot showing the interactions of *Mm*SAHH-bound inosine (PDB ID: 8COD chain A) with the amino acid backbones and side chains in the active site.

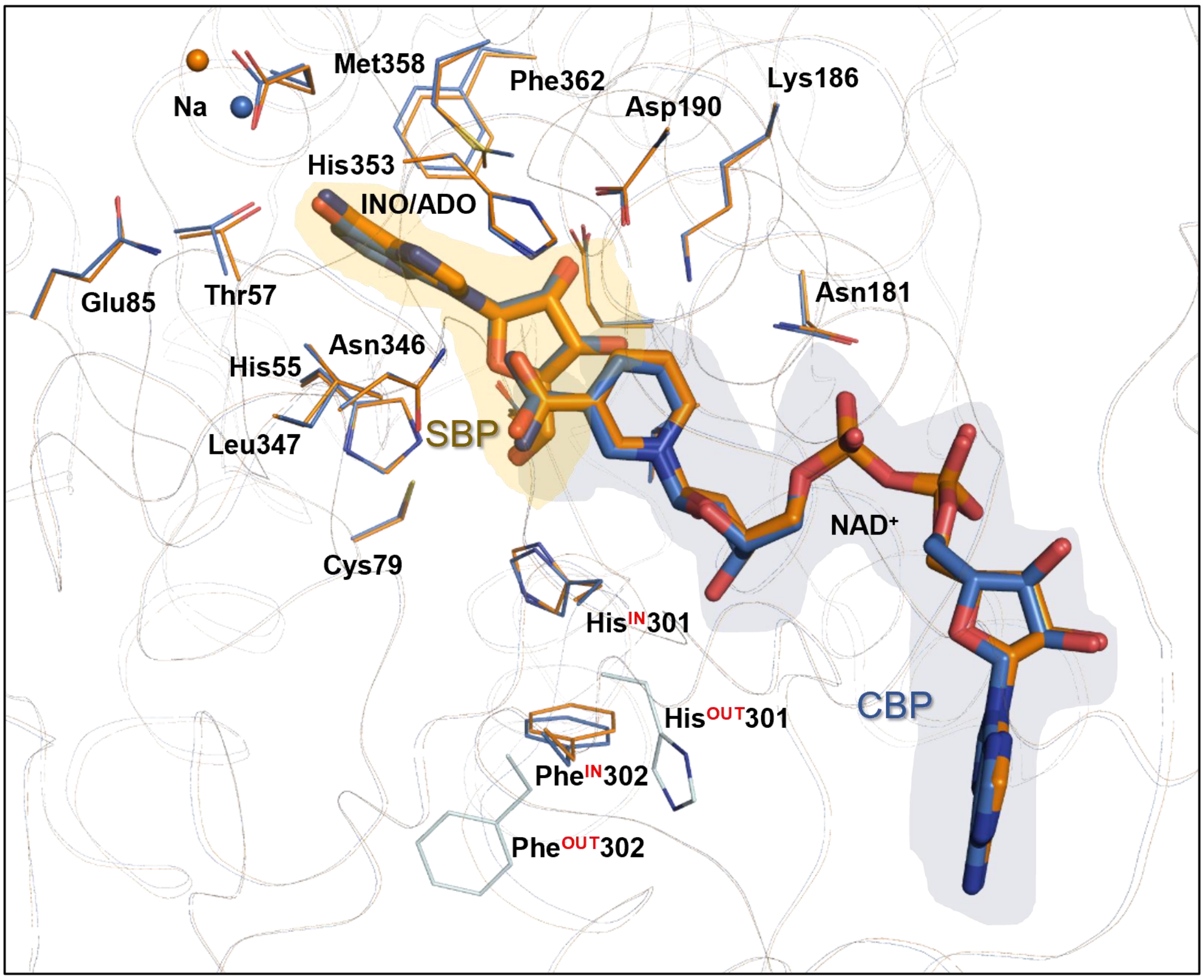

Figure S28. Superposition of the active sites of *Mm*SAHH with adenosine (ADO) in orange (PDB ID: 5AXA[37]) and with inosine (INO) in blue (PDB ID: 8COD) along with NAD^+ cofactors and Na^+ ions (SBP: substrate binding pocket; CBD: Cofactor binding pocket).

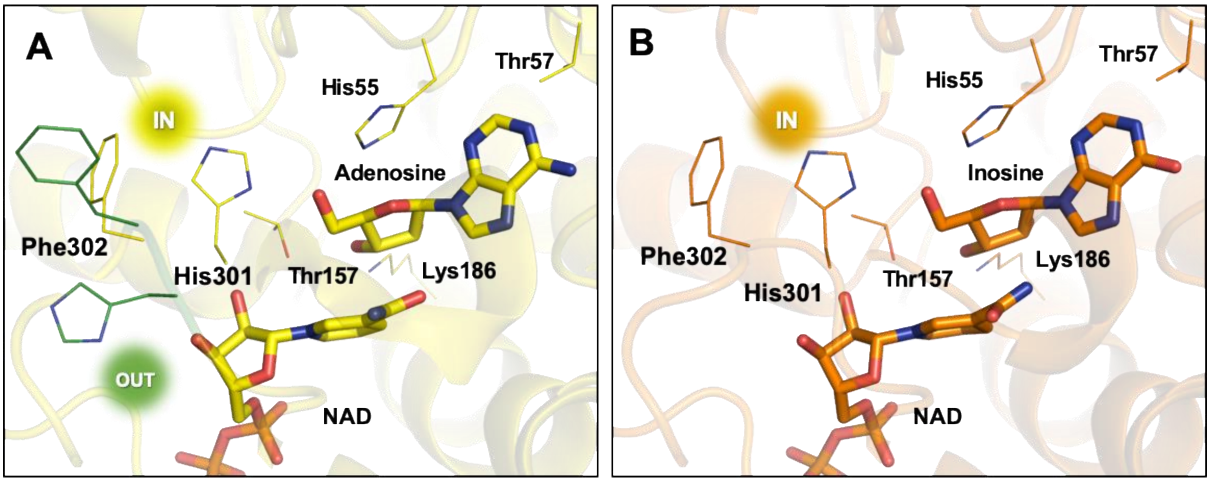

Figure S29. The molecular gate residues in the crystal structures of *Mus musculus* SAHHs. The His residue states are represented as His-IN (IN) and His-OUT (OUT). **A.** In the *Mm*SAHH•NAD•Adenosine complex (PDB ID: 5AXA[37]), the gatekeeper residue forms both IN and OUT conformation leaving the channel gate shut and open, respectively. **B.** In the *Mm*SAHH•NAD•inosine complex (PDB ID: 8COD), the His gatekeeper residue is in the IN orientation thereby closing the channel entrance.

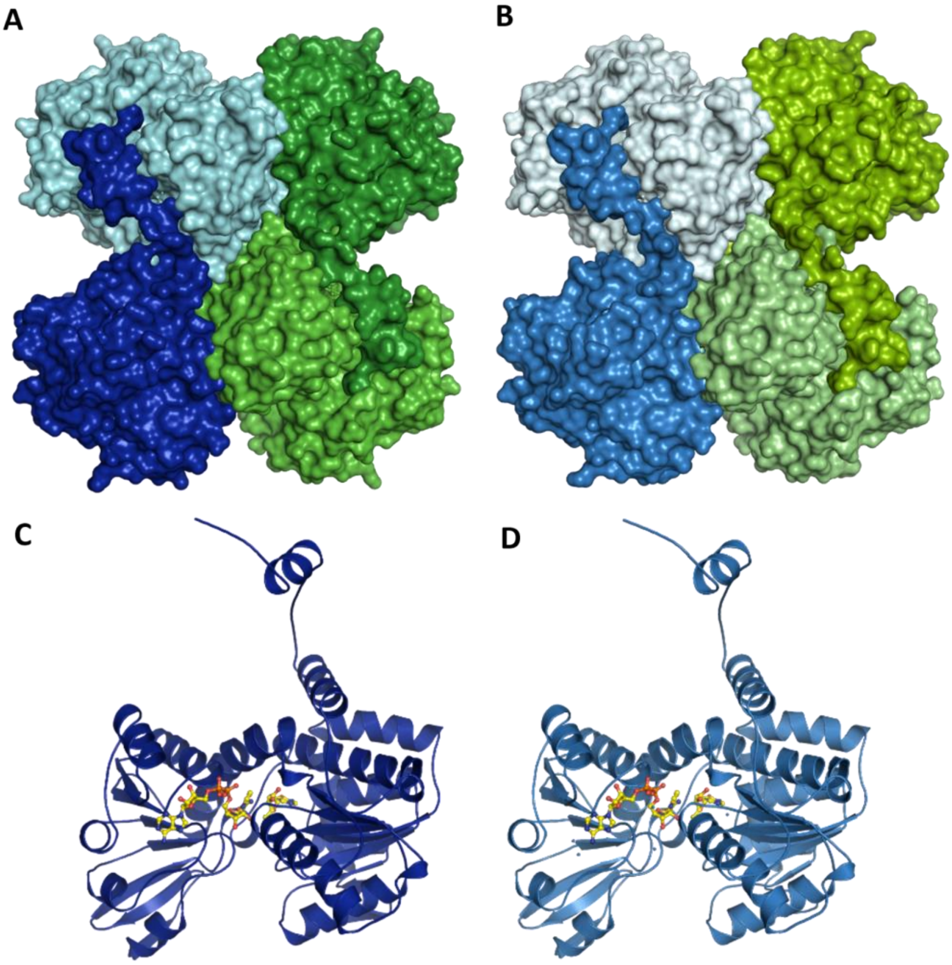

Figure S30. Crystal structures of *Pfu*SAHH treated at different temperatures. **A.** Tetrameric assembly of *Pfu*SAHH treated at 95 °C (PDB ID: 8QNO); **B.** Tetrameric assembly of PfuSAHH treated at room temperature (PDB ID: 7R37). **C.** Monomer of *Pfu*SAHH treated at 95 °C in the closed conformation. **D.** Monomer of *Pfu*SAHH treated at room temperature in the closed conformation.

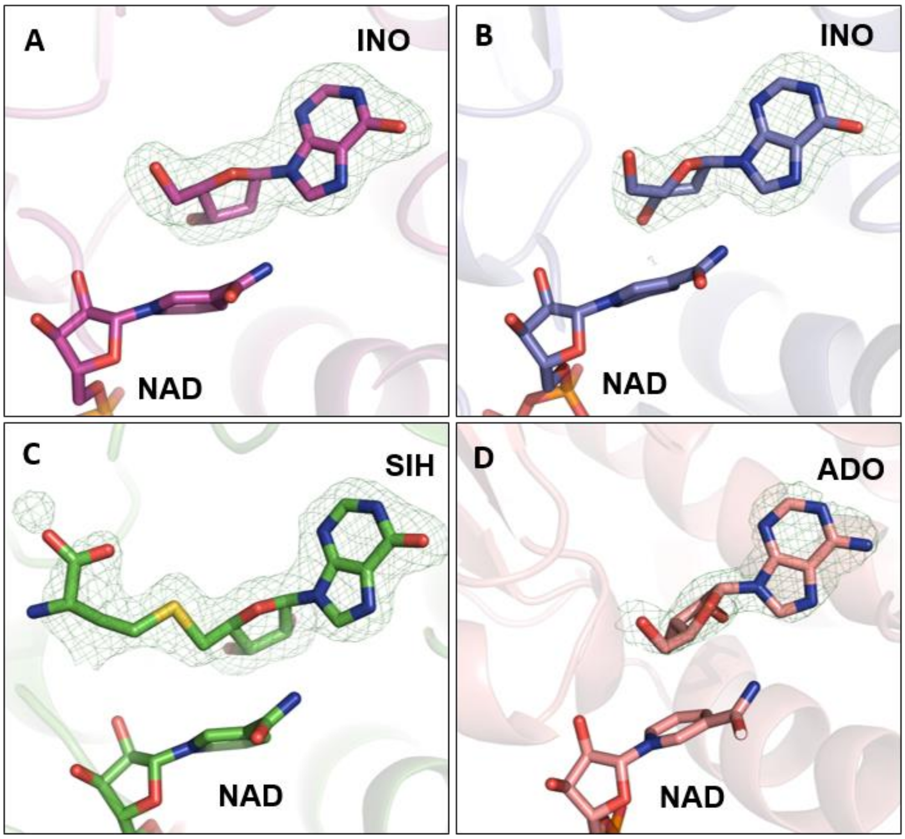

Figure S31. The mFo-DFc polder OMIT difference electron density maps (green mesh) of the bound nucleosides and are contoured at 3.0σ above the mean. Mode of inosine (INO) in the *Pfu*SAHH active site (A) (PDB ID: 7R37), *Mma*SAHH active site (B) (PDB ID: 7R3A), SIH at *Pfu*SAHH active site (C) (PDB ID: 7R38) and adenosine (ADO) in *Sac*SAHH active site (D) (PDB ID: 7R39).

	Tm	*Tk*	*Pfu*	*Mma*	*Mj*	*Mi*	*Mc*	*Mt*	*Sac*	*Sso*	*Pa*	*Mh*	*Me*	*Mm*	*Ll*	*Cg*	*Sf*	*Sa*
Tm	-																	
Tk	58.9	-																
Pfu	59.9	88.1	-															
Mma	55.8	60.5	59.5	-														
Mj	56.8	62.9	63.6	75.7	-													
Mi	56.4	65.6	65.1	75.2	87.2	-												
Mc	49.8	61.8	62.3	55.5	58.1	57.5	-											
Mt	51.5	61.2	61.0	55.4	58.6	58.2	71.0	-										
Sac	53.0	57.6	59.0	56.2	59.1	58.5	56.6	60.0	-									
Sso	53.4	60.3	61.3	57.0	59.9	60.2	58.4	57.9	72.1	-								
Pa	37.0	38.1	38.1	36.2	39.1	38.3	38.5	38.4	40.8	41.4	-							
Mh	40.3	42.5	43.0	41.9	41.2	44.0	43.5	41.8	45.5	46.8	54.4	-						
Me	43.0	43.3	42.8	40.4	42.1	44.2	44.2	42.8	46.7	47.0	55.7	87.8	-					
Mm	42.3	45.4	46.8	43.3	44.6	45.7	46.6	44.5	47.5	49.6	58.9	65.8	65.5	-				
Ll	38.0	40.5	42.1	43.2	43.0	43.6	40.7	41.5	44.2	45.4	52.2	63.0	61.6	62.7	-			
Cg	41.3	43.7	43.7	42.4	42.7	42.8	41.1	41.6	47.7	48.0	54.3	59.6	59.1	58.5	58.6	-		
Sf	40.1	43.0	43.7	42.2	43.9	44.2	44.0	43.8	46.8	46.3	57.4	63.6	63.1	64.6	65.7	67.6	-	
Sa	39.6	42.8	43.5	41.9	42.9	44.0	43.7	44.5	46.5	46.3	58.2	62.1	62.4	64.4	65.1	68.2	89.3	-

Figure S32. Sequence identities given in percent [%] based on the amino acid sequence for all investigated SAHHs in this work. The matrix was calculated using Clustal Omega[35]

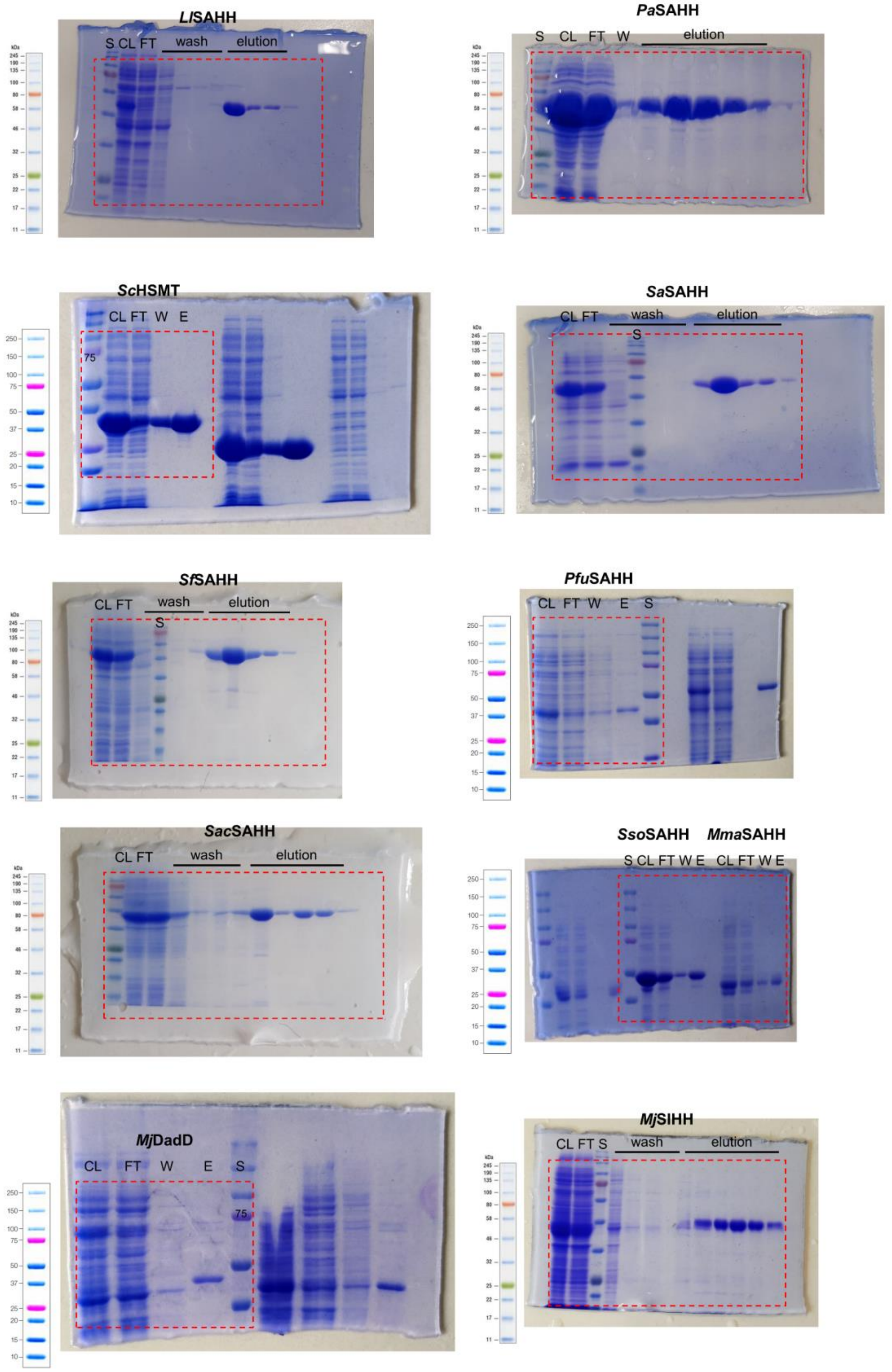

Figure S33. Uncropped and unedited SDS gels of the purifications of *Ll*SAHH, *Pa*SAHH, *Sc*HSMT, *Sa*SAHH, *Sf*SAHH, *Pfu*SAHH, *Sac*SAHH, *Sso*SAHH, *Mma*SAHH, *Mj*DadD and *Mj*SIHH.

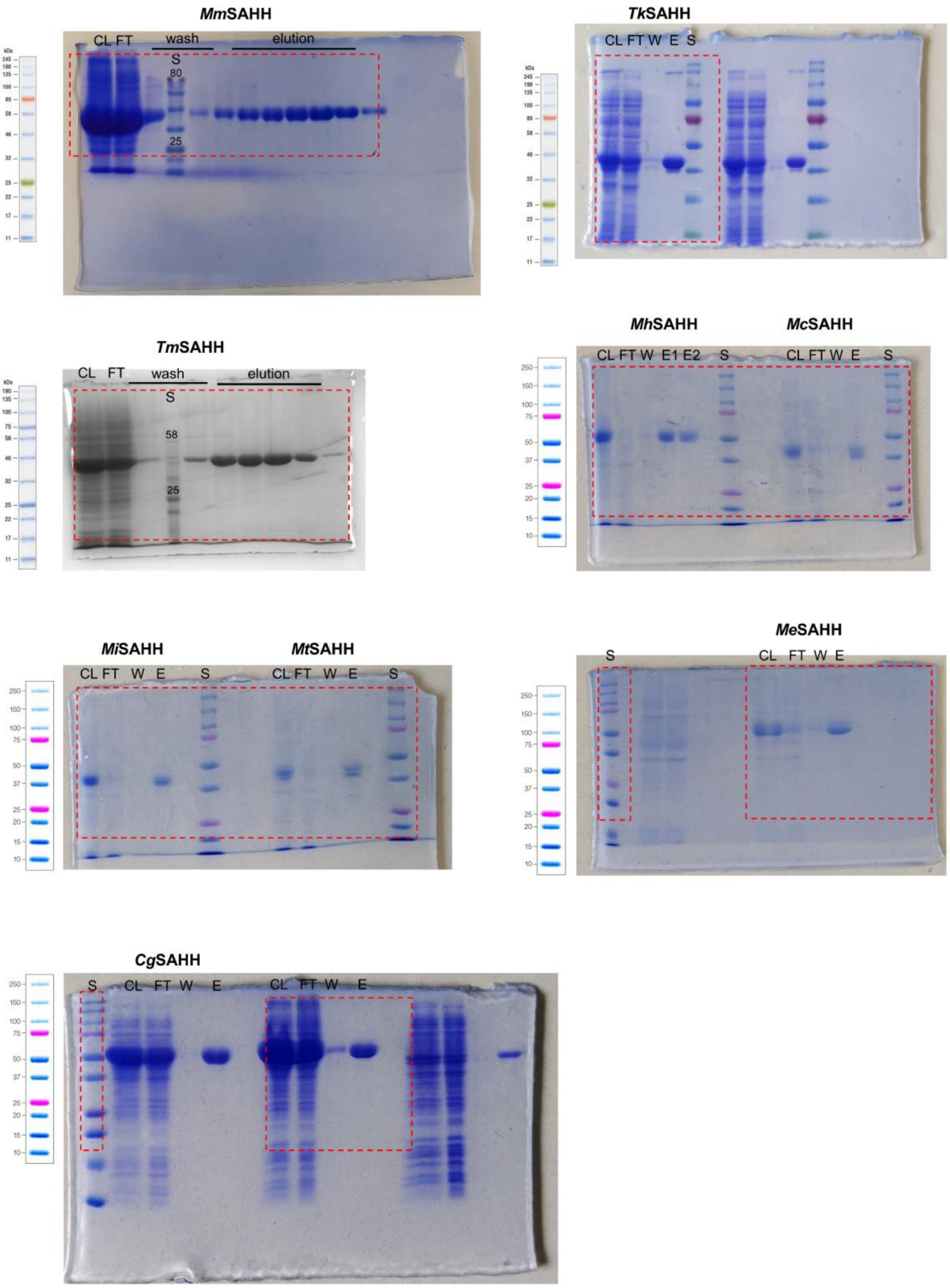

Figure S34. Uncropped and unedited SDS gels of the purifications of *Mm*SAHH, *Tk*SAHH, *Tm*SAHH, *Mh*SAHH, *Mc*SAHH, *Mi*SAHH, *Mt*SAHH, *Me*SAHH and *Cg*SAHH.

Supplementary Notes 1

DNA sequences of the enzymes used in this study.

>*Sc*HSMT

ATGAAGCGCATTCCAATCAAAGAACTAATAGTTGAGCACCCCGGAAAAGTTCTTATCCTTGATGGTGGACAGGGTACAGA
ATTGGAAAACAGAGGCATTAACATAAATAGTCCGGTATGGTCTGCAGCTCCTTTTACGAGCGAATCCTTTTGGGAGCCATC
TTCTCAAGAGCGAAAGGTGGTAGAAGAAATGTACAGAGACTTTATGATTGCTGGCGCAAACATATTAATGACAATTACTT
ACCAGGCAAACTTTCAAAGCATATCTGAGAATACCTCGATTAAAACTCTGGCTGCTTACAAGCGTTTTCTCGATAAAATCG
TGTCATTTACTCGTGAATTTATTGGTGAGGAAAGGTACTTAATCGGGAGTATTGGCCCATGGGCAGCACATGTATCCTGTG
AATATACTGGTGACTATGGTCCCCATCCTGAGAATATTGATTACTACGGCTTTTTCAAACCCCAGCTGGAGAACTTCAACCA
AAATAGAGATATTGATCTTATTGGTTTTGAAACGATTCCAAATTTTCATGAGTTAAAGGCTATTTTATCCTGGGATGAAGAT
ATTATTTCGAAGCCCTTTTATATTGGGTTGTCGGTGGATGACAATAGTTTGCTACGAGACGGTACCACTTTGGAAGAAATT
TCTGTCCATATAAAAGGCCTCGGAAATAAAATTAACAAGAATCTCTTATTAATGGGAGTTAACTGTGTCAGTTTCAATCAA
TCGGCATTAATTCTTAAAATGTTGCACGAGCATCTACCTGGCATGCCTCTGCTAGTTTACCCAAACAGTGGAGAAATCTAC
AATCCCAAAGAGAAGACATGGCACCGGCCGACTAATAAGTTGGATGACTGGGAGACCACGGTTAAGAAATTCGTTGATA
ATGGTGCGCGCATTATTGGCGGTTGTTGTAGAACGTCTCCTAAAGATATCGCCGAAATTGCATCAGCTGTAGATAAATACT
CCTAA

>*Mj*DadD (codon-optimised for *E. coli*)

ATGATCCTGATCAAAAACGTGTTTGTGAATGGTAAACGCCAGGATATTCTGATCGAAGGCAACAAGATCAAAAAAATCGG
CGAGGTGAAAAAAGAAGAAATCGAAAACGCCGAAATCATCGACGGCAAAAACAAAATTGCCATTCCGGGTCTGATTAAC
ACCCATACACATATTCCGATGACACTGTTTCGTGGTGTTGCAGATGATCTGCCGCTGATGGAATGGCTGAATAACTATATT
TGGCCGATGGAAGCCAAACTGAACGAAGAAATTGTTTATTGGGGCACCCTGCTGGGTTGTATTGAAATGATTCGTAGCGG
CACCACCACCTTTAATGATATGTATTTTTTCCTGGAAGGGATCGCCAAAGCAGTTGATGAAAGCGGTATGCGTGCAGTTCT
GGCCTATGGTATGATTGACCTGTTTGATGAAGAACGTCGTGAACGCGAACTGAAAAATGCAGAGAAATACATCAACTATA
TCAACAGCCTGAACAACAGCCGTATTATGCCTGCACTGGGTCCGCATGCACCGTATACCTGTAGCAAAGAACTGTTAATG
GAAGTGAATAACCTGGCCAAAAAGTATAACGTGCCGATTCATATTCATCTGAACGAAACCCTGGATGAGATCAAGATGGT
TAAAGAAAAAACCGGTATGGAACCGTTCATCTATCTGAATAGCTTTGGCTTTTTTGATGATGTTCGTGCAATTGCCGCACA
TTGTGTTCATCTGACCGATGAAGAAATCAAGATCATGAAACAGAAAAACATTAACGTGTCGCATAACCCGATTAGCAATCT
GAAACTGGCAAGCGGTGTTGCACCGATTCCGAAACTGCTGGCCGAAGGTATTAATGTTACCCTGGGCACCGATGGTTGTG
GTAGCAATAATAACCTGAACCTGTTCGAAGAGATTAAAGTTAGCGCCATTCTGCATAAAGGCGTTAATCTGAATCCGACC
GTTGTTAAAGCAGAAGAAGCATTTAACTTCGCCACCAAAAAATGGTGCAAAAGCCCTGAACATTAAAGCCGGTGAAATTCG
TGAAGGTTATCTGGCAGATATTGTGCTGATTAATCTGGATAAACCGTATCTGTACCCGAAAGAAAACATTATGAGCCATCT
GGTGTATGCCTTTAATGGCTTCGTGGATGATGTGATTATTGATGGCAACATTGTTATGCGTGATGGCGAAATTCTGACCGT
TGACGAAGAAAAAGTTTACGAAAAAGCCGAAGAGATGTATGAAATTCTGCGCAGCTAA

>*Cg*SAHH

ATGGCACAGGTTATGGACTTCAAGGTTGCCGATCTTTCACTAGCAGAGGCAGGACGTCACCAGATTCGTCTTGCAGAGTA
TGAGATGCCAGGTCTCATGCAGTTGCGCAAGGAATTCGCAGACGAGCAGCCTTTGAAGGGCGCCCGAATTGCTGGTTCTA
TCCACATGACGGTCCAGACCGCCGTGCTTATTGAGACCCTCACTGCTTTGGGCGCTGAGGTTCGTTGGGCTTCCTGCAACA
TTTTCTCCACCCAGGATGAGGCTGCAGCGGCTATCGTTGTCGGCTCCGGCACCGTCGAAGAGCCAGCTGGTGTTCCAGTA
TTCGCGTGGAAGGGTGAGTCACTGGAGGAGTACTGGTGGTGCATCAACCAGATCTTCAGCTGGGGCGATGAGCTGCCAA
ACATGATCCTCGACGACGGCGGTGACGCCACCATGGCTGTTATTCGCGGTCGCGAATACGAGCAGGCTGGTCTGGTTCCA
CCAGCAGAGGCCAACGATTCCGATGAGTACATCGCATTCTTGGGCATGCTGCGTGAGGTTCTTGCTGCAGAGCCTGGCAA
GTGGGGCAAGATCGCTGAGGCCGTTAAGGGTGTCACCGAGGAAACCACCACCGGTGTGCACCGCCTGTACCACTTCGCT
GAAGAAGGCGTGCTGCCTTTCCCAGCGATGAACGTCAACGACGCTGTCACCAAGTCCAAGTTTGATAACAAGTACGGCAC

CCGCCACTCCCTGATCGACGGCATCAACCGCGCCACTGACATGCTCATGGGCGGCAAGAACGTGCTTGTCTGCGGTTACG
GCGATGTCGGCAAGGGCTGCGCTGAGGCTTTCGACGGCCAGGGCGCTCGCGTCAAGGTCACCGAAGCTGACCCAATCAA
CGCTCTTCAGGCTCTGATGGATGGCTACTCTGTGGTCACCGTTGATGAGGCCATCGAGGACGCCGACATCGTGATCACCG
CGACCGGCAACAAGGACATCATTTCCTTCGAGCAGATGCTCAAGATGAAGGATCACGCTCTGCTGGGCAACATCGGTCAC
TTTGATAATGAGATCGATATGCATTCCCTGTTGCACCGCGACGACGTCACCCGCACCACGATCAAGCCACAGGTCGACGA
GTTCACCTTCTCCACCGGTCGCTCCATCATCGTCCTGTCCGAAGGTCGCCTGTTGAACCTTGGCAACGCCACCGGACACCC
ATCATTTGTCATGTCCAACTCTTTCGCCGATCAGACCATTGCGCAGATCGAACTGTTCCAAAACGAAGGACAGTACGAGAA
CGAGGTCTACCGTCTGCCTAAGGTTCTCGACGAAAAGGTGGCACGCATCCACGTTGAGGCTCTCGGCGGTCAGCTCACCG
AACTGACCAAGGAGCAGGCTGAGTACATCGGCGTTGACGTTGCAGGCCCATTCAAGCCGGAGCACTACCGCTACTAA

>*Ll*SAHH (codon-optimised for *E. coli*)

ATGGCCCTGCTGGTTGAAAAAACCACCAGTGGTCGTGAATATAAAGTGAAAGATATGAGCCAGGCAGATTTTGGTCGTCT
GGAAATTGAACTGGCCGAAGTTGAAATGCCTGGTCTGATGGCAAGCCGTAGCGAATTTGGTCCGAGCCAGCCGTTTAAA
GGTGCAAAAATTACCGGTAGCCTGCACATGACCATTCAGACCGCAGTTCTGATTGAAACCCTGACCGCACTGGGTGCCGA
AGTTCGTTGGTGTAGCTGTAACATTTTTAGCACCCAGGATCATGCAGCAGCAGCAATTGCACGTGATAGCGCAGCAGTTTT
TGCATGGAAAGGCGAAACCCTGCAAGAATATTGGTGGTGTACCGAACGTGCACTGGATTGGGGTCCTGGTGGTGGTCCG
GATCTGATTGTTGATGATGGTGGTGATACCACACTGCTGATTCATGAAGGTGTTAAAGCCGAAGAGATCTATGAAAAAAG
CGGTCAGTTTCCGGATCCTGATAGCACCGATAATGCAGAATTCAAAATTGTGCTGAGCATCATCAAAGAAGGCCTGAAAA
CCGATCCGAAACGCTATCACAAAATGAAAGATCGTGTTGTTGGTGTGAGCGAAGAAACCACCACCGGTGTTAAACGTCTG
TATCAGATGCAGGCAAATGGCACCCTGCTGTTTCCGGCAATTAATGTTAATGATAGCGTGACCAAAAGCAAATTTGATAAC
CTGTATGGTTGTCGTCATAGCCTGCCGGATGGCCTGATGCGTGCAACCGATGTTATGATTGCAGGTAAAGTTGCAGTTGTT
GCAGGTTATGGTGATGTTGGTAAAGGTTGTGCAGCAGCCCTGAAACAGGCAGGCGCACGTGTTATTGTTACCGAAATTG
ATCCGATTTGTGCACTGCAGGCAACCATGGAAGGTCTGCAGGTTCTGACCCTGGAAGATGTTGTTAGCGAAGCAGATATT
TTTGTTACCACCACGGGCAACAAAGATATCATTATGCTGGACCACATGAAAAAAATGAAAAACAACGCCATCGTGTGCAA
CATCGGCCATTTTGATAATGAGATTGATATGCTGGGCTTAGAAACCCATCCGGGTGTGAAACGTATTACCATTAAACCGCA
GACCGATCGTTGGGTTTTTCCTGAAACCAATACCGGCATTATTATCCTGGCGGAAGGTCGTCTGATGAATCTGGGTTGTGC
AACCGGTCATCCGAGCTTTGTTATGAGCTGTAGCTTTACCAATCAGGTTATTGCACAGCTGGAACTGTGGAATGAAAAATC
AAGCGGCAAATACGAGAAAAAGGTTTATGTTCTGCCGAAACACCTGGATGAAAAAGTTGCCGCACTGCATCTGGAAAAA
CTGGGTGCAAAACTGACCAAACTGAGCAAAGATCAGGCCGATTATATCAGCGTTCCGGTTGAAGGTCCGTATAAACCGTT
TCATTATCGCTACTAA

>*Mc*SAHH (codon-optimised for *E. coli*)

ATGGTTAAGGATATCAAGATGGCCCAGCAGGGCGAAAGCCGTATTGAATGGGCACGTCGCCACATGCCGGTTCTGAATT
ATATTAAAGCCGAATATGAGAAGACCAAACCGCTGAAAGGTGTTAATGTGATTGCCTGCCTGCATGTGACCGTGGAAACC
GCAAATCTGATTAGTACCCTGCAGGCAGGTGGCGCCAATGTGGCACTGACCGCCAGCAATCCGCTGAGTACCCAGGATG
ATGTTGCAGCAGCACTGGCCAAACGTGGTGTGAAAGTGTATGCAATTCGCGGTGAAGATGAAAAACAGTATTATGAAAA
CATCGAGAGTGCCCTGAAAATTGGTCCGAATGTGACCCTGGATGATGGCGCCGATGTGATTGCAACCGTGCATAGTAAAC
ATCCGGAATATCTGAAAGGTATCTATGGCGGCTGTGAAGAAACCACCACCGGCGTGATTCGCCTGCGTGCCATGGCCAAA
GATGGCGCACTGAAATATCCGGTTATTGCCGTTAATGATGCCGAAACCAAAATGATGTTCGATAATCGCTATGGTACCGG
CCAGAGTACCCTGCATGGTATTATGAATGCCACCAATGTGCTGCTGGCAGGCAAAACCGTGGTGATTGTGGGTTATGGTT
GGTGCGGTCGCGGTGTTGCCATGCGCGCACGCGGTATGGGTAGCAATGTTATTATTGTGGAAGTGCATCCGCGCAAAGC
ACTGGAAGCCGTGATGGATGGTTATCGTGTTATGGATATGGAACAGGCCGCCCGCGAAGGTGATATCTTCATTACCGTGA
CCGGCGATATTAGCGTGATTCGCAAAGAACACTTCAAACTGATGAAAGATCAGGCAATTGTGTGTAATAGCGGCCACTTC
AATGTTGAAATTGATCTGAAAGATCTGGGCGCCATGGCCAAGAATGTGCGTGAAGTGAAAGCAGATGTTATGGAATATG
AAATGAAGGATGGCCGTCGTATCTATCTGCTGGGTGAAGGCCGCCTGGTGAATCTGGCCTGTGCCTTCGGCCATCCGCCG
GAAGTTATGGATATGAGCTTCGCAAATCAGGCACTGTGCGTTAAATATATTGTTGAAAATCACAAGAAGCTGAAGAATGA

AGTGTATCGTGTTCCGGAAGATATTGATAATCGTGTTGCACGTATGAAACTGGAAACCATGGGCATTAAACTGGAAAAAC
TGACCAAAGAACAGGAAAAATATCTGAGTAGCTGGAGTATGGGCACCTAA

>*Me*SAHH (codon-optimised for *E. coli*)

ATGACCAATAACGATTACAAGATCAAGGATATTGGCCTGGCCGAACTGGGTCGCAAACAGATTAATATGGCAGAAAAAG
AAATGCCGGGCCTGATTGCAACCCGCGAAAAATATGGTCCGCAGAAACCGCTGAAAGGTGCACGCGTTAGCGGCAGTCT
GCACATGACCGTGCAGACCGCAGTTCTGATTGAAACCTTAGTTGAACTGGGCGCCGATGTTCGCTGGGCCAGTTGCAATA
TCTTCAGTACCCAGGATGAAGCAGCAGCAGCCATTGCAGCCGAAGGCATTCCGGTGTTCGCATGGAAAGGCGAAACCGT
GGATGAATATTGGTGGTGCACCAAACAGGCACTGACCTGGAGTGATGGCAAAGGCCCGCATCTGATTGTGGATGATGGT
GGCGATGCCACCCTGATGATGCATCGTGGCTATGAAGCCGAAGATAATCCGAGCATTCTGGATGAACCGACCGAAAATA
AAGATCTGATGGCACAGAATAAAATCCTGAAAGATAGCCAGGCAGAAGATCCGCAGTTCTGGCATAAAGCAGTTGCAGA
ATGGAAAGGTGTGAGCGAAGAAACCACCACCGGCGTGCATCGTCTGTATCATTGGGAAAAAGATGGCGAACTGCTGGTT
CCGGCCATTAATGTGAATGATAGTGTGACCAAAAGTAAATTCGATAATATCTACGGTTGCCGCGAAAGCCTGGTGGATGC
CATTAAACGTGGCACCGATGTTATGGTTGCAGGTAAAGTGGCCGTGGTGTGCGGCTATGGCGATGTGGGTAAAGGTAGT
GCAGAAGCACTGGCCAATCATAAAGCCCGCGTTATTGTGACCGAAAGTGATCCGATCTGTGCCCTGCAGGCACTGATGGA
AGGTTATAGCGTTATGACCGTTGAAGATGCACTGCCGTATGGCGATATCTATGTTACCACCACCGGTAATCGTGATGTTAT
TACCACCGATCACATGAGTAAAATGAAAGATCAGGCAATTGTGTGCAATATTGGTCACTTCGATAATGAAATTCAGGTTG
ATGAACTGAATAAGATGAATAATGTGGAAAAGGTGGAAATTAAGCCGCAGGTTGATAAATATGAATTCCCGGATGGTCA
TAGTATCTATATGCTGGCCGAAGGTCGCCTGGTGAATCTGGGTCTGGCAACCGGCCATCCGAGCTTCGTTATGAGTAATA
GCTTCACCAATCAGACCCTGGCACAGATTGATCTGTGGGAAAATCCGAAAGATATTGGCGTGTATCGTCTGCCGAAAGTG
CTGGATGAAGAAGTTGCACGTCTGCATCTGGATAAACTGGGCGCAAAACTGACCAAAATGACCCAGGAACAGGCAGAAT
ATATTGGCTTCCCGGTGGATGGTCCGCATAAACCGGATCATTATCGTTATTAA

>*Mh*SAHH (codon-optimised for *E. coli*)

ATGGTGACCAATAAAGATTACAAGGTTAAAGATATCGGCCTGGCCGATAGCGGTCGTAAACAGCTGAATATTGCCGAACA
GGAAATGCCGGGTCTGATGGCCACCCGCGAAAAATATGGCCCGATGAAACCGCTGAAAGGCGCCCGTATTAGCGGTAGC
CTGCACATGACCGTGCAGACCGCAGTGCTGATTGAAACCTTAGTTGAACTGGGCGCCGATGTTCGCTGGGCCAGTTGTAA
TATCTTCAGTACCCAGGATGAAGCAGCAGCAGCCATTGCCGATACCGGTGTTCCGGTGTTCGCATGGAAAGGTGAAACCT
TAGATGATTATTGGTGGTGCACCAAACAGGCACTGACCTGGCCGGATGGTAAAGGCCCGAATCTGATTGTGGATGATGG
TGGTGATGCCACCCTGATGATGCATCGCGGTTATGCCGCCGAAGATGATCCGAGCATTCTGGATGAACCGACCGAAAATA
AAGAACTGATTGCCCAGAATGAAGTGCTGAAAAAAAGTCTGAAAGAAGATCCGCAGTTCTGGCATAAAGCCGTGGCAGA
TTGGAAAGGCGTGAGCGAAGAAACCACCACCGGCGTTCATCGTCTGTATCATTGGGAACGCAAAGGTGAACTGCTGACC
CCGGCAATTAATGTTAATGATAGTGTGACCAAAAGCAAATTCGATAATGTGTATGGCTGCCGTGAAAGCCTGGTGGATGC
CATTAAACGTGGCACCGATGTGATGATTGCAGGTAAAGTGGCCGTGGTGTGTGGTTATGGTGATGTGGGCAAAGGTAGT
GCCGCCGCACTGGCAAATCATAAAGCCCGCGTGATTATTACCGAAACCGATCCGATCTGTGCACTGCAGGCCCTGATGGA
AGGCTATGATGTTATGACCGTTGAAGATGCACTGCCGTATGGCGATATCTATGTTACCACCACCGGTAATTGTGATGTGCT
GACCACCGAACACATGAGTAATATGAAAGATCAGGCAATTGTGTGTAATATTGGTCACTTCGATAATGAAATCCAGGTTG
ATGCACTGAATAAAATGGATAATGTTAAGAAGGTGAACATCAAACCGCAGGTGGATGAATATCAGTTCCCGGATGGCCAT
AGTATCTATGTGCTGGCAGAAGGCCGTCTGGTGAATCTGGGTCTGGCCACCGGTCATCCGAGCTTCGTGATGAGCAATAG
CTTCACCAATCAGACCCTGGCACAGATTGATCTGTGGGAAAGCCCGAAAGAAGTTGGTGTGTATCGTCTGAGTAAAGTGC
TGGATGAAGAAGTTGCCCGCCTGCATCTGGAAAAACTGGGTGCAAAACTGACCAAAATGAGTCAGGAACAGGCAGAATA
TATTGGCTTCCCGGTTGAAGGTCCGTATAAACCGGAACATTATCGTTATTAA

>*Mi*SAHH (codon-optimised for *E. coli*)

ATGTACAAGGTTAAGGATATCAACCTGTGGAAAGAAGGTGAACGTAAAATTGAATGGGCCAAAGAACACATGCCGGTGC
TGAGTCTGATTCGTGAACGCTTCAAAGAAGAAAAACCGTTCAAAGGTCTGACCATTGGTATGGCCCTGCATCTGGAAGCA
AAAACCGCAGTTCTGGCCGAAACCTTAATGGAAGGCGGCGCAAAAATTGCCATTACCGGCTGCAATCCGCTGAGTACCCA
GGATGATGTTGCAGCAGCCTGCGCCAAAAAAGGCATGCATGTGTATGCATGGCGCGGCGAAACCAGAGAAGAATATTAT

GAAAATCTGAACCGCGTTCTGGATCATGAACCGGATATTATTATTGATGATGGCTGCGATCTGATCTTCCTGGTTCATACC
AAACGTACCGAACTGATTGAAAAAATTCGCGGTGCATGTGAAGAAACCACCACCGGTATTATTCGTCTGAAAGCAATGGA
AAAAGAAGGTGCACTGAAATTCCCGGTTATGGATGTGAATGATGCATATACCAAATATCTGTTCGATAATCGTTACGGTAC
CGGTCAGAGTGCCCTGGATGGTATTATTCGCGCAACCAATCTGCTGATTGCCGGCAAAACCGTTGTTGTGGCAGGTTATG
GTTGGTGCGGCCGCGGCGTGGCAATGCGTGCAAAAGGTCTGGGTGCCGAAGTTATTGTTACCGAAGTGAATCCGATTCG
CGCACTGGAAGCACGCATGGATGGCTTCCGCGTTATGAAAATGGAAGAAGCCGTTAAATATGGTGATATCTTCATTACCA
CCACCGGCTGTAAAGATGTTATTCGTAAAGAACACATGGTTAAAATGAAGGATAAGGCCATTCTGGCAAATGCCGGTCAC
TTCGATAATGAAATTAATAAACGTGACCTGGAAGAACTGGCCGAAAAAATTAAAGAAGTGCGTAATGGTGTTCGTGAATA
TAAACTGCGCGATGGTAAAAAACTGTATCTGCTGGGCGATGGCCGCCTGGTTAATCTGGTGTGTGCAGATGGCCATCCGT
GTGAAGTTATGGATATGAGCTTCAGCAATCAGGCACTGGCCGCAGAATATATTAAAGAACATTATAAGGAGCTGGAGCC
GCGTGTGTATCGTATTCCGTATGAACAGGATCTGATGATTGCCAAACTGAAACTGCGCAGCATGGGCATTGAAATTGATG
AACTGACCGAAGAACAGAAAAAATATCTGGAAGATTGGCGTGAAGGTACCTAA

>*Mj*SIHH (codon-optimised for *E. coli*)

ATGTACGAGGTGCGCGATATCAACCTGTGGAAAGAAGGTGAACGTAAAATTCAGTGGGCAAAACAGCACATGCCGGTTC
TGAATCTGATTCGTGAACGTTTCAAAGAAGAGAAACCGTTTAAAGGCATTACCATTGGTATGGCACTGCATCTGGAAGCA
AAAACCGCAGTTCTGGCAGAAACCCTGATGGAAGGTGGTGCAGAAATTGCCATTACCGGTTGTAATCCGCTGAGCACCCA
GGATGATGTTGCAGCAGCATGTGCAAAAAAAGGTATGCATGTTTATGCATGGCGTGGTGAAACCGTGGAAGAATATTAT
GAAAACCTGAACAAAGTGCTGGATCACAAACCGGATATTGTGATTGATGATGGTTGCGATCTGATCTTTCTGCTGCATACC
AAACGTACCGAACTGCTGGATAACATTATGGGTGGTTGTGAAGAAACCACCACCGGTATTATTCGTCTGAAAGCAATGGA
AAAAGAAGGCGCACTGAAATTTCCGGTTATGGATGTTAATGATGCCTACACCAAACACCTGTTTGATAATCGTTATGGCAC
CGGTCAGAGCGCACTGGATGGTATTCTGCGTGCAACCAATCTGCTGATTGCAGGTAAAACCGTTGTTGTTGCAGGTTATG
GTTGGTGTGGTCGTGGTGTTGCAATGCGTGCAAAAGGTCTGGGTGCAGAAGTTGTTGTTACCGAAGTTAATCCGATTCGT
GCACTGGAAGCCCGTATGGATGGTTTTCGTGTTATGAAAATGGAAAAGGCAGCCGAAATTGGCGATATCTTTATTACAAC
CACCGGTTGCAAAGATGTGATCCGCAAAGAACATATTCTGAAAATGCGTAATGGTGCCATTCTGGCAAATGCAGGTCATT
TTGATAACGAGATCAACAAGAAACACCTGGAAGAACTGGCCAAAAGCATTAAAGAAGTTCGTAATTGCGTCACCGAATAT
GATCTGGGCAACAAAAAAATCTATCTGCTTGGTGAAGGTCGTCTGGTTAATCTGGCATGTGCAGATGGTCATCCGTGTGA
AGTTATGGATATGAGCTTTGCAAATCAGGCACTGGCAGCAGAATATATCCTGAAAAATCACGAAAAACTGGAACCGCGTG
TTTATAACATTCCGTATGAACAGGATCTGATGATTGCCAGCCTGAAACTGAAAGCCATGGGTATTGAAATTGATGAGCTG
ACCAAAGAGCAGAAGAAGTATCTGGAAGATTGGCGTGAAGGCACCTAA

>*Mma*SAHH (codon-optimised for *E. coli*)

ATGAGCAACGTGAAAGATATGAGCCTGGCACCGAGCGGTCATCTGAAAATGGAATGGGCAAAACGTCACATGCCGGTTC
TGTGTCGTATTGCCGAAGAATTCAAAAACGATAAACCGTTTGAAGGTCTGACCATTGGTATGGCACTGCATCTGGAAGCA
AAAACCGCAATTCTGGCAGAAACCCTGCTGGAAGGTGGTGCAAAAATTGTTATTACCGGTTGTAATCCGCTGAGCACCCA
GGATGATGTTGCAGCAGCATGTGTTGAAAAAGGTATGGAAGTTTATGCATGGCGTGGTGAAACCAACGAAGAATATTAT
GAGAACCTGAACAAAGTGCTGGATAGCAATCCGGATATCATTATTGATGATGGTGCCGATCTGATCTTTCTGATTCATACC
GAACGTACCGAACTGATTGGCAAAATTATGGGTGGTTGTGAAGAAACCACCACCGGTATTATTCGTCTGAAAAGCATGGC
CGAAGAAGGTGCACTGAAATTTCCGGTTGTTAATGTGAATGATGCCTACACCAAACACCTGTTTGATAATCGTTATGGCAC
CGGTCAGAGCGCAATGGATGGCATTATTCGTACCACCAATCTGCTGATTGCAGGTAAAAATGTTGTGGTTGGTGGTTATG
GTTGGTGTGGTCGTGGTGTTGCAAGCCGTGCAGCAGGTCATGGTGCAAATGTGATTATTACCGAAGTTAATCCGATTCGT
GCACTGGAAGCCAAGATGGATGGTTTTACCGTGTTAAAAATGGAAGAGGCAGCCAAAATTGGCGATATTTTTGTTACCAC
CACAGGCTGCAAAGATATTCTGCGTATGGAACATTTTCTGCTGATGAAAGATGGTGCAGTTCTGAGCAATGCAGGCCATT
TTGATAACGAGATTAACAAAAACGACCTGAAAGAGCTGAGCAAGAGCGTTAAAGAAGCACGCTTTAACATCGAAGAGTA
TGATCTGGGCAACAAAAAGATTTATCTGCTTGGTGAAGGTCGTCTGGTTAATCTGGCATGTGCAGATGGTCATCCGTGTG
AAGTTATGGATATGAGCTTTGCAAATCAGGCACTGAGCGCCAAATTCATCAAAGAAAATAAAGGCAAGCTGGAAAACGA

GGTGTATGAAATTCCGTATGAGCAGGATTTCAAAATCGCACTGCTGAAACTGCATAGCATGGGTGCAGATATTGATGAAC
TGAGTCCGGAACAGCGTAAATATCTGAGCGATTGGAAAGAAGGCACCTAA

>*Mm*SAHH (codon-optimised for *E. coli*)

ATGAGCGACAAACTGCCGTATAAAGTTGCAGATATTGGTCTGGCAGCATGGGGTCGTAAAGCACTGGATATTGCAGAAA
ATGAAATGCCTGGTCTGATGCGTATGCGTGAAATGTATAGCGCAAGCAAACCGCTGAAAGGTGCACGTATTGCAGGTTGT
CTGCACATGACCGTTGAAACCGCAGTTCTGATTGAAACCCTGGTTGCACTGGGTGCAGAAGTTCGTTGGAGCAGCTGTAA
CATTTTTAGCACCCAGGATCATGCAGCAGCAGCAATTGCAAAAGCAGGTATTCCGGTTTTTGCATGGAAAGGTGAAACCG
ATGAAGAATATCTGTGGTGTATTGAACAGACCCTGCACTTTAAAGATGGTCCGCTGAATATGATTCTGGATGATGGTGGT
GATCTGACCAATCTGATTCATACCAAATATCCGCAGCTGCTGAGCGGTATTCGTGGTATTAGCGAAGAAACCACCACCGGT
GTTCATAACCTGTATAAAATGATGAGCAACGGCATTCTGAAAGTTCCGGCAATTAATGTTAATGATAGCGTGACCAAAAG
CAAATTCGATAATCTGTATGGTTGTCGCGAAAGCCTGATTGATGGTATTAAACGTGCAACCGATGTTATGATTGCAGGTAA
AGTTGCCGTTGTTGCAGGTTATGGTGATGTTGGTAAAGGTTGTGCACAGGCACTGCGTGGTTTTGGTGCACGTGTGATTA
TTACCGAAATTGATCCGATTAATGCACTGCAGGCAGCAATGGAAGGCTATGAAGTTACCACCATGGATGAAGCATGTAAA
GAAGGCAACATTTTTGTTACAACCACCGGTTGCGTTGATATCATTCTGGGTCGTCATTTTGAGCAGATGAAAGATGATGCC
ATTGTGTGCAATATCGGCCATTTTGATGTTGAGATTGATGTGAAATGGCTGAATGAAAACGCCGTGGAAAAAGTGAACAT
TAAACCGCAGGTTGATCGCTATTGGCTGAAAAATGGTCGTCGTATTATTCTGCTGGCAGAAGGTCGTCTGGTTAATCTGG
GTTGTGCAATGGGTCATCCGAGCTTTGTTATGAGCAATAGCTTTACCAATCAGGTGATGGCACAGATTGAACTGTGGACC
CATCCGGATAAATATCCGGTTGGTGTTCATTTCCTGCCGAAAAAACTGGATGAGGCAGTTGCAGAAGCACATCTGGGTAA
ACTGAATGTGAAACTGACCAAACTGACAGAAAAACAGGCACAGTATCTGGGTATGCCGATTAACGGTCCGTTTAAACCGG
ATCATTATCGCTATTAA

>*Mt*SAHH (codon-optimised for *E. coli*)

ATGGAAAAGAGTATCGTTAAGGATATCACCCTGGCCGATGCCGGCGCACGCCGTATTGAATGGGCCCGCCGCCACATGCC
GGTTCTGCAGCTGATTAAAGAAGAATTCGAACGTGAACGCCCGCTGGAAGGCATTCGTATTGTTGCATGTCTGCATGTGA
CCGTTGAAACCGCAAATCTGATTGAAGCCCTGCGTGCAGGCGGCGCCGAAATTGCACTGACCGGCAGCAATCCGCTGAG
CACCCAGGATGATGTGGCAGCAGCACTGGCCAGTCGTGGCATTCATGTGTATGCATTCCGTGGCGAAAATGAAAGCGAA
TATTATGAATGCATCGAACGCGCCCTGGAACTGCGCCCGAATGTTACCCTGGATGATGGTGCAGATACCATTGCAGTTGT
GCATAAAAGCCATCGCGAACTGATTCCGGAAATTCTGGGTGGCTGCGAAGAAACCACCACCGGTGTGATGCGCCTGCGT
GCAATGGCAGATGATGGCGCACTGGCATATCCGGTGATTGCAGTGAATGATGCAGAAACCAAAATGATGTTCGATAATC
GCTATGGCACCGGTCAGAGTACCCTGCATGGTATTATGAATGCCACCAACTTCCTGTTCGCCGGCAAAACCGTGGTTGTG
GCCGGCTATGGCTGGTGTGGCCGCGGCTTCGCCATGCGTGCAAAAGGTCTGGGCGCAAATGTGATTGTGGTTGAAACCG
AACCGCGTAAAGCCCTGGAAGCAGCAATGGATGGTTATCGCGTGATGAGTATGCGCAGTGCAGCCCCGCTGGGTGATCT
GTTCGTGACCCTGACCGGTGATATTAATGTGATTCGTAATGAACACTTCGAACTGATGAAAGATCAGGTGATTCTGGCAA
ATAGTGGCCACTTCAATGTTGAAATTGATATTCCGGGCCTGGAAAGTATGGCCGTTAGTACCGATGAAATTCAGCCGAAT
GTGAAAGAATATGTGATGCCGGATGGCCGTCGTATCTATCTGCTGGCAGAAGGTCGCCTGATTAATCTGGCCGCCGCCTA
TGGTCATCCGCCGGAAGTGATGGATATGAGCTTCGCCAATCAGGCCCTGAGTGTTCGTTATATTGTTGAAAATGGTCGTC
GTCTGGAACGCAAAGTGTATCCGGTGCCGGTGGAAATTGATCGTCGCGTTGCCGAACTGAAACTGAAAGCCATGGGTAT
TGAAATTGAACGTCTGACCCCGGAACAGGATCGTTATCTGCATAGTTGGCAGATGGGCACCTAA

>*Pa*SAHH (codon-optimised for *E. coli*)

ATGAGCGCAGTTATGACACCGGCAGGTTTTACCGATTATAAAGTTGCAGATATTACCCTGGCAGCATGGGGTCGTCGTGA
ACTGATTATTGCAGAAAGCGAAATGCCTGCACTGATGGGTCTGCGTCGTAAATATGCAGGTCAGCAGCCGCTGAAAGGT
GCAAAAATTCTGGGTTGTATTCACATGACCATTCAGACCGGTGTTCTGATTGAAACCCTGGTTGCACTGGGTGCAGAAGTT
CGTTGGAGCAGCTGTAACATTTTTAGCACCCAGGATCAGGCAGCAGCAGCAATTGCAGCAGCCGGTATTCCGGTTTTTGC
ATGGAAAGGTGAAACCGAAGAGGAATATGAATGGTGTATTGAACAGACCATTCTGAAAGATGGTCAGCCGTGGGATGCA
AATATGGTTCTGGATGATGGTGGTGATCTGACCGAAATTCTGCATAAAAAGTATCCGCAGATGCTGGAACGTATTCATGG
TATTACCGAAGAAACCACCACCGGTGTGCATCGTCTGCTGGATATGCTGAAAAATGGCACCCTGAAAGTTCCGGCAATTA

ATGTTAATGATAGCGTGACCAAAAGCAAAAACGACAACAAATATGGCTGTCGCCATAGCCTGAATGATGCAATTAAACGT
GGCACCGATCATCTGCTGAGCGGTAAACAGGCACTGGTTATTGGTTATGGTGATGTTGGTAAAGGTAGCAGCCAGAGCC
TGCGTCAAGAAGGTATGATTGTTAAAGTTGCCGAAGTTGATCCGATTTGTGCAATGCAGGCATGTATGGATGGTTTTGAA
GTTGTTAGCCCGTATAAAAACGGCATTAATGATGGCACCGAAGCAAGCATTGATGCAGCACTGCTGGGTAAAATTGATCT
GATTGTTACCACCACGGGTAATGTGAATGTTTGTGATGCAAACATGCTGAAAGCCCTGAAAAAACGTGCAGTTGTTTGCA
ACATTGGCCACTTTGATAACGAAATTGATACCGCCTTTATGCGCAAAAATTGGGCATGGGAAGAAGTTAAACCGCAGGTT
CATAAAATTCATCGTACCGGTAAAGATGGCTTTGATGCCCATAATGATGATTATCTGATTCTGCTGGCAGAAGGTCGTCTG
GTTAATCTGGGTAATGCAACCGGTCATCCGAGCCGTATTATGGATGGCAGCTTTGCAAATCAGGTGCTGGCACAGATTCA
CCTGTTTGAACAGAAATATGCCGATCTGCCTGCAGCAGAAAAAGCCAAACGTCTGAGCGTTGAAGTTCTGCCGAAAAAAC
TGGATGAAGAGGTTGCCCTGGAAATGGTTAAAGGTTTTGGTGGTGTTGTTACCCAGCTGACCCCGAAACAGGCAGAATAT
ATCGGTGTTAGCGTGGAAGGTCCGTTTAAACCGGATACCTATCGCTATTAA

>*Pfu*SAHH (codon-optimised for *E. coli*)

ATGGATTGCGGCAAAGATTATTGCGTTAAAGATCTGAGCCTGGCAGAAGAAGGTTGGAAAAAAATCGATTGGGTTAGCC
GTTTTATGCCGGTTCTGCAGTATATCAAACGCGAATTCGAAGAGAAAAAACCGTTTAAAGGTGTTCGTATTGCAGCAACCC
TGCATCTGGAAATGAAAACCGCATTTCTGCTGCTGACCCTGAAAGCCGGTGGTGCAGAAGTTAGCGCAGCAGCAAGCAAT
CCGCTGAGCACCCAGGATGATGTTGTTGCAGCACTGGCAAAAGCGGGTGTTAAAGTTTATGCAATTCGTGGTGAAAGCC
GTGAGCAGTATTATGAGTTCATGCATAAAGCACTGGATATCCGTCCGAACATCATTATTGATGATGGTGCAGATATGATCA
GCCTGGTTCATAAAGAACGTCAAGAAATGCTGGATGAAATTTGGGGTGGTAGCGAAGAAACCACCACCGGTGTTATTCGT
CTGCGTGCAATGGAAAAAGCAGGCATTCTGAAATTTCCGGTTATTGCCGTTAACGACAGCTACATGAAATACCTGTTTGAT
AATCGTTATGGCACCGGTCAGAGCACCTGGGATGGTATTATGCGTGCAACCAATCTGCTGATTGCAGGTAAAAATGTTGT
GGTGGTTGGTTATGGTTGGTGTGGTCGTGGTATTGCAATGCGTGCACGTGGTCTGGGTGCAACCGTTATTGTTGTTGAAG
TTGATCCGATTAAAGCCCTGGAAGCACGTATGGATGGTTTTCTGGTTATGGATATGAAAGAGGCAGCAAAAATCGGCGAT
ATTTTTGTTACCGCAACCGGCAACATTAAATGCATTCGTCGTGAACATTTCGAGCTGATGAAAGATGGTGCAATTATGGCA
AATGCCGGTCATTTTGATGTGGAAATTTGGAAACCGGATCTGGAAAAACTGGCCGTGGAAATCAATAATCCGCGTCCGAA
TGTTACCGAGTATAAACTGAAAGACGGTCGTCGTCTGTATCTGCTGGCAGATGGTCGTCTGGTTAATCTGGTTGCAGCCG
ATGGTCATCCGGCAGAAATCATGGATATGTCATTTGCACTGCAGGCCAAAGCAGCCGAATATATCAAAGATAATCATGAA
CGTCTGGAACCGAAGGTGTATATTCTGCCTCGTGAAATTGATGAAATGGTGGCACGTATTAAACTGGAAAGCATGGGCAT
TAAAATCGAAGAACTGACCGAAGAACAGAAAAAGTATCTGGAAAGTTGGGAACATGGCACCTAA

>*Sac*SAHH (codon-optimised for *E. coli*)

ATGGATTATCGCGTTAAAGATCTGAGCCTGGCAGAACAGGGTCGTAAACAAATTGAATGGGCAGAACTGCACATGCCTG
CACTGATGGAAATTCGTAAACGTTTTAATGCAGAGAAACCGCTGGATGGTATTCGTATTGGTGCCGTTCTGCATGTTACCA
AAGAAACCGCAGTTCTGGTTGAAACCCTGAAAGCCGGTGGTGCAGAAATTGCACTGGCAGGTAGCAATCCGCTGAGCAC
CCAGGATGATGTTGCAGCAGGTCTGGCAAAAAATGGTATTCATGTTTATGCATGGCGTGGCGAAACCGAGAAAGATTATT
ATGATAACATTCGCGAAATCCTGAAATATGAACCGCATGTGATTATGGATGATGGTGGTGATCTGCATGCCTATGTGCAT
GAAAATAATCTGACCAGCAAAATTGTTGGTGGCACCGAAGAAACCACCACCGGTGTTATTCGTCTGAAAGCCATGGAAGA
AGAGAAAGTTCTGAAATATCCGGTGATTGCCGTGAATAATGCCTTTACCAAATACCTGTTTGATAACCGTATTGGCACCGG
TCAGAGCACCATTGATGGCATTCTGCGTGCAACCAATATTCTGATTGCAGGTAAAGTTGCCGTGGTGATTGGTTATGGTTG
GGTTGGTCGTGGTATTGCAAGCCGTTTTAAAGGTATGGGTGCACGTGTTATTGTTGTTGAAAGCAGCCCGTTTCGTGCACT
GGAAGCCCTGATGGATGGTTTTGATGTTATGACCATGAATCGTGCAAGCGAAATTGGCGATATTTTTGTTACCGCAACCG
GTAATCTGAATGTTGTTAGCCGTGATCATATTCTGCGTATGAAAGATGGTGCGGTTCTGGCAAATAGCGGTCACTTTAATG
TTGAGATTGATGTGAAAGGCCTGAAAGAAATTAGCGTGGAAACCCGTGAAGTTCGTCAGAATCTGGAAGAATATAAACT
GCGTAATGGCAAACGCATTTATCTGCTGGCAGATGGTCGTCTGGTTAATCTGGTTGCAGCCGAAGGTCATCCGAGCGAAG
TTATGGATCTGAGCTTTTGTAATCAGGCACTGAGCGTTGAACATCTGATTAAAAACAAAGGCAAACTGGAAAACAAAGTG
TACAACGTGCCGATCGAAATTGATGAACAGGTTGCACGTCTGAAACTGAAAGCACTGGGTATTGAAATTGAAGAACTGAC
CATCGAGCAGAAAGAATACATCAAACAGTGGAAATACGGCACCTAA

>*Sa*SAHH

ATGGCAAGCGCCCAGCAGCACGACTTCAAGGTCGCCGACCTCTCCCTCGCGGAGTTCGGCCGCAAGGAGATCACCCTCGC
CGAGCACGAGATGCCCGGCCTGATGTCGATCCGCGAGGAGTACGCCGCGTCCCAGCCGCTGGCCGGCGCCCGCGTCACC
GGCTCGCTGCACATGACCGTCCAGACGGCCGTCCTCATCGAGACGCTCACCGCCCTGGGCGCGGAGGTCCGCTGGGCCTC
CTGCAACATCTTCTCCACCCAGGACCACGCCGCCGCCGCCATCGCGGTCGGCCCCAACGGCACCCCGGACAACCCGCAGG
GCGTCCCGGTCTTCGCCTGGAAGGGCGAGAGCCTGGAGGAGTACTGGTGGTGCACCGAGCAGGCGCTGACCTGGCCGA
ACACCCCCACCGGCGGCCCCAACATGATCCTGGACGACGGCGGTGACGCCACCCTCCTCGTCCACAACGGCGTCCAGTAC
GAGAAGGACGGCAAGGTCCCCGACCCGGTCACCGCCGAGTCCGACGAGCACCGCGTCATCCTCCAGCTGCTGACCCGCA
CCCTCGGCGAGAACCCGCAGAAGTGGACCCAGCTCGCCTCGGAGATCCGCGGCGTCACCGAGGAGACCACCACCGGCGT
CCACCGCCTCTACGAGATGCAGCGCGACGGCCAGCTGCTCTTCCCGGCGATCAACGTCAACGACGCGGTCACCAAGTCGA
AGTTCGACAACAAGTACGGCTGCCGCCACTCCCTGATCGACGGCATCAACCGTGCCACCGACGTCCTCATCGGCGGCAAG
ACCGCCGTCGTCTGCGGCTACGGCGACGTCGGCAAGGGCTGCGCCGAGTCCCTGCGCGGCCAGGGCGCCCGCGTCATCG
TCACCGAGATCGACCCGATCTGCGCCCTCCAGGCGGCGATGGACGGCTACCAGGTCGCCACCCTCGACGACGTGATCGG
CCAGGCCGACATCTTCGTCACCACGACCGGCAACAAGGACATCATCATGGCCGCCGACATGGCCAAGATGAAGCACCAG
GCCATCGTGGGGAACATCGGCCACTTCGACAACGAGATCGACATGGCCGGCCTCGCCGCCATCCCCGGCATCGTCAAGG
ACGAGGTCAAGCCGCAGGTCCACACCTGGACCTTCCCCGACGGCAAGGTCATCATCGTGCTGTCCGAGGGCCGCCTGCTC
AACCTGGGCAACGCCACCGGCCACCCGTCCTTCGTGATGTCCAACAGCTTCGCGGACCAGACGCTGGCCCAGATCGAGCT
GTTCACCAAGCCCGACGAGTACCCGACCGACGTCTACGTGCTGCCCAAGCACCTCGACGAGAAGGTCGCCCGCCTCCACC
TCGACGCCCTCGGCGTCAGGCTGACGACCCTCCGCCCGGAGCAGGCCGCGTACATCGGCGTCTCCGTCGAAGGCCCGTTC
AAGCCGGACCACTACCGGTACTGA

>*Sf*SAHH

ATGACGACGACCTCCACGACCGGCCATGACTTCAAGGTCGCGGACCTCTCTCTGGCCGCTTTCGGCCGCAAGGAGATCAC
GCTGGCCGAGCACGAGATGCCCGGCCTGATGGCGATCCGCAAGGAGTACTCCGCGGAGAAGCCGCTGGCCGGAGCGCG
CATCACGGGCTCCCTGCACATGACGGTGCAGACGGCCGTGCTCATCGAAACCCTCGTCGCCCTCGGCGCCGAGGTCCGCT
GGGCCTCCTGCAACATCTTCTCCACCCAGGACCACGCCGCCGCGGCCATCGCCGTCGGCCCCGACGGCACCCCGGACAAC
CCGCGGGGCGTCCCGGTCTTCGCCTGGAAGGGCGAGACCCTGGAGGAGTACTGGTGGTGCACGGAACAGGCCCTCACCT
GGCCGAACACGCCCACCGGCGGCCCCAACATGATTCTCGACGACGGTGGTGACGCCACCCTCCTCGTCCACAAGGGCGTC
GAGTACGAGAAGGCCGGTGCCGCCCCCTCGGTCGACACCGCCGAGAACGACGAGCACCGCGTCATCCTCCAGCTCCTCA
ACCGCACCCTCGCCGAGAGCCCGCAGAAGTGGACGCAGCCCGCGTCGGAGATCCGCGGCGTCACCGAGGAGACCACCAC
CGGCGTCCACCGCCTCTACGAGATGCAGCAGGCCGGCACGCTGCTCTTCCCGGCGATCAACGTCAACGACGCCGTCACCA
AGTCGAAGTTCGACAACAAGTACGGCTGCCGCCACTCCCTGATCGACGGCATCAACCGCGCCACCGACGTCCTCATCGGC
GGCAAGACCGCCGTCGTCTGCGGCTACGGCGATGTCGGCAAGGGCTGCGCCGAGTCCCTGCGCGGCCAGGGCGCCCGG
GTCATGATCACTGAGATCGACCCGATCTGCGCCCTCCAGGCGGCGATGGACGGCTACCAGGTGGTGCGGCTGGACGATG
TCGTCGAGACCGCCGACATCTTCATCACCACCACGGGCAACAAGGACATCATCATGGCCTCGGACATGGCCAAGATGAAG
CACCAGGCCATCGTCGGCAACATCGGCCACTTCGACAACGAGATCGACATGGCCGGTCTCGCCGCCATCGACGGCATCGT
CAAGGACGAGGTCAAGCCGCAGGTCCACACCTGGACCTGGCCGGACGGCAAGAGCATCATCGTCCTGTCCGAGGGCCGC
CTGCTGAACCTGGGCAACGCCACCGGGCACCCCTCGTTCGTGATGTCGAACAGCTTCGCGAACCAGACGATCGCCCAGAT
CGAACTGTTCACCAAGCCGGAGTCGTACCCGACCGACGTCTACGTGCTGCCCAAGCACCTCGACGAGAAGGTCGCCCGCC
TCCACCTCGACGCCCTCGGCGCCAAGCTGACCACGCTCCGCCCGGAGCAGGCGGCGTACATCGGCGTCCCGGTCGAGGG
TCCCTACAAGCCGGACCACTACCGCTACTGA

>*Sso*SAHH (codon-optimised for *E. coli*)

ATGAGCTACAAAATCAAAGATCTGAGCCTGGCAAGCGAAGGTAAAAAACAAATTGAATGGGCAGAACGTCACATGCCGA
CACTGATGGAAATTCGTAAACGTTTTAAAGCCGAGAAACCGCTGAAAGGCATTAACATTAGCGCAGTTCTGCATGTTACC
AAAGAAACCGCAGCACTGGTTAAAACCCTGAAAATTGGTGGTGCAAATGTTGCACTGGCAGGTAGCAATCCGCTGAGCA
CCCAGGATGATGTTGCAGCAGCCCTGGTTGAAGAAGGTATTAGCGTTTTTGCATGGAAAGGCGAAAATGAAACCGAGTA

TTACAGCAACATTGAGAGCATCGTGAAAATCCATGAACCGAACATTGTTATGGATGATGGTGCCGATCTGCATGCCTATAT
TCATGAAAAAGTTAGCAGCAAGCTGGATATTTATGGTGGCACCGAAGAAACCACCACCGGTGTTATTCGTCTGAAAGCAA
TGGAAAAAGATGGCGTTCTGAAATATCCGCTGGTTGCAGTTAATAACGCCTATACCAAATACCTGTTCGATAATCGTTATG
GCACCGGTCAGAGCGCAATTGATGGTATTCTGCGTGCAACCAATATTCTGATTGCAGGTAAAATTGCAGTGGTTGCAGGT
TATGGTTGGGTTGGTCGTGGTATTGCAAATCGTCTGCGTGGTATGGGTGCACGTGTTATTGTTACCGAAGTTGATCCGATT
CGTGCACTGGAAGCAGTGATGGATGGTTTTGATGTTATGCCGATTGCCGAAGCAAGCAAAGTTGGTGATATTTTTGTTAC
CGCAACCGGTAATACCAAAGCCATTCGTGTTGAACACATGCTGAATATGAAAGATGGTGCCATTCTGAGCAATGCCGGTC
ACTTTAATGTTGAAGTTGATGTGAAAGGCCTGAAAGAAACAGCAGTTAAAGTGCGTAATATTCGTCCGTATGTGGATGAA
TATACCCTGCCGAATGGTAAACGTGTTTATCTGCTGGCAGATGGTCGTCTGGTTAATCTGGCAGCAGCAGAAGGTCATCC
GAGCGAAGTTATGGATATGAGCTTTGCAAATCAGGCACTGGCCGTTGAATATCTGGTGAAAAATCGTGGTAAGCTGGAA
AAAAAGGTGTACAATATGCCGATGGAACTGGATTATGAAGTGGCACGTATTAAGCTGAAAAGCATGGGTATTCAGATTG
ATGAACTGACCGAAGAACAGAAAGAATACCTGGAACAGTGGAAAAGCGGCACCTAA

>*Tk*SAHH

ATGGACTGCACGAAGGATTACTGCGTTAAGGACATCTCCCTGGCACCGAGCGGGGAGAAGAAGATAGACTGGGTCTCCC
GCTTCATGCCGGTTCTCCAGCACATCAGGAAGGACTTTGAAGAGAGGAAACCGTTTAAGGGCGTTAGGATAGCAGCGAC
TCTACACCTTGAGATGAAGACTGCCTTTCTGCTTCTGACGCTGAAAGCGGCTGGAGCCGAGGTTTCGGCAGCTGCCAGCA
ACCCACTCTCCACCCAGGACGATGTAGTTGCCGCTCTGGCAAAGGCGGGGGTCAAGGTCTACGCTATAAGGGGAGAGGA
CAGGGAGCAGTACTACGAGTTCATGCACAAGGCCCTCGACGTAAAACCGAACATCATCATAGACGACGGAGCGGATATG
GTGAGCACGGTTTTGAAGGAGAGGCAGGAGCTGATTCCCGAAATATGGGGGGCAAGCGAGGAAACCACAACCGGCGTC
ATAAGGCTCCGTGCCATGGAGAAGGATGGCGTCCTCAAGTTCCCGATCATAGCGGTCAACGATTCCTACACCAAATACCT
CTTCGACAACCGCTATGGAACCGGTCAGTCCACCTGGGACGGCATCATAAGGACTACTAACCTCCTCGTCGCTGGAAAGA
ACGTCGTTGTTGTCGGCTATGGCTGGTGCGGCAGGGGCATAGCAATGCGCGCGAGGGGACTTGGAGCGACCGTTATCGT
CGTTGAGGTTGACCCAATAAGGGCTCTAGAAGCCAGAATGGACGGATTCCTCGTCATGGACATGATGGAGGCGGCGAAG
GTAGGGGACATCTTCATAACTGCCACCGGAGACATCAACTGCATAAGGAAGGAGCACTTCGAGCTCATGAAGGACGGAG
CTATTCTCGCCAACGCCGGCCACTTCGATGTCGAGATTTCAAAGCCTGACCTTGAGGCCCTCGCAGTTGAGATAAGCGAG
CCAAGGCCGAACATCACAGAGTACAAAATGGCAGACGGGAGGAGGCTCTACCTCCTGGCTGAGGGCAGGCTTGTGAATC
TAGCCGCTGCCGACGGTCATCCAGCGGAGATAATGGACATGAGCTTCGCGCTCCAGGCGAAAGCCGCTGAGTACATCAA
GGAGAACCGCGGAAGGCTTGAGCCGAAGGTCTACGTTCTTCCGAGGGAGATAGACGAGATGGTGGCGAGGATAAAGCT
CGCCTCGATGGGGATAAAAATTGAGGAACTCACAGAAGAGCAAAAGAAATATCTGGAAAGCTGGGAGCACGGCACCTG
A

>*Tm*SAHH (codon-optimised for *E. coli*)

ATGAACACCGGTGAGATGAAGATTAATTGGGTTAGCCGTTATATGCCGCTGCTGAACAAAATTGCCGAAGAATATAGCCG
TGAAAAACCGCTGAGTGGTTTTACCGTTGGTATGAGCATTCATCTGGAAGCAAAAACCGCATATCTGGCAATTACCCTGA
GCAAACTGGGTGCAAAAGTTGTTATTACCGGTAGCAATCCGCTGAGCACCCAGGATGATGTTGCAGAAGCACTGCGTAGC
AAAGGTATTACCGTTTATGCACGTCGTACCCATGATGAAAGCATTTATCGTGAAAACCTGATGAAAGTGCTGGATGAACG
TCCGGATTTCATTATTGATGATGGTGGTGATCTGACCGTTATTAGCCATACCGAACGTGAAGAAGTTCTGGAAAATCTGAA
AGGTGTTAGCGAAGAAACCACCACCGGTGTGCGTCGTCTGAAAGCACTGGAAGAAACCGGTAAACTGCGTGTTCCGGTT
ATTGCAGTTAATGACAGCAAAATGAAATACCTGTTCGATAATCGTTATGGCACCGGTCAGAGCACCTGGGATGCAATTAT
GCGTAATACCAATCTGCTGGTTGCCGGTAAAAATGTTGTTGTTGCAGGTTATGGTTGGTGTGGTCGTGGTATTGCACTGC
GTGCAGCAGGTCTGGGTGCACGTGTTATTGTTACCGAAGTTGATCCGGTTAAAGCAGTTGAAGCAATTATGGATGGCTTT
ACCGTTATGCCGATGAAAGAAGCAGTTAAAATTGCGGATTTTGTGATTACCGCAAGCGGCAATACCGATGTTCTGAGCAA
AGAAGATATTCTGAGCCTGAAAGATGGTGCAGTTCTGGCAAATGCAGGTCATTTTAATGTTGAAATTCCGGTTCGTGTGCT
GGAAGAGATTGCAGTTGAAAAATTTGAAGCACGTCCGAATGTTACCGGTTATACCCTGGAAAATGGTAAAACCGTTTTTC
TGCTGGCAGAAGGTCGTCTGGTTAATCTGGCAGCCGGTGATGGTCATCCGGTTGAAATCATGGATCTGAGCTTTGCACTG
CAGATTTTTGCCGTTCTGTATCTGCTGGAAAACCATCGTAAAATGAGCCCGAAAGTTTATATGCTGCCGGATGAAATTGAT

GAACGTGTTGCACGTATGAAACTGGATAGCCTGGGTGTTAAAATCGATGAACTGACCGAAAAACAGCGTCGTTATCTGCG
TAGCTGGCAGTAA

Supplementary Notes 2

Protein sequences of the enzymes used in this study. His_6-tags are underlined.

>*Sc*HSMT

MGSSHHHHHHSSGLVPRGSHMKRIPIKELIVEHPGKVLILDGGQGTELENRGININSPVWSAAPFTSESFWEPSSQERKVVEE
MYRDFMIAGANILMTITYQANFQSISENTSIKTLAAYKRFLDKIVSFTREFIGEERYLIGSIGPWAAHVSCEYTGDYGPHPENIDYY
GFFKPQLENFNQNRDIDLIGFETIPNFHELKAILSWDEDIISKPFYIGLSVDDNSLLRDGTTLEEISVHIKGLGNKINKNLLLMGVNC
VSFNQSALILKMLHEHLPGMPLLVYPNSGEIYNPKEKTWHRPTNKLDDWETTVKKFVDNGARIIGGCCRTSPKDIAEIASAVDK
YS

>*Mj*DadD

MGSSHHHHHHSSGLVPRGSHMILIKNVFVNGKRQDILIEGNKIKKIGEVKKEEIENAEIIDGKNKIAIPGLINTHTHIPMTLFRGVA
DDLPLMEWLNNYIWPMEAKLNEEIVYWGTLLGCIEMIRSGTTTFNDMYFFLEGIAKAVDESGMRAVLAYGMIDLFDEERRERE
LKNAEKYINYINSLNNSRIMPALGPHAPYTCSKELLMEVNNLAKKYNVPIHIHLNETLDEIKMVKEKTGMEPFIYLNSFGFFDDVR
AIAAHCVHLTDEEIKIMKQKNINVSHNPISNLKLASGVAPIPKLLAEGINVTLGTDGCGSNNNLNLFEEIKVSAILHKGVNLNPTVV
KAEEAFNFATKNGAKALNIKAGEIREGYLADIVLINLDKPYLYPKENIMSHLVYAFNGFVDDVIIDGNIVMRDGEILTVDEEKVYE
KAEEMYEILRS

>*Cg*SAHH

MGSSHHHHHHSSGLVPRGSHMAQVMDFKVADLSLAEAGRHQIRLAEYEMPGLMQLRKEFADEQPLKGARIAGSIHMTVQT
AVLIETLTALGAEVRWASCNIFSTQDEAAAAIVVGSGTVEEPAGVPVFAWKGESLEEYWWCINQIFSWGDELPNMILDDGGD
ATMAVIRGREYEQAGLVPPAEANDSDEYIAFLGMLREVLAAEPGKWGKIAEAVKGVTEETTTGVHRLYHFAEEGVLPFPAMNV
NDAVTKSKFDNKYGTRHSLIDGINRATDMLMGGKNVLVCGYGDVGKGCAEAFDGQGARVKVTEADPINALQALMDGYSVV
TVDEAIEDADIVITATGNKDIISFEQMLKMKDHALLGNIGHFDNEIDMHSLLHRDDVTRTTIKPQVDEFTFSTGRSIIVLSEGRLLN
LGNATGHPSFVMSNSFADQTIAQIELFQNEGQYENEVYRLPKVLDEKVARIHVEALGGQLTELTKEQAEYIGVDVAGPFKPEHY
RY

>*Ll*SAHH

MGSSHHHHHHSSGLVPRGSHMALLVEKTTSGREYKVKDMSQADFGRLEIELAEVEMPGLMASRSEFGPSQPFKGAKITGSLH
MTIQTAVLIETLTALGAEVRWCSCNIFSTQDHAAAAIARDSAAVFAWKGETLQEYWWCTERALDWGPGGGPDLIVDDGGDT
TLLIHEGVKAEEIYEKSGQFPDPDSTDNAEFKIVLSIIKEGLKTDPKRYHKMKDRVVGVSEETTTGVKRLYQMQANGTLLFPAINV
NDSVTKSKFDNLYGCRHSLPDGLMRATDVMIAGKVAVVAGYGDVGKGCAAALKQAGARVIVTEIDPICALQATMEGLQVLTL
EDVVSEADIFVTTTGNKDIIMLDHMKKMKNNAIVCNIGHFDNEIDMLGLETHPGVKRITIKPQTDRWVFPETNTGIIILAEGRL
MNLGCATGHPSFVMSCSFTNQVIAQLELWNEKSSGKYEKKVYVLPKHLDEKVAALHLEKLGAKLTKLSKDQADYISVPVEGPYK
PFHYRY

>*Mc*SAHH

MGSSHHHHHHSSGLVPRGSHMVKDIKMAQQGESRIEWARRHMPVLNYIKAEYEKTKPLKGVNVIACLHVTVETANLISTL
QAGGANVALTASNPLSTQDDVAAALAKRGVKVYAIRGEDEKQYYENIESALKIGPNVTLDDGADVIATVHSKHPEYLKGIYGGC
EETTTGVIRLRAMAKDGALKYPVIAVNDAETKMMFDNRYGTGQSTLHGIMNATNVLLAGKTVVIVGYGWCGRGVAMRARG
MGSNVIIVEVHPRKALEAVMDGYRVMDMEQAAREGDIFITVTGDISVIRKEHFKLMKDQAIVCNSGHFNVEIDLKDLGAMAK
NVREVKADVMEYEMKDGRRIYLLGEGRLVNLACAFGHPPEVMDMSFANQALCVKYIVENHKKLKNEVYRVPEDIDNRVARM
KLETMGIKLEKLTKEQEKYLSSWSMGT

>*Me*SAHH

MGSSHHHHHHSSGLVPRGSHMTNNDYKIKDIGLAELGRKQINMAEKEMPGLIATREKYGPQKPLKGARVSGSLHMTVQT
AVLIETLVELGADVRWASCNIFSTQDEAAAAIAAEGIPVFAWKGETVDEYWWCTKQALTWSDGKGPHLIVDDGGDATLMMH
RGYEAEDNPSILDEPTENKDLMAQNKILKDSQAEDPQFWHKAVAEWKGVSEETTTGVHRLYHWEKDGELLVPAINVNDSVTK
SKFDNIYGCRESLVDAIKRGTDVMVAGKVAVVCGYGDVGKGSAEALANHKARVIVTESDPICALQALMEGYSVMTVEDALPYG
DIYVTTTGNRDVITTDHMSKMKDQAIVCNIGHFDNEIQVDELNKMNNVEKVEIKPQVDKYEFPDGHSIYMLAEGRLVNLGLAT
GHPSFVMSNSFTNQTLAQIDLWENPKDIGVYRLPKVLDEEVARLHLDKLGAKLTKMTQEQAEYIGFPVDGPHKPDHYRY

>*Mh*SAHH

MGSSHHHHHHSSGLVPRGSHMVTNKDYKVKDIGLADSGRKQLNIAEQEMPGLMATREKYGPMKPLKGARISGSLHMTV
QTAVLIETLVELGADVRWASCNIFSTQDEAAAAIADTGVPVFAWKGETLDDYWWCTKQALTWPDGKGPNLIVDDGGDATL
MMHRGYAAEDDPSILDEPTENKELIAQNEVLKKSLKEDPQFWHKAVADWKGVSEETTTGVHRLYHWERKGELLTPAINVNDS
VTKSKFDNVYGCRESLVDAIKRGTDVMIAGKVAVVCGYGDVGKGSAAALANHKARVIITETDPICALQALMEGYDVMTVEDAL
PYGDIYVTTTGNCDVLTTEHMSNMKDQAIVCNIGHFDNEIQVDALNKMDNVKKVNIKPQVDEYQFPDGHSIYVLAEGRLVNL
GLATGHPSFVMSNSFTNQTLAQIDLWESPKEVGVYRLSKVLDEEVARLHLEKLGAKLTKMSQEQAEYIGFPVEGPYKPEHYRY

>*Mi*SAHH

MGSSHHHHHHSSGLVPRGSHMYKVKDINLWKEGERKIEWAKEHMPVLSLIRERFKEEKPFKGLTIGMALHLEAKTAVLAET
LMEGGAKIAITGCNPLSTQDDVAAACAKKGMHVYAWRGETREEYYENLNRVLDHEPDIIIDDGCDLIFLVHTKRTELIEKIRGAC
EETTTGIIRLKAMEKEGALKFPVMDVNDAYTKYLFDNRYGTGQSALDGIIRATNLLIAGKTVVVAGYGWCGRGVAMRAKGLGA
EVIVTEVNPIRALEARMDGFRVMKMEEAVKYGDIFITTTGCKDVIRKEHMVKMKDKAILANAGHFDNEINKRDLEELAEKIKEV
RNGVREYKLRDGKKLYLLGDGRLVNLVCADGHPCEVMDMSFSNQALAAEYIKEHYKELEPRVYRIPYEQDLMIAKLKLRSMGIE
IDELTEEQKKYLEDWREGT

>*Mj*SIHH

MGSSHHHHHHSSGLVPRGSHMYEVRDINLWKEGERKIQWAKQHMPVLNLIRERFKEEKPFKGITIGMALHLEAKTAVLAETL
MEGGAEIAITGCNPLSTQDDVAAACAKKGMHVYAWRGETVEEYYENLNKVLDHKPDIVIDDGCDLIFLLHTKRTELLDNIMGG
CEETTTGIIRLKAMEKEGALKFPVMDVNDAYTKHLFDNRYGTGQSALDGILRATNLLIAGKTVVVAGYGWCGRGVAMRAKGL
GAEVVVTEVNPIRALEARMDGFRVMKMEKAAEIGDIFITTTGCKDVIRKEHILKMRNGAILANAGHFDNEINKKHLEELAKSIKE
VRNCVTEYDLGNKKIYLLGEGRLVNLACADGHPCEVMDMSFANQALAAEYILKNHEKLEPRVYNIPYEQDLMIASLKLKAMGIE
IDELTKEQKKYLEDWREGT

>*Mma*SAHH

MGSSHHHHHHSSGLVPRGSHMSNVKDMSLAPSGHLKMEWAKRHMPVLCRIAEEFKNDKPFEGLTIGMALHLEAKTAILAET
LLEGGAKIVITGCNPLSTQDDVAAACVEKGMEVYAWRGETNEEYYENLNKVLDSNPDIIIDDGADLIFLIHTERTELIGKIMGGCE
ETTTGIIRLKSMAEEGALKFPVVNVNDAYTKHLFDNRYGTGQSAMDGIIRTTNLLIAGKNVVVGGYGWCGRGVASRAAGHGA
NVIITEVNPIRALEAKMDGFTVLKMEEAAKIGDIFVTTTGCKDILRMEHFLLMKDGAVLSNAGHFDNEINKNDLKELSKSVKEAR
FNIEEYDLGNKKIYLLGEGRLVNLACADGHPCEVMDMSFANQALSAKFIKENKGKLENEVYEIPYEQDFKIALLKLHSMGADIDE
LSPEQRKYLSDWKEGT

>*Mm*SAHH

MGSSHHHHHHSSGLVPRGSHMSDKLPYKVADIGLAAWGRKALDIAENEMPGLMRMREMYSASKPLKGARIAGCLHMTVET
AVLIETLVALGAEVRWSSCNIFSTQDHAAAAIAKAGIPVFAWKGETDEEYLWCIEQTLHFKDGPLNMILDDGGDLTNLIHTKYP
QLLSGIRGISEETTTGVHNLYKMMSNGILKVPAINVNDSVTKSKFDNLYGCRESLIDGIKRATDVMIAGKVAVVAGYGDVGKGC
AQALRGFGARVIITEIDPINALQAAMEGYEVTTMDEACKEGNIFVTTTGCVDIILGRHFEQMKDDAIVCNIGHFDVEIDVKWLN
ENAVEKVNIKPQVDRYWLKNGRRIILLAEGRLVNLGCAMGHPSFVMSNSFTNQVMAQIELWTHPDKYPVGVHFLPKKLDEAV
AEAHLGKLNVKLTKLTEKQAQYLGMPINGPFKPDHYRY

>*Mt*SAHH

MGSSHHHHHHSSGLVPRGSHMEKSIVKDITLADAGARRIEWARRHMPVLQLIKEEFERERPLEGIRIVACLHVTVETANLIEALR
AGGAEIALTGSNPLSTQDDVAAALASRGIHVYAFRGENESEYYECIERALELRPNVTLDDGADTIAVVHKSHRELIPEILGGCEETT
TGVMRLRAMADDGALAYPVIAVNDAETKMMFDNRYGTGQSTLHGIMNATNFLFAGKTVVVAGYGWCGRGFAMRAKGLG
ANVIVVETEPRKALEAAMDGYRVMSMRSAAPLGDLFVTLTGDINVIRNEHFELMKDQVILANSGHFNVEIDIPGLESMAVSTD
EIQPNVKEYVMPDGRRIYLLAEGRLINLAAAYGHPPEVMDMSFANQALSVRYIVENGRRLERKVYPVPVEIDRRVAELKLKAMG
IEIERLTPEQDRYLHSWQMGT

>*Pa*SAHH

MGSSHHHHHHSSGLVPRGSHMSAVMTPAGFTDYKVADITLAAWGRRELIIAESEMPALMGLRRKYAGQQPLKGAKILGCIH
MTIQTGVLIETLVALGAEVRWSSCNIFSTQDQAAAAIAAAGIPVFAWKGETEEEYEWCIEQTILKDGQPWDANMVLDDGGDL
TEILHKKYPQMLERIHGITEETTTGVHRLLDMLKNGTLKVPAINVNDSVTKSKNDNKYGCRHSLNDAIKRGTDHLLSGKQALVIG
YGDVGKGSSQSLRQEGMIVKVAEVDPICAMQACMDGFEVVSPYKNGINDGTEASIDAALLGKIDLIVTTTGNVNVCDANMLK
ALKKRAVVCNIGHFDNEIDTAFMRKNWAWEEVKPQVHKIHRTGKDGFDAHNDDYLILLAEGRLVNLGNATGHPSRIMDGSF
ANQVLAQIHLFEQKYADLPAAEKAKRLSVEVLPKKLDEEVALEMVKGFGGVVTQLTPKQAEYIGVSVEGPFKPDTYRY

>*Pfu*SAHH

MGSSHHHHHHSSGLVPRGSHMDCGKDYCVKDLSLAEEGWKKIDWVSRFMPVLQYIKREFEEKKPFKGVRIAATLHLEMKTAF
LLLTLKAGGAEVSAAASNPLSTQDDVVAALAKAGVKVYAIRGESREQYYEFMHKALDIRPNIIIDDGADMISLVHKERQEMLDEI
WGGSEETTTGVIRLRAMEKAGILKFPVIAVNDSYMKYLFDNRYGTGQSTWDGIMRATNLLIAGKNVVVVGYGWCGRGIAMR
ARGLGATVIVVEVDPIKALEARMDGFLVMDMKEAAKIGDIFVTATGNIKCIRREHFELMKDGAIMANAGHFDVEIWKPDLEKL
AVEINNPRPNVTEYKLKDGRRLYLLADGRLVNLVAADGHPAEIMDMSFALQAKAAEYIKDNHERLEPKVYILPREIDEMVARIKL
ESMGIKIEELTEEQKKYLESWEHGT

>*Sac*SAHH

MGSSHHHHHHSSGLVPRGSHMDYRVKDLSLAEQGRKQIEWAELHMPALMEIRKRFNAEKPLDGIRIGAVLHVTKETAVLVET
LKAGGAEIALAGSNPLSTQDDVAAGLAKNGIHVYAWRGETEKDYYDNIREILKYEPHVIMDDGGDLHAYVHENNLTSKIVGGT
EETTTGVIRLKAMEEEKVLKYPVIAVNNAFTKYLFDNRIGTGQSTIDGILRATNILIAGKVAVVIGYGWVGRGIASRFKGMGARVI
VVESSPFRALEALMDGFDVMTMNRASEIGDIFVTATGNLNVVSRDHILRMKDGAVLANSGHFNVEIDVKGLKEISVETREVRQ
NLEEYKLRNGKRIYLLADGRLVNLVAAEGHPSEVMDLSFCNQALSVEHLIKNKGKLENKVYNVPIEIDEQVARLKLKALGIEIEELTI
EQKEYIKQWKYGT

>*Sa*SAHH

MGSSHHHHHHSSGLVPRGSHMASAQQHDFKVADLSLAEFGRKEITLAEHEMPGLMSIREEYAASQPLAGARVTGSLHMTVQ
TAVLIETLTALGAEVRWASCNIFSTQDHAAAAIAVGPNGTPDNPQGVPVFAWKGESLEEYWWCTEQALTWPNTPTGGPNMI
LDDGGDATLLVHNGVQYEKDGKVPDPVTAESDEHRVILQLLTRTLGENPQKWTQLASEIRGVTEETTTGVHRLYEMQRDGQL
LFPAINVNDAVTKSKFDNKYGCRHSLIDGINRATDVLIGGKTAVVCGYGDVGKGCAESLRGQGARVIVTEIDPICALQAAMDGY
QVATLDDVIGQADIFVTTTGNKDIIMAADMAKMKHQAIVGNIGHFDNEIDMAGLAAIPGIVKDEVKPQVHTWTFPDGKVIIVL
SEGRLLNLGNATGHPSFVMSNSFADQTLAQIELFTKPDEYPTDVYVLPKHLDEKVARLHLDALGVRLTTLRPEQAAYIGVSVEGP
FKPDHYRY

>*Sf*SAHH

MGSSHHHHHHSSGLVPRGSHMTTTSTTGHDFKVADLSLAAFGRKEITLAEHEMPGLMAIRKEYSAEKPLAGARITGSLHMTVQTAVLIETLVA
LGAEVRWASCNIFSTQDHAAAAIAVGPDGTPDNPRGVPVFAWKGETLEEYWWCTEQALTWPNTPTGGPNMILDDGGDATLLVHKGVEYE
KAGAAPSVDTAENDEHRVILQLLNRTLAESPQKWTQPASEIRGVTEETTTGVHRLYEMQQAGTLLFPAINVNDAVTKSKFDNKYGCRHSLIDG
INRATDVLIGGKTAVVCGYGDVGKGCAESLRGQGARVMITEIDPICALQAAMDGYQVVRLDDVVETADIFITTTGNKDIIMASDMAKMKHQ
AIVGNIGHFDNEIDMAGLAAIDGIVKDEVKPQVHTWTWPDGKSIIVLSEGRLLNLGNATGHPSFVMSNSFANQTIAQIELFTKPESYPTDVYVL
PKHLDEKVARLHLDALGAKLTTLRPEQAAYIGVPVEGPYKPDHYRY

>*Sso*SAHH

MGSSHHHHHHSSGLVPRGSHMSYKIKDLSLASEGKKQIEWAERHMPTLMEIRKRFKAEKPLKGINISAVLHVTKETAALVKTLKI
GGANVALAGSNPLSTQDDVAAALVEEGISVFAWKGENETEYYSNIESIVKIHEPNIVMDDGADLHAYIHEKVSSKLDIYGGTEET
TTGVIRLKAMEKDGVLKYPLVAVNNAYTKYLFDNRYGTGQSAIDGILRATNILIAGKIAVVAGYGWVGRGIANRLRGMGARVIV
TEVDPIRALEAVMDGFDVMPIAEASKVGDIFVTATGNTKAIRVEHMLNMKDGAILSNAGHFNVEVDVKGLKETAVKVRNIRPY
VDEYTLPNGKRVYLLADGRLVNLAAAEGHPSEVMDMSFANQALAVEYLVKNRGKLEKKVYNMPMELDYEVARIKLKSMGIQI
DELTEEQKEYLEQWKSGT

>*Tk*SAHH

MGSSHHHHHHSSGLVPRGSHMDCTKDYCVKDISLAPSGEKKIDWVSRFMPVLQHIRKDFEERKPFKGVRIAATLHLEMKTAFL
LLTLKAAGAEVSAAASNPLSTQDDVVAALAKAGVKVYAIRGEDREQYYEFMHKALDVKPNIIIDDGADMVSTVLKERQELIPEI
WGASEETTTGVIRLRAMEKDGVLKFPIIAVNDSYTKYLFDNRYGTGQSTWDGIIRTTNLLVAGKNVVVVGYGWCGRGIAMRA
RGLGATVIVVEVDPIRALEARMDGFLVMDMMEAAKVGDIFITATGDINCIRKEHFELMKDGAILANAGHFDVEISKPDLEALAV
EISEPRPNITEYKMADGRRLYLLAEGRLVNLAAADGHPAEIMDMSFALQAKAAEYIKENRGRLEPKVYVLPREIDEMVARIKLAS
MGIKIEELTEEQKKYLESWEHGT

>*Tm*SAHH

MGSSHHHHHHSSGLVPRGSHMNTGEMKINWVSRYMPLLNKIAEEYSREKPLSGFTVGMSIHLEAKTAYLAITLSKLGAKVVIT
GSNPLSTQDDVAEALRSKGITVYARRTHDESIYRENLMKVLDERPDFIIDDGGDLTVISHTEREEVLENLKGVSEETTTGVRRLKA
LEETGKLRVPVIAVNDSKMKYLFDNRYGTGQSTWDAIMRNTNLLVAGKNVVVAGYGWCGRGIALRAAGLGARVIVTEVDPV
KAVEAIMDGFTVMPMKEAVKIADFVITASGNTDVLSKEDILSLKDGAVLANAGHFNVEIPVRVLEEIAVEKFEARPNVTGYTLEN
GKTVFLLAEGRLVNLAAGDGHPVEIMDLSFALQIFAVLYLLENHRKMSPKVYMLPDEIDERVARMKLDSLGVKIDELTEKQRRYL
RSWQ

References

1. Guranowski, A. & Pawelkiewicz, J. Adenosylhomocysteinase from Yellow Lupin Seeds. Purification and Properties. *Eur. J. Biochem.* **80**, 517–523 (1977).
2. Brzezinski, K., Bujacz, G. & Jaskolski, M. Purification, crystallization and preliminary crystallographic studies of plant *S* -adenosyl- L -homocysteine hydrolase (*Lupinus luteus*). *Acta Crystallogr. Sect. F Struct. Biol. Cryst. Commun.* **64**, 671–673 (2008).
3. Brzezinski, K., Dauter, Z. & Jaskolski, M. High-resolution structures of complexes of plant *S* -adenosyl- L -homocysteine hydrolase (*Lupinus luteus*). *Acta Crystallogr. D Biol. Crystallogr.* **68**, 218–231 (2012).
4. Miller, D., Xu, H. & White, R. H. S-Inosyl-L-homocysteine hydrolase, a novel enzyme involved in S-adenosyl-L-methionine recycling. *J. Bacteriol.* **197**, 2284–2291 (2015).
5. Ishihara, M. *et al.* Crystallization of mouse *S* -adenosyl- L -homocysteine hydrolase. *Acta Crystallogr. Sect. F Struct. Biol. Cryst. Commun.* **66**, 313–315 (2010).
6. Kusakabe, Y. *et al.* Structural insights into the reaction mechanism of S-adenosyl-L-homocysteine hydrolase. *Sci. Rep.* **5**, 16641 (2015).
7. Czyrko, J. *et al.* Metal-cation regulation of enzyme dynamics is a key factor influencing the activity of S-adenosyl-l-homocysteine hydrolase from Pseudomonas aeruginosa. *Sci. Rep.* **8**, 11334 (2018).
8. Porcelli, M. *et al.* S-adenosylhomocysteine hydrolase from the archaeon Pyrococcus furiosus: biochemical characterization and analysis of protein structure by comparative molecular modeling. *Proteins* **58**, 815–825 (2005).
9. Porcelli, M. *et al.* S-Adenosylhomocysteine hydrolase from the thermophilic archaeon Sulfolobus solfataricus: purification, physico-chemical and immunological properties. *Biochim. Biophys. Acta Prot. Struct. Mol. Enzymol.* **1164**, 179–188 (1993).
10. Porcelli, M., Fusco, S., Inizio, T., Zappia, V. & Cacciapuoti, G. Expression, purification, and characterization of recombinant S-adenosylhomocysteine hydrolase from the thermophilic archaeon Sulfolobus solfataricus. *Protein Expr. Purif.* **18**, 27–35 (2000).
11. Hermann, J. C. *et al.* Structure-based activity prediction for an enzyme of unknown function. *Nature* **448**, 775-779 (2007).
12. Lozada-Ramírez, J. D., Sánchez-Ferrer, A. & García-Carmona, F. Recombinant S-adenosylhomocysteine hydrolase from Thermotoga maritima: cloning, overexpression, characterization, and thermal purification studies. *Appl. Biochem. Biotechnol.* **170**, 639–653 (2013).
13. Manszewski, T., Singh, K., Imiolczyk, B. & Jaskolski, M. An enzyme captured in two conformational states: crystal structure of *S* -adenosyl- L -homocysteine hydrolase from *Bradyrhizobium elkanii*. *Acta Crystallogr.* D**71**, 2422–2432 (2015).
14. Manszewski, T., Szpotkowski, K. & Jaskolski, M. Crystallographic and SAXS studies of *S* -adenosyl- L -homocysteine hydrolase from *Bradyrhizobium elkanii*. *IUCrJ* **4**, 271–282 (2017).
15. Kailing, L. L. *et al.* S-Adenosyl-L-Homocysteine Hydrolase Inhibition by a Synthetic Nicotinamide Cofactor Biomimetic. *Front. Microbiol.* **9**, 505 (2018).
16. Turner, M. A. *et al.* Structure determination of selenomethionyl S-adenosylhomocysteine hydrolase using data at a single wavelength. *Nat. Struct. Mol. Biol.* **5**, 369–376 (1998).
17. Yang, X. *et al.* Catalytic Strategy of *S* -Adenosyl- L -homocysteine Hydrolase: Transition-State Stabilization and the Avoidance of Abortive Reactions. *Biochemistry* **42**, 1900–1909 (2003).
18. Lee, Y., Jeong, L. S., Choi, S. & Hyeon, C. Link between Allosteric Signal Transduction and Functional Dynamics in a Multisubunit Enzyme: *S* -Adenosylhomocysteine Hydrolase. *J. Am. Chem. Soc.* **133**, 19807–19815 (2011).
19. Nakao, A. *et al.* Discovery and structural analyses of S-adenosyl-l-homocysteine hydrolase inhibitors based on non-adenosine analogs. *Bioorg. Med. Chem.* **23**, 4952–4969 (2015).
20. Uchiyama, N. *et al.* Identification of AHCY inhibitors using novel high-throughput mass spectrometry. *Biochem. Biophys. Res. Commun.* **491**, 1–7 (2017).

21. Wang, Y. *et al.* Regulation of S-Adenosylhomocysteine Hydrolase by Lysine Acetylation. *J. Biol. Chem.* **289**, 31361–31372 (2014).
22. Reddy, M. C. M. *et al.* Crystal structures of *Mycobacterium tuberculosis* S-adenosyl-L-homocysteine hydrolase in ternary complex with substrate and inhibitors. *Protein Sci.* **17**, 2134-2144 (2008).
23. Tanaka, N. *et al.* Crystal Structure of S-Adenosyl-l-Homocysteine Hydrolase from the Human Malaria Parasite Plasmodium falciparum. *J. Mol. Biol.* **343**, 1007–1017 (2004).
24. Hu, Y. *et al.* Crystal Structure of *S* -Adenosylhomocysteine Hydrolase from Rat Liver. *Biochemistry* **38**, 8323–8333 (1999).
25. Huang, Y. *et al.* Inhibition of S-Adenosylhomocysteine Hydrolase by Acyclic Sugar Adenosine Analogue d-Eritadenine. *J. Biol. Chem.* **277**, 7477–7482 (2002).
26. Takata, Y. *et al.* Catalytic Mechanism of S-Adenosylhomocysteine Hydrolase. *J. Biol. Chem.* **277**, 22670–22676 (2002).
27. Yamada, T. *et al.* Catalytic mechanism of S-adenosylhomocysteine hydrolase: Roles of His 54, Asp130, Glu155, Lys185, and Aspl89. *Int. J. of Biochem. Cell Biol.* **37**, 2417–2435 (2005).
28. Malecki, P. H. *et al.* Biochemical and structural insights into an unusual, alkali-metal-independent *S* -adenosyl- L -homocysteine hydrolase from *Synechocystis* sp. PCC 6803. *Acta Crystallogr. D Struct. Biol.* **78**, 865–882 (2022).
29. Siponen, M. I. *et al.* S-adenosyl homocysteine hydrolase (SAHH) from Trypanosoma brucei. (2009) doi:10.2210/pdb3H9U/pdb.
30. Zheng, Y. *et al.* Crystal structures of S-adenosylhomocysteine hydrolase from the thermophilic bacterium Thermotoga maritima. *J. Struct. Biol.* **190**, 135–142 (2015).
31. Brzezinski, K. *et al.* S-adenosyl-L-homocysteine hydrolase from a hyperthermophile (*Thermotoga maritima*) is expressed in Escherichia coli in inactive form – Biochemical and structural studies. *Int. J. Biol. Macromol.* **104**, 584–596 (2017).
32. Altschul, S. F. *et al.* Gapped BLAST and PSI-BLAST: a new generation of protein database search programs. *Nucleic Acids Res.* **25**, 3389–3402 (1997).
33. Madeira, F. *et al.* Search and sequence analysis tools services from EMBL-EBI in 2022. *Nucleic Acids Res.* **50**, W276–W279 (2022).
34. Laemmli, U. K. Cleavage of structural proteins during the assembly of the head of bacteriophage T4. *Nature* **227**, 680–685 (1970).
35. Sievers, F. *et al.* Fast, scalable generation of high-quality protein multiple sequence alignments using Clustal Omega. *Mol. Syst. Biol.* **7**, 539 (2011).
36. Waterhouse, A. M., Procter, J. B., Martin, D. M. A., Clamp, M. & Barton, G. J. Jalview Version 2--a multiple sequence alignment editor and analysis workbench. *Bioinformatics* **25**, 1189–1191 (2009).
37. Kusakabe, Y., Ishihara, M. & Tanaka, N. Crystal structure of mouse SAHH complexed with adenosine. (2016) doi:10.2210/pdb5AXA/pdb.
