# Supplementary material for: Structure, function and substrate preferences of archaeal S-adenosyl-l-homocysteine hydrolases
Source: Commun Biol. 2024 Mar 29;7:380. doi: 10.1038/s42003-024-06078-9 (PMC10978960; doi:10.1038/s42003-024-06078-9)
Supplement: Supplementary file 3 — Description of additional supplementary files [file 42003_2024_6078_MOESM3_ESM.docx]

Description of Additional Supplementary Files

**File name:** Supplementary Data 1

**Description:** The source data behind the chromatograms in the paper and the Supplementary Information. Column A shows the time points in min, while column B includes the absorption data in mAU.
